# Estimating the cause-specific relative risks of non-optimal temperature on daily mortality: a two-part modelling approach applied to the Global Burden of Disease Study

**DOI:** 10.1016/S0140-6736(21)01700-1

**Published:** 2021-08-21

**Authors:** Katrin G Burkart, Michael Brauer, Aleksandr Y Aravkin, William W Godwin, Simon I Hay, Jaiwei He, Vincent C Iannucci, Samantha L Larson, Stephen S Lim, Jiangmei Liu, Christopher J L Murray, Peng Zheng, Maigeng Zhou, Jeffrey D Stanaway

**Affiliations:** a Institute for Health Metrics and Evaluation, University of Washington, Seattle, WA, USA; b Department of Health Metrics Sciences, School of Medicine, University of Washington, Seattle, WA, USA; c School of Population and Public Health, University of British Columbia, Vancouver, BC, Canada; d Non-Communicable Disease Center, Chinese Center for Disease Control and Prevention, Beijing, China

## Abstract

**Background:**

Associations between high and low temperatures and increases in mortality and morbidity have been previously reported, yet no comprehensive assessment of disease burden has been done. Therefore, we aimed to estimate the global and regional burden due to non-optimal temperature exposure.

**Methods:**

In part 1 of this study, we linked deaths to daily temperature estimates from the ERA5 reanalysis dataset. We modelled the cause-specific relative risks for 176 individual causes of death along daily temperature and 23 mean temperature zones using a two-dimensional spline within a Bayesian meta-regression framework. We then calculated the cause-specific and total temperature-attributable burden for the countries for which daily mortality data were available. In part 2, we applied cause-specific relative risks from part 1 to all locations globally. We combined exposure–response curves with daily gridded temperature and calculated the cause-specific burden based on the underlying burden of disease from the Global Burden of Diseases, Injuries, and Risk Factors Study, for the years 1990–2019. Uncertainty from all components of the modelling chain, including risks, temperature exposure, and theoretical minimum risk exposure levels, defined as the temperature of minimum mortality across all included causes, was propagated using posterior simulation of 1000 draws.

**Findings:**

We included 64·9 million individual International Classification of Diseases-coded deaths from nine different countries, occurring between Jan 1, 1980, and Dec 31, 2016. 17 causes of death met the inclusion criteria. Ischaemic heart disease, stroke, cardiomyopathy and myocarditis, hypertensive heart disease, diabetes, chronic kidney disease, lower respiratory infection, and chronic obstructive pulmonary disease showed J-shaped relationships with daily temperature, whereas the risk of external causes (eg, homicide, suicide, drowning, and related to disasters, mechanical, transport, and other unintentional injuries) increased monotonically with temperature. The theoretical minimum risk exposure levels varied by location and year as a function of the underlying cause of death composition. Estimates for non-optimal temperature ranged from 7·98 deaths (95% uncertainty interval 7·10–8·85) per 100 000 and a population attributable fraction (PAF) of 1·2% (1·1–1·4) in Brazil to 35·1 deaths (29·9–40·3) per 100 000 and a PAF of 4·7% (4·3–5·1) in China. In 2019, the average cold-attributable mortality exceeded heat-attributable mortality in all countries for which data were available. Cold effects were most pronounced in China with PAFs of 4·3% (3·9–4·7) and attributable rates of 32·0 deaths (27·2–36·8) per 100 000 and in New Zealand with 3·4% (2·9–3·9) and 26·4 deaths (22·1–30·2). Heat effects were most pronounced in China with PAFs of 0·4% (0·3–0·6) and attributable rates of 3·25 deaths (2·39–4·24) per 100 000 and in Brazil with 0·4% (0·3–0·5) and 2·71 deaths (2·15–3·37). When applying our framework to all countries globally, we estimated that 1·69 million (1·52–1·83) deaths were attributable to non-optimal temperature globally in 2019. The highest heat-attributable burdens were observed in south and southeast Asia, sub-Saharan Africa, and North Africa and the Middle East, and the highest cold-attributable burdens in eastern and central Europe, and central Asia.

**Interpretation:**

Acute heat and cold exposure can increase or decrease the risk of mortality for a diverse set of causes of death. Although in most regions cold effects dominate, locations with high prevailing temperatures can exhibit substantial heat effects far exceeding cold-attributable burden. Particularly, a high burden of external causes of death contributed to strong heat impacts, but cardiorespiratory diseases and metabolic diseases could also be substantial contributors. Changes in both exposures and the composition of causes of death drove changes in risk over time. Steady increases in exposure to the risk of high temperature are of increasing concern for health.

**Funding:**

Bill & Melinda Gates Foundation.

**Research in context**

**Evidence before this study**

Ecological studies have evaluated all-cause mortality or aggregate causes of temperature-associated mortality, such as cardiorespiratory mortality, but with few studies assessing detailed cause-specific data. Previous studies have focused primarily on temperature-related excess mortality in individual cities rather than larger geographical areas, including rural locations. No consistent approach has been applied to estimate the global and regional burden due to non-optimal temperature exposure.

**Added value of this study**

This study assessed the relationship between cause-specific mortality and daily temperature on a dataset of 64·9 million individual deaths from nine countries. This spanned 29% of the global population as well as approximately 95% of the inhabited global temperature range, and 79% of sociodemographic conditions. Our analysis extends those focused on a more limited set of mortality causes or limited to specific locations. Our global extrapolation allowed us to estimate temperature-attributable burden, with appropriate uncertainty intervals, in areas for which no daily mortality data are available by accounting for cause-specific temperature sensitivity and the underlying burden of disease in a location. Previous studies have highlighted large heterogeneity of temperature across different locations. Our meta-regression—Bayesian, regularised, trimmed tool—accounts for between-location differences and incorporates them into the uncertainty range.

**Implications of all the available evidence**

We provide the first global cause-specific temperature-attributable burden estimates developed with an internally consistent approach. This offers a valuable tool to assist public health policy making and intervention planning, and for the first time allows temperature effects to be incorporated into the Global Burden of Disease framework as a prelude to investigating future temperature scenarios. This study highlights the relevance of temperature as a risk factor for human health and identifies various causes and areas that are particularly affected. We show that the burden attributable to high and low temperature is strongly driven by the location-specific underlying burden of disease. Although in most locations a higher disease burden can be attributed to low temperature exposure, the heat-related burden is substantial in countries with high prevailing temperatures, such as Brazil, Colombia, and Guatemala, and specifically in certain areas within these countries. Our extrapolation model provides initial temperature-attributable burden estimations for many countries and regions for which no estimations were previously available. Areas with high prevailing ambient temperatures, such as south Asia, North Africa and the Middle East, and sub-Saharan Africa, show a particularly high heat-attributable burden. Our observation that the risk exposure to high temperatures increased from 1990 to 2019, while the risk exposure to low temperatures only marginally declined over the same time period, suggests a growing future relevance of the disease burden related to heat. Given rising temperatures due to climate change, heat effects might become particularly pronounced in areas that are already hot.

## Introduction

Climate change is expected to affect human health directly by increasing exposure to extreme temperatures and indirectly by various pathways such as sea level rise, extreme weather events, migration, and changes in agricultural productivity resulting in rural poverty.^1^, ^2^ The excess mortality associated with direct and acute effects of short-term exposure to extreme temperatures is among the best documented effects of climate on human health, yet its attributable disease burden has yet to be comprehensively estimated.^3^, ^4^, ^5^ Studies have noted an increased risk of morbidity and mortality associated with ambient temperatures that are either colder or warmer than an optimum temperature.^3^, ^4^, ^5^ One study attributed 7·7% of mortality to non-optimum temperature in 13 selected countries spanning various climate zones and sociodemographic conditions.^4^ Although cold effects generally outweigh heat effects on health, with variations across different locations and regions, the detrimental nature of heat becomes particularly evident in regions with hot climates or during heatwaves.^3^, ^4^, ^5^, ^6^, ^7^, ^8^

To date, research has focused primarily on temperature effects in individual cities,^4^, ^5^, ^6^, ^7^, ^8^, ^9^ and has not produced comprehensive global estimates to predict temperature-attributable burden across all locations. Deviations from a set core temperature can trigger a wide array of adverse biological and biomedical reactions,^10^, ^11^ yet, most studies have evaluated the effects of temperature only on all-cause mortality or broader categories of cardiorespiratory mortality; analyses of a range of detailed causes of death are scarce.

We apply a two-part modelling approach. In the first stage, we analyse the temperature-mortality relationships and estimated cause-specific exposure–response surfaces along temperature zone (ie, the daily mean temperature, for each day, and the mean temperature across all days) and daily mean temperature deaths from nine countries. In the second stage of the analysis, we estimated mortality population attributable fractions (PAFs) specific to location, year, and cause, and attributable burden (disability-adjusted life-years [DALYs]) with appropriate uncertainty bounds, for 204 countries and territories for 1990–2019. To our knowledge, no previous study has analysed the associations between ambient temperature and a comprehensive collection of detailed causes of death, across geographies that span a wide range of climate and socioeconomic development scenarios.

## Methods

### Part 1: Daily temperature and cause-specific mortality and estimating the attributable burden of disease

#### Data

We used temperature estimates from ERA5, a gridded reanalysis dataset produced by the European Centre for Medium Range Weather Forecasts with 0·25° × 0·25° spatial and subdaily temporal resolutions, including uncertainty estimates on a 0·5° × 0·5° spatial and three-hourly resolution.^12^ Using temperature estimates for the period from Jan 1, 1980, to Dec 31, 2019, for each pixel we calculated the temperature zone, as previously described*.* We derived gridded population data from the WorldPop project for the years 2000, 2005, 2010, 2015, and 2020.^13^ To produce annual population estimates, we interpolated between 5-year bins and extrapolated by using the 2000–05 growth rate for back-casting until 1990.

Our analysis used daily cause-specific mortality data from the countries where individual deaths with information on International Classification of Diseases (ICD) code, administrative unit, and day were available based on publicly available datasets or through the Global Burden of Disease (GBD) Study collaborators. The data were collected at the administrative level of municipalities or counties. We mapped ICD codes to GBD causes; where deaths were coded to so-called garbage codes, we applied individual-level garbage-code redistribution based on GBD 2019 cause of death redistribution methods (appendix p 14).^14^, ^15^

For each death, we ascertained the temperature zone of location of the death, the daily mean temperature for the day, and location in which the death occurred. To avoid fitting to overly influential and noisy datapoints at extreme temperatures with few data, and to avoid extrapolations beyond the range of temperatures that correspond to the locations in our mortality data, we truncated temperature zones to the 1st and 99th percentiles of those in our mortality dataset; and we truncated daily mean temperatures to the 1st and 99th percentiles of daily temperatures within each temperature zone. We then calculated cause-specific mortality rates for each GBD level 3 cause of death, and for each combination of administrative level 2 location (eg, county or municipality) and daily mean temperature (appendix pp 14–15).

#### Relative risk (RR) modelling

**Figure 1:**
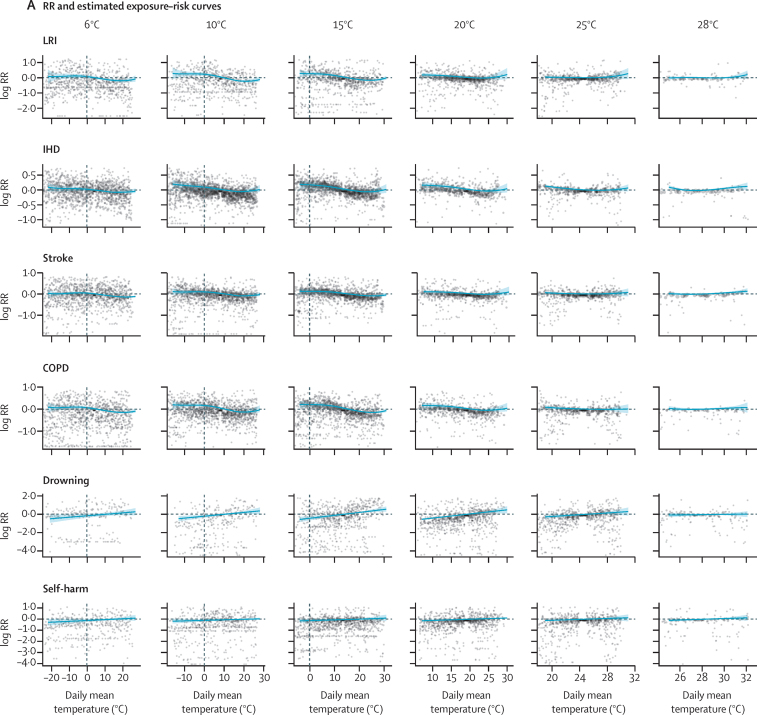

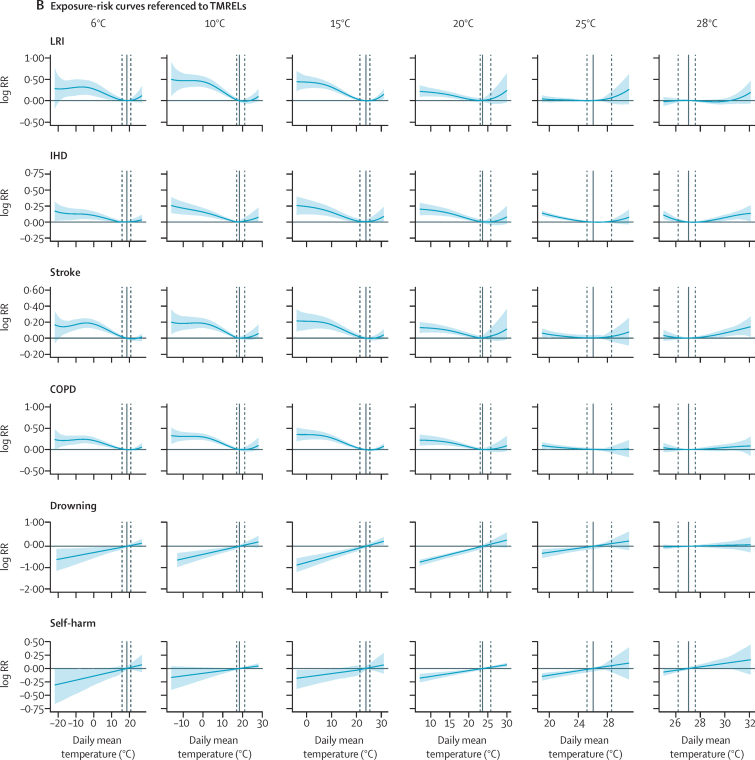
Exposure–response curves displaying the relationship between daily mean temperature and the log RR for mortality from LRI, IHD, stroke, COPD, drowning, and self-harm by temperature zone (A) RR estimates per daily mean temperature category and estimated exposure–risk curves. (B) Estimated exposure–risk functions. The RR is referenced to the TMREL, which represents the minimum mortality temperature for death-weighted multicause curves in each mean annual temperature category. The grey solid line depicts the location of the TMREL in each mean annual temperature category, with dashed lines depicting 95% UI of the TMREL. RR=relative risk. LRI=lower respiratory infections. IHD=ischaemic heart disease. COPD=chronic obstructive pulmonary disease. TMREL=theoretical minimum-risk exposure level. UI=uncertainty interval.

To estimate outcomes based on daily mean temperature and temperature zone, we used a robust meta-regression framework, implemented through the meta-regression—Bayesian, regularised, trimmed tool, known as MR-BRT.^16^ The tool allows three features that are essential to the analysis. First, a meta-analytic framework that can handle heterogeneous data sources. Modelling along different temperature zones and integrating data from all locations into one model allowed us to stabilise our estimates across zones. Second, a robust approach to outlier detection and removal using trimming methodology, which is standard in a vast array of inference and machine learning problems.^17^, ^18^, ^19^ Third, specification of the functional dependence of outcome versus daily mean temperature and temperature zone as a two-dimensional surface through a two-dimensional spline interface.^16^ For exposure–response relationships that depicted a J-shape, we fixed the curve to monotonically decrease or increase beyond the threshold temperature. For exposure–response relationships that displayed a one-directional relationship (ie, all external causes), we imposed monotonicity over the entire exposure range. Figure 1 displays raw RR and modelled splines (appendix p 15).

#### Cause selection

Starting with a list of all (n=176) level 3 causes in the GBD cause hierarchy, we first reduced the set of potential causes by excluding those causes (n=44) for which no deaths were recorded in our mortality dataset. We also excluded two causes for which vital registration data are not used to model mortality due to known inconsistencies in classification practices across countries (ie, dementia and protein-energy malnutrition). We estimated RR surfaces and uncertainties for all remaining (n=130) level 3 causes in the GBD cause hierarchy. For each cause, we calculated the magnitude of the RR function using methods developed for evaluating risk-outcome pairs in the GBD study and included all causes with a risk-outcome score above zero. A table with average risk-outcome scores for individual causes is provided in the appendix (p 17), additional information on risk-outcome score derivation is provided in the appendix (p 15).

#### Applying risk curves to estimates of the burden of disease

We estimated burden attributable to daily non-optimal temperature using the comparative risk assessment metric framework developed by Murray and Lopez.^20^ This approach uses four inputs: (1) the exposure levels for the risk factor, (2) the RR of the outcome as a function of exposure, (3) the counterfactual level of risk factor exposure, or theoretical minimum-risk exposure level (TMREL), and (4) the estimate of the burden being assessed for the cause (ie, number of deaths, years of life lost, years lived with disability, or DALYs).^20^

To estimate risk-attributable burden, we established a counterfactual level of exposure that was associated with the lowest overall burden across all causes that were included. Specifically, the TMREL was the location-specific daily temperature associated with the lowest mortality rates for all included causes combined. As comprehensive cause of death estimates do not exist at a finer spatial resolution than GBD locations, we assumed that the cause composition of deaths was consistent across all pixels within a given GBD national or subnational location (appendix pp 15–16).

We calculated the PAF (ie, the proportion of cause-specific deaths attributable to high or low daily temperatures), for each pixel and day, based on the RR associated with a given daily mean temperature within a given temperature zone. We calculated PAFs for high and low daily temperatures above and below the TMREL. The non-optimal temperature PAF is an aggregate of the high-temperature and low-temperature PAFs. We used estimates of cause-specific burden from the GBD study^15^ and estimated the temperature-attributable burden for each cause as the product of the total burden for that cause and the corresponding PAF for each GBD location, year, age group, and sex. We propagated uncertainty from all components of the modelling chain using posterior simulation: for each quantity of interest, we used 1000 random draws from the posterior distribution of the estimate and analysed all calculations at the draw level to generate 95% uncertainty intervals (UIs; appendix p 16).

The summary exposure values (SEVs) are measures of risk-weighted prevalence of exposure and range from 0% to 100%.^21^ A SEV of 0% reflects no risk exposue while 100% indicates that the entire population is exposed to the maximum level for that risk (RR_max_). For each cause and temperature zone, we estimated RR_max_ as the 99th percentile of RRs experienced for that cause and in that zone, across all years and locations, weighted by person-days of exposure. We then calculated the SEV for each cause, temperature zone, GBD location, and year (appendix pp 16–17).

### Part 2: global estimations of the temperature-attributable burden of disease

For generating global estimations, we applied the derived cause-specific exposure–response curves to a grid of global daily temperature exposure data from the ERA5 dataset. We calculated the PAF for each temperature pixel and population weighted pixels before aggregating to the country level. The exposure–response curves were truncated at the outer ends of the temperature distribution observed in locations included in the mortality dataset. These truncations meant that the RR was kept constant for maximum and minimum mean annual and daily temperature. For instance, for areas with a mean annual temperature below 6°C or above 28°C, the exposure–response curves for 6°C and 28°C were applied. Similarly, if daily mean temperatures in the extrapolation areas were below or above the daily mean temperatures within the locations with mortality, the RR at the maximum range of the curve was applied. The calculation of PAFs, TMRELs, and the attributable burden of disease was carried out analogously to the estimations for within-sample locations as described earlier.

This study complies with the Guidelines for Accurate and Transparent Health Estimates Reporting recommendations (appendix p 7). RRs were calculated in R and Python, PAFs and TMRELs were calculated in R, and burden of disease was calculated in Python.

### Role of the funding source

The funders of the study had no role in study design, data collection, data analysis, data interpretation, or writing of the report.

## Results

For part 1 of our analyses, which spanned from Jan 1, 1980, to Dec 31, 2016, we used our mortality dataset of 64·9 million deaths from nine countries: Brazil, Chile, China, Colombia, Guatemala, Mexico, New Zealand, South Africa, and the USA. Locations with mortality data were in 23 temperature zones ranging from 6°C to 28°C (median 14°C, IQR 8–17); with daily mean temperatures ranging from –29°C to 33°C (median 12°C, IQR 3–21). These data represent a range of sociodemographic conditions and span the distribution of global temperatures, with only 5% of the global population residing in zones outside of the temperature range (mean temperature zones from 6°C to 28°C) represented in these data. Of the 176 causes that we specifically evaluated, 17 met the significance threshold to be included in the full analysis. These were a mix of non-external causes of cardiorespiratory (ischaemic heart disease, stroke, hypertensive heart disease, cardiomyopathy and myocarditis, lower respiratory infection, chronic obstructive pulmonary disease) and metabolic (diabetes and chronic kidney disease) groupings, and external causes (homicide, suicide, drowning, mechanical injuries and other unintentional injuries, animal-related, disaster-related, and road injuries and other transport-related injuries; appendix p 17).

For exposure–response curves, we observed two distinct shapes of the temperature-mortality relationship: first, non-external causes generally showed a J-shaped curve with increasing mortality below and above threshold temperature, and second, external causes displayed a monotonic increase in risk with increasing temperature (figure 1). For causes with J-shaped risk curves, the cold effects generally occur over a wide range of temperatures, whereas the heat effects tend to occur at only the upper end of the temperature range and were not observed for every cause and within every temperature zone. Complete risk surface plots and curves for all causes and all mean annual temperature categories are provided in the appendix (pp 20–30). For most causes, we observed evidence of adaptation, with risk curves varying by temperature zone. Global maps of the TMREL, derived as death-weighted averages of individual exposure–response curves, are provided in the appendix (p 9).

In all nine countries included in the pooled daily mortality datasets, the burden attributable to low temperatures exceeded the burden attributable to high temperatures (table). For Chile and New Zealand, we observed negligible heat effects. Cold effects were most pronounced in China with PAFs of 4·3% (95% UI 3·9–4·7) and attributable rates of 32·0 deaths (95% UI 27·2–36·8) per 100 000 and in New Zealand with 3·4% (2·9–3·9) and 26·4 deaths (22·1–30·2) per 100 000. Similarly, strong cold effects and high mortality rates (per 100 000) were observed in the USA (PAF of 3·4% [3·0–3·7] and 30·9 deaths [27·3–33·7]), and Chile (3·3% [2·9–3·8] and 20·6 deaths [17·8–23·4]). Moderately strong cold effects of PAFs and attributable rates of death per 100 000 were seen in Mexico (PAF of 2·2% [2·0–2·5] and 13·2 deaths [11·1–15·4]), South Africa (1·6% [1·4–1·8] and 15·1 deaths [13·3–16·9]), and Colombia (1·6% [1·3–1·9] and 8·06 deaths [5·98–10·4]). In 2019, cold effects were less pronounced in Guatemala with PAFs of 1·1% (0·9–1·3) and attributable rates of 5·76 deaths (4·33–7·66) per 100 000, and in Brazil with 0·8% (0·7–0·9) and 5·27 deaths (4·52–6·09). Heat effects were most pronounced in China with PAFs of 0·4% (0·3–0·6) and attributable rates of 3·25 deaths (2·39–4·24) per 100 000 and in Brazil with 0·4% (0·3–0·5) and 2·71 deaths (2·15–3·37). Overall, estimates for non-optimal temperature ranged from 7·98 deaths (7·10–8·85) per 100 000 and a PAF of 1·2% (1·1–1·3) in Brazil to 35·1 deaths (29·9–40·3) per 100 000 and a PAF of 4·7% (4·3–5·1) in China.

**Table:** PAF and burden estimates for high temperature, low temperature, and non-optimal temperature exposure by country in 1990 and 2019

	**1990**			**2019**		
	Attributable deaths (95% UI)	PAFs (%)	Attributable rate, per 100 000	Attributable deaths	PAFs (%)	Attributable rate, per 100 000
**High temperature**						
Brazil	2340 (1707 to 3023)	0·24 (0·17 to 0·31)	1·57 (1·15 to 2·03)	5881 (4658 to 7305)	0·42 (0·33 to 0·51)	2·71 (2·15 to 3·37)
Chile	1 (0 to 7)	0 (0 to 0·01)	0·009 (0 to 0·052)	5 (2 to 16)	0 (0 to 0·01)	0·03 (0·009 to 0·086)
China	31 696 (24 483 to 40 627)	0·38 (0·30 to 0·47)	2·68 (2·07 to 3·43)	46 224 (33 932 to 60 317)	0·43 (0·33 to 0·55)	3·25 (2·39 to 4·24)
Colombia	393 (271 to 557)	0·23 (0·16 to 0·33)	1·21 (0·83 to 1·71)	976 (688 to 1304)	0·40 (0·31 to 0·49)	2·04 (1·44 to 2·73)
Guatemala	42 (20 to 80)	0·06 (0·03 to 0·11)	0·53 (0·25 to 1·01)	234 (166 to 307)	0·25 (0·19 to 0·30)	1·32 (0·94 to 1·73)
Mexico	943 (715 to 1175)	0·21 (0·16 to 0·27)	1·10 (0·84 to 1·37)	2655 (1844 to 3533)	0·36 (0·26 to 0·46)	2·13 (1·48 to 2·83)
New Zealand	2 (0 to 5)	0·01 (0 to 0·02)	0·046 (−0·003 to 0·16)	2 (0 to 7)	0·01 (0 to 0·02)	0·047 (0·005 to 0·15)
South Africa	167 (86 to 264)	0·06 (0·03 to 0·09)	0·45 (0·23 to 0·72)	453 (275 to 671)	0·09 (0·05 to 0·13)	0·81 (0·50 to 1·21)
USA	6128 (4074 to 8621)	0·29 (0·19 to 0·40)	2·42 (1·61 to 3·40)	9854 (7333 to 13 046)	0·33 (0·25 to 0·44)	3·00 (2·24 to 3·98)
**Low temperature**						
Brazil	9117 (8027 to 10 326)	0·93 (0·81 to 1·05)	6·13 (5·39 to 6·94)	11 420 (9795 to 13 202)	0·81 (0·70 to 0·93)	5·27 (4·52 to 6·09)
Chile	2883 (2517 to 3268)	3·80 (3·33 to 4·30)	21·71 (18·96 to 24·61)	3754 (3235 to 4264)	3·32 (2·87 to 3·77)	20·63 (17·78 to 23·43)
China	344 273 (300 340 to 384 708)	4·12 (3·70 to 4·49)	29·08 (25·37 to 32·50)	455 735 (386 354 to 523 890)	4·28 (3·88 to 4·66)	32·04 (27·16 to 36·83)
Colombia	2047 (1669 to 2466)	1·21 (0·98 to 1·45)	6·29 (5·13 to 7·58)	3850 (2855 to 4963)	1·56 (1·27 to 1·88)	8·06 (5·98 to 10·39)
Guatemala	898 (729 to 1129)	1·23 (1·03 to 1·52)	11·27 (9·15 to 14·17)	1024 (770 to 1362)	1·08 (0·92 to 1·31)	5·76 (4·33 to 7·66)
Mexico	7977 (7178 to 8843)	1·81 (1·63 to 2·00)	9·33 (8·40 to 10·34)	16 447 (13 873 to 19 237)	2·23 (1·99 to 2·47)	13·16 (11·10 to 15·40)
New Zealand	1070 (927 to 1217)	4·08 (3·53 to 4·64)	31·32 (27·13 to 35·62)	1189 (994 to 1359)	3·45 (2·90 to 3·95)	26·45 (22·12 to 30·22)
South Africa	5426 (4 612 to 6284)	1·85 (1·58 to 2·11)	14·73 (12·52 to 17·06)	8372 (7390 to 9399)	1·61 (1·42 to 1·80)	15·06 (13·29 to 16·91)
USA	77 365 (69 254 to 84 114)	3·63 (3·25 to 3·95)	30·51 (27·31 to 33·17)	101 292 (89 621 to 110 435)	3·44 (3·03 to 3·75)	30·88 (27·33 to 33·67)
**Non-optimal temperature**						
Brazil	11 474 (10 312 to 12 693)	1·17 (1·05 to 1·28)	7·71 (6·93 to 8·53)	17 286 (15 386 to 19 185)	1·23 (1·10 to 1·35)	7·98 (7·10 to 8·85)
Chile	2885 (2519 to 3270)	3·80 (3·33 to 4·30)	21·72 (18·97 to 24·62)	3759 (3244 to 4267)	3·32 (2·88 to 3·77)	20·66 (17·83 to 23·45)
China	375 343 (327 344 to 417 900)	4·50 (4·06 to 4·85)	31·71 (27·65 to 35·31)	499 605 (425 094 to 573 429)	4·69 (4·30 to 5·06)	35·13 (29·89 to 40·32)
Colombia	2462 (2050 to 2907)	1·45 (1·21 to 1·72)	7·56 (6·30 to 8·93)	4820 (3669 to 6148)	1·96 (1·65 to 2·27)	10·01 (7·68 to 12·87)
Guatemala	942 (765 to 1179)	1·29 (1·08 to 1·59)	11·82 (9·60 to 14·79)	1258 (976 to 1637)	1·33 (1·15 to 1·58)	7·08 (5·49 to 9·21)
Mexico	8924 (8104 to 9810)	2·03 (1·85 to 2·23)	10·44 (9·48 to 11·48)	19 055 (16 111 to 22 083)	2·58 (2·32 to 2·83)	15·25 (12·89 to 17·68)
New Zealand	1072 (930 to 1218)	4·08 (3·54 to 4·64)	31·37 (27·21 to 35·64)	1191 (996 to 1361)	3·45 (2·91 to 3·95)	26·49 (22·16 to 30·27)
South Africa	5595 (4795 to 6405)	1·90 (1·64 to 2·17)	15·19 (13·02 to 17·39)	8815 (7846 to 9823)	1·69 (1·50 to 1·89)	15·86 (14·11 to 17·67)
USA	83 196 (75 143 to 89 943)	3·90 (3·52 to 4·22)	32·81 (29·63 to 35·47)	110 588 (98 410 to 119 580)	3·75 (3·35 to 4·06)	33·72 (30·00 to 36·46)

PAF=Population attributable fraction. UI=uncertainty interval.

**Figure 2:**
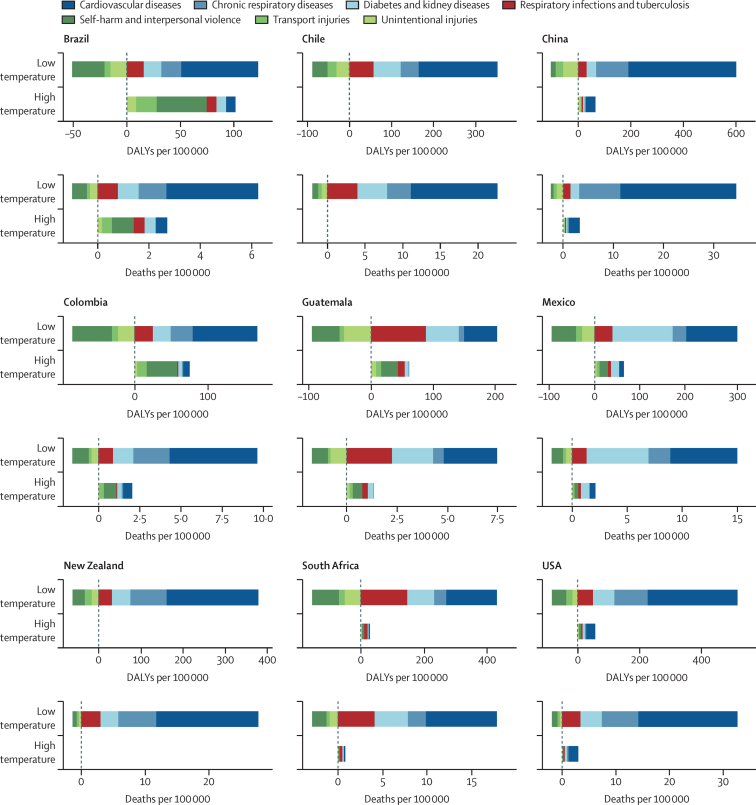
Composition of deaths and DALYs attributable to high and low temperatures in 2019 by level 2 GBD causes The x-axis scales vary by location. High and low temperatures are defined as the temperatures above (heat) and below (cold) the location and year-specific theoretical minimum risk exposure level. Cardiovascular diseases were ischaemic heart disease, stroke, hypertensive heart disease, and cardiomyopathy and myocarditis. Chronic respiratory diseases was chronic obstructive pulmonary disease. Respiratory infections and tuberculosis was lower respiratory infections. Diabetes and kidney diseases were diabetes and chronic kidneydisease. Self-harm and interpersonal violence were homicide and suicide. Transport injuries were road injuries and other transport-related injuries. Unintentional injuries were drowning, mechanical injuries and other unintentional injuries, animal-related, and disaster-related injuries. DALYs=disability-adjusted life-years. GBD=Global Burden of Disease.

Cold effects were largely driven by cardiorespiratory and metabolic disease (figure 2; appendix p 17). For external causes (eg, suicide, homicide, and injuries), cold temperatures always displayed a protective effect, leading to a negative cold-attributable burden. These protective effects were particularly evident in South Africa with –2·79 deaths (95% UI –3·49 to –2·13) per 100 000, in China with –2·46 deaths (–2·98 to –2·00), and in Chile with –1·92 deaths (–2·50 to –1·40). Although, Chile and New Zealand exhibited negligible heat-related burden, the relevance of heat-attributable burden increased for other countries. External causes contributed substantially to the heat-attributable burden, particularly when measuring DALYs (figure 2). Lower respiratory infections, chronic respiratory disease, cardiovascular disease, chronic kidney disease, and diabetes were other substantial contributors. We observed strong variations in heat-attributable and cold-attributable burden within countries. Appendix (p 30) highlights differences in cause composition of heat and cold effects for selected subnational locations in the USA, Brazil, and Mexico, which showed strong subnational differences. Additional information on these differences is provided in the appendix (pp 30–31).

**Figure 3:**
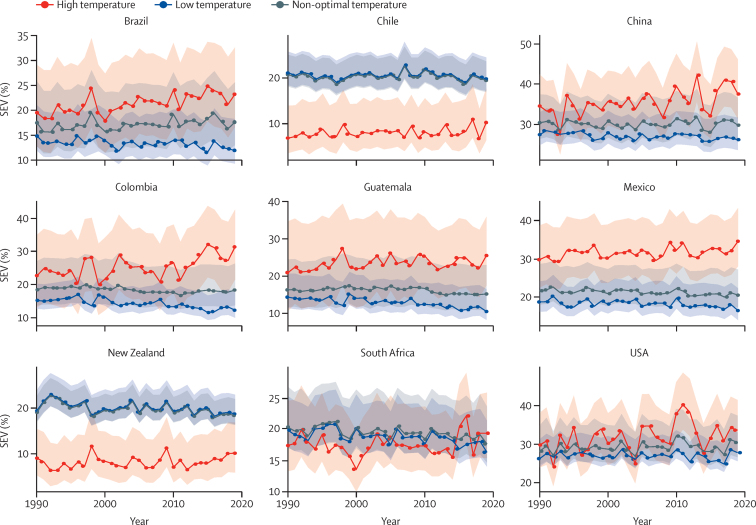
Time series plots from 1990 to 2019 of high and low temperature SEVs SEVs are a measure of risk-weighted prevalence for high and low temperature exposure and range from 0% to 100%. A SEV of 0% reflects no risk exposure, while 100% indicates that the entire population is exposed to the maximum possible level for that risk. The shaded areas indicate 95% UIs. SEV=summary exposure value. UI=uncertainty interval.

Results for temporal changes in risk exposure are shown in figure 3, which displays yearly SEVs for 1990 to 2019 for the nine countries considered in part 1 of this study. SEVs are a measure of risk-weighted prevalence of exposure for high and low temperatures and range from 0% to 100%. The indicator showed a strong fluctuation from year to year, reflecting inter-annual variations in temperature exposure, especially for high temperature SEVs. Although overall cold SEVs remained constant from 1990 to 2019, heat SEVs steadily increased over this time.

**Figure 4:**
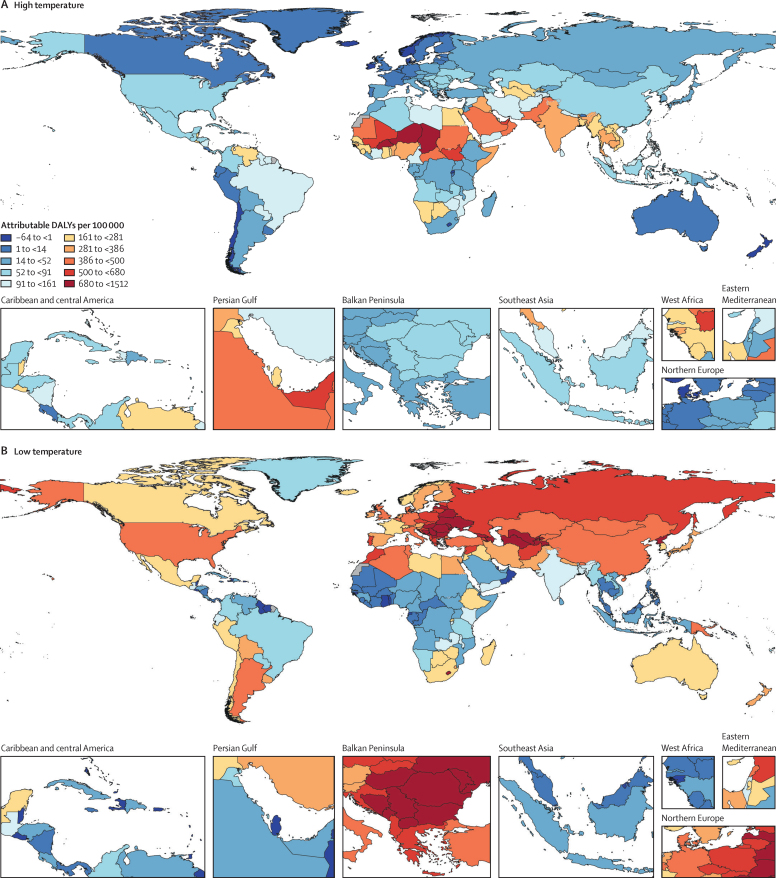

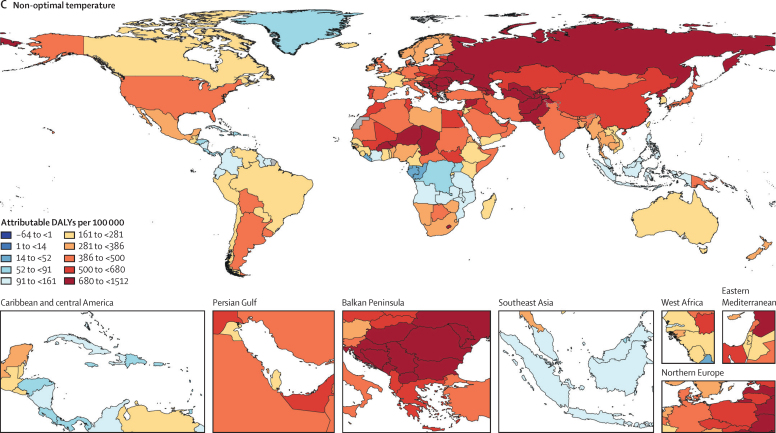
Spatial distribution of all-cause DALYs (per 100 000) attributable to high temperature (A), low temperature (B), and non-optimal temperature (C) exposure in 2019 DALYs=disability-adjusted life-years.

For part 2 of the analysis on global estimates, figure 4 displays the heat, cold, and non-optimal temperature attributable burden in 204 countries and territories. We estimated that 1·69 million (1·52–1·83) deaths were attributable to non-optimal temperature globally in 2019. The GBD regions of sub-Saharan Africa, North Africa and the Middle East, and south and southeast Asia display particularly high heat-attributable burden. This burden is largely driven by external as well as cardiorespiratory and metabolic disease. In sub-Saharan Africa, lower respiratory infections substantially contribute to the high heat-attributable burden. Wide regions of the middle and higher latitudes of the northern hemisphere display a high cold-attributable burden. Particularly, North America, eastern and central Europe, North Africa and the Middle East, and central and east Asia show substantial cold impacts. In the southern hemisphere, Argentina, Bolivia, Chile, Uruguay, Lesotho, Papua New Guinea, and New Zealand exhibit strong cold effects. This cold-related burden is largely driven by cardiovascular disease, chronic respiratory disease, metabolic disease, and acute respiratory infections. The relative contribution of different causes varies by location. For instance, in eastern Europe, cardiovascular disease was the largest contributor, whereas in Argentina, acute respiratory disease contributed a larger share as compared with other locations. Cause-specific maps for high, low, and non-optimal temperature PAFs and burdens are provided in the appendix (pp 30–35). In addition, burden estimates for high temperature, low temperature, and non-optimal temperature exposure by super-region and by cause are provided in the appendix (pp 32–34).

## Discussion

This temperature-mortality analysis is based on a multicountry dataset across a comprehensive collection of detailed mortality causes, and is the first to translate derived exposure–risk functions into estimates of the associated burden of disease in a comparative framework. Our study was separated into two parts. In the first part, we estimated the PAFs and attributable burden for nine countries and subnational locations for which we had access to daily cause-specific mortality data. These findings allowed insights into the cause of death composition of temperature-attributable deaths. Our analysis particularly highlighted the relevance of cardiorespiratory and metabolic mortality, as well as external causes of death such as interpersonal violence, self-harm, drowning, and injuries. Locations with high burdens of these disease categories and high prevailing temperatures showed strong heat impacts. Areas with moderate or cold temperatures exhibited protective effects on external causes but were generally associated with a high burden of cardiorespiratory and metabolic disease. In the second part, we applied our derived exposure–response surfaces to all locations using a global gridded dataset of daily temperatures. This allowed us to estimate daily temperature effects in areas for which no daily mortality data were available. While these global applications incorporated limitations and uncertainties, our model is a first important step toward estimating the temperature-related burden in areas that are potentially very strongly impacted by temperature—especially heat—but have no available data.

We found that minimum mortality temperatures vary by cause and climate (or temperature zone). We consequently estimated location-specific and year-specific TMRELs that reflect both the exposure–response relationship in a specific climate and the composition of causes of death in that location and year. Several studies have shown varying exposure–response functions in different cities that could not solely be explained by climate.^4^, ^5^, ^9^ Generally, infrastructure, such as the prevalence of air conditioning or heating, and housing insulation are likely explanations.^22^, ^23^ Our study highlights the importance of the composition of causes of death as another driver of this observed variation. Areas with a high burden of external causes of death exhibit a high heat-attributable burden, whereas areas with a high burden of cardiorespiratory and metabolic disease generally exhibit increased cold and heat impacts.

In a paper by Gasparrini and colleagues,^4^ the authors calculated all-cause PAFs for 13 countries, based on data from major cities. Three of these countries, the USA, Brazil, and China, were included in our study. Generally, cold PAFs in our study are lower than those derived by Gasparrini and colleagues, with 3·44% (95% UI 3·03–3·75) versus 5·51% (5·17–5·82) for the USA, 0·81% (0·70–0·93) versus 2·83% (2·34–3·30) for Brazil, and 4·28% (3·88–4·66) versus 10·36% (8·72–11·77) for China. In addition to differences in seasonality adjustment and lag periods, there are two other reasons that might contribute to these lower cold estimates: first, the study by Gasparrini and colleagues^4^ relies on point data from individual cities, whereas this study relies on data with continuous spatial coverage, including rural populations. Second, our study assesses cause-specific attributable burden, and we only included causes that we found to be statistically significantly associated with temperature. There are likely to be additional causes that did not meet our inclusion criteria despite being associated with temperature; and temperature-attributable burden for these causes would not be included in our estimates but would be captured in analyses of all-cause mortality like those by Gasparrini and colleagues.^4^ Despite these differences, the estimated heat PAFs for the three countries included in both studies are quite similar, with 0·33% (0·25–0·44) versus 0·35% (0·30–0·39) for the USA, 0·42% (0·33–0·51) versus 0·70% (0·45–0·93) for Brazil, and 0·43% (0·33–0·55) versus 0·64% (0·47–0·79) for China. Heat effects occur on a short-term time scale, and our methodological approach, which assumes only same-day impacts, is likely to capture the same heat effects as Gasparrini and colleagues.^4^ Differences in heat PAFs between the two studies are mostly observed for European countries. Gasparrini and colleagues^4^ observed consistently higher PAFs than our study, with 1·06% (0·96–1·16) versus 0·12% (0·08–0·18) for Spain and 1·62% (1·24–1·98) versus 0·13% (0·07–0·22) for Italy. Several studies have highlighted strong heat effects in southern European countries,^7^, ^8^ which might be the result of effect modifiers such as high urban density and low prevalence of air conditioning. Given data privacy restrictions in Europe, we were not able to include individual cause-specific data in this study and are likely to underestimate heat impacts in this region.

Our method for estimating RRs has some important limitations and differences from previous studies. First, while previous studies of temperature-mortality relationships have used distributed non-linear lag models to capture lagged temperature effects,^4^, ^5^, ^6^ we defined temperature effects as the short-term effect occurring on the same day of exposure. Given the number of locations and causes included in our analysis, our approach is more computationally tractable and facilitates generalisability but comes with the limitation that our estimates might be conservative and underestimate temperature-attributable burden. As previous studies have found stronger lagged effects for low temperatures, the degree of underestimation is likely to be greater for cold than for heat.^24^, ^25^, ^26^, ^27^ Second, while most previous studies have adjusted their temperature estimates for season, we did not include this adjustment because the strong correlation between temperature and seasonality challenges complete disentangling of these two factors;^28^ where season and temperature are strongly correlated, the inclusion of these colinear variables will inevitably dilute any association between temperature and mortality. Our method assumes that seasonal patterns observed in mortality are driven primarily by the direct and indirect effects of temperature, and we therefore ascribe deviations in daily mortality to temperature without making any attributions to seasonality. Third, because we only included causes of death that met specific inclusion criteria, our analysis does not include all causes of death that are likely to be sensitive to temperature, such as malaria and other infectious diseases. While this does not affect our burden estimates for the causes that we did include, the exclusion of burden from some causes means that we will underestimate the total burden attributable to heat and cold. Fourth, our analysis does not include non-fatal health outcomes. Data limitations precluded our inclusion of morbidity effects, but we hope to extend our model to include non-fatal outcomes in future iterations.

There are also important limitations to our method of applying RRs developed using data from nine countries to produce global estimates. First, while we accounted for heterogeneity between temperature zones, the paucity of relevant globally comprehensive data prevented us from explicitly accounting for infrastructure, behaviour, or other population-specific characteristics that have been shown to underlie heterogeneity of temperature-mortality relationships within similar climates.^22^, ^23^ Consequently, we might have underestimated temperature-attributable burden in locations with greater temperature sensitivity, and overestimated temperature-attributable burden in locations with less temperature sensitivity than the locations for which we had mortality data. Second, our mortality data were limited to nine countries and did not include data from Europe or south or southeast Asia, and include only one country in sub-Saharan Africa. Though some countries in southeast Asia have high-quality vital registration, these data were not publicly available with day of death, location, and ICD codes. Because our RR functions are derived from locations that include 29% of the world's population, span temperature zones inhabited by approximately 95% of the global population (as indicated by the mean annual temperature), and cover 79% of sociodemographic conditions (as indicated by the Socio-demographic Index) the uncertainty intervals that we estimate likely encompass the range of effect modification experienced by most of the world's population. Moreover, between GBD 2019^21^ and this iteration, we added vital registration data from South Africa, which provided a means of assessing the validity of our estimates for locations without data. In this study, we estimated that 8372 deaths (95% UI 7390–9399) were attributable to cold and 453 deaths (275–671) were attributable to heat in South Africa in 2019 (table). Reassuringly, the estimates are similar to those estimated for GBD 2019 for which 11 000 deaths (9200–12 610) attributable to cold and 300 deaths (155–623) attributable to heat were estimated for South Africa in the same year.^21^ For many countries, especially in low-latitude regions with high ambient temperatures, daily mortality data are not and will not be available for the foreseeable future, and our estimates provide an initial approximation of temperature-related mortality in these areas. Recently, Vicedo-Cabrera and colleagues^29^ quantified heat-related mortality attributable to climate change by linking exposure–response curves for all-cause mortality with modelled temperature due to anthropogenic emissions. Across all countries included in their study, 37·0% (range 20·5–76·3) of warm-season heat-related deaths were attributable to anthropogenic climate change. These findings underline the need for continued focus on temperature effects on health as increased adverse impacts of heat under global warming are expected, especially in areas that are already vulnerable. As additional data become available, estimates of the burden of disease attributable to non-optimal temperature exposure will be iteratively updated and expanded within the cycle of GBD releases. Despite limitations, the results presented here provide valuable information for public health policy by highlighting the relevance of temperature as a risk factor, as compared with other risk factors. These estimates thus provide vital information in support of efforts to address vulnerability that might then focus on reducing the underlying burden of disease (eg, cardiorespiratory and metabolic disease or external causes), or to develop strategies to reduce exposure (eg, spatial or urban planning, housing insulation, air conditioning) or behavioural education.

## Data sharing

To download the data used in these analyses, visit the Global Health Data Exchange GBD 2019 website. All code used in the analysis can be found online at https://github.com/ihmeuw/environmental_risk_factors/tree/temperature_lancet_2021.

## Declaration of interests

We declare no competing interests.
