
JOURNAL INFORMATION
==============================
NLM Title Abbreviation: Lancet
Journal ID: Lancet
NLM Journal ID: 2985213R

Lancet (London, England)

ISSN: 0140-6736
EISSN: 1474-547X

ARTICLE INFORMATION
==============================
PMCID: PMC8444083
PMID: 34509231
DOI: 10.1016/S0140-6736(21)01996-6
Article ID: S0140-6736(21)01996-6
Article version: 1
Subjects: Department of Error

Department of Error

Print publication date: 2021 Sep 11
Volume: 398
Issue: 10304
First page: 956
Last page: 956
Copyright: © 2021 The Author(s). Published by Elsevier Ltd.
Copyright year: 2021
Copyright holder: Elsevier Ltd
License: This is an open access article under the CC BY license (http://creativecommons.org/licenses/by/4.0/).
License URL: https://creativecommons.org/licenses/by/4.0/
Erratum for: Estimating the cause-specific relative risks of non-optimal temperature on daily mortality: a two-part modelling approach applied to the Global Burden of Disease Study 398 10301 21 8 2021 685 697 Lancet (London, England) 10.1016/S0140-6736(21)01700-1 PMC8387975 34419204

==============================
Burkart KG, Brauer M, Aravkin AY, et al. Estimating the cause-specific relative risks of non-optimal temperature on daily mortality: a two-part modelling approach applied to the Global Burden of Disease Study. Lancet 2021; 398: 685–97—In this Article, the spelling of author Jiawei He's name was incorrect. This correction has been made to the online version as of Sept 9, 2021.

