# Supplementary material for: Estimating the cause-specific relative risks of non-optimal temperature on daily mortality: a two-part modelling approach applied to the Global Burden of Disease Study
Source: Lancet. 2021 Aug 21;398(10301):685–97. doi: 10.1016/S0140-6736(21)01700-1 (PMC8387975; doi:10.1016/S0140-6736(21)01700-1)
Supplement: Supplementary appendix [file mmc1.pdf]

THE LANCET

Supplementary appendix

This appendix formed part of the original submission and has been peer reviewed. We post it as supplied by the authors.

Supplement to: Burkart KG, Brauer M, Aravkin AY, et al. Estimating the cause-specific relative risks of non-optimal temperature on daily mortality: a two-part modelling approach applied to the Global Burden of Disease Study. *Lancet* 2021; **398:** 685–97.

Appendix: supplementary methods and results for "Estimating the cause-specific relative risks of non-optimal temperature on daily mortality: a two-part modelling approach applied to the Global Burden of Disease Study"

This appendix provides further methodological details—including data source citations—and supplementary results for "Estimating the cause-specific relative risks of non-optimal temperature on daily mortality: a two-part modelling approach applied to the Global Burden of Disease Study".

Table of Contents

Section 1 List of abbreviations 6

Section 2 Guidelines for Accurate and Transparent Health Estimates Reporting (GATHER) compliance 6

Table S1: GATHER checklist 7

Section 3 Methods and data 8

Section 3.1 Exposure data 8

Figure S1: Global distribution of mean annual temperature categories (ie, climate zones) used for analysis (panel a) and density distribution of daily temperatures in each mean climate zone (panel b). 9

Figure S2: Time series of daily population-weighted temperatures in Brazil, Chile, China, Colombia Guatemala, Mexico, New Zealand, and the United States of America 10

Section 3.2 Mortality data 11

Table S2: Overview over mortality data collected from vital registration systems 11

Table S3: Total death counts for analysed causes of death by country 12

Section 3.3 Part 1: Modelling the relationship between daily temperature and cause-specific mortality and estimating the attributable burden of disease 14

Section 3.3.1 Data 14

Section 3.3.2 Relative risk modelling 15

Section 3.3.3 Cause selection 15

Section 3.3.4 Applying risk curves to estimates of the burden of disease 15

Section 3.4 Part 2: Global estimations of the temperature-attributable burden of disease 17

Table S4: Average risk-outcome scores across climate zones for individual causes. Causes with an average score above 0 were included into GBD. 17

Figure S3: Joint exposure-response curves displaying the relationship between daily mean temperature and the log RR for mortality from 17 selected causes 18

Section 3.4.1 Theoretical minimum risk exposure level 18

Figure S4: Global distribution of theoretical minimum risk exposure level (TMREL) in 1990 and 2019 18

Figure S5: GBD super-regions 19

Figure S6: Isopleth diagrams depicting the risk surface of the log RR of mortality of selected causes along daily and annual mean temperature. 20

Figure S7: Exposure-response curves displaying the relationship between daily mean temperature and the log RR for mortality from lower respiratory infections. 22

Figure S8: Exposure-response curves displaying the relationship between daily mean temperature and the log RR for mortality from ischaemic heart disease (IHD). 22

Figure S9: Exposure-response curves displaying the relationship between daily mean temperature and the log RR for mortality from stroke. 23

Figure S10: Exposure-response curves displaying the relationship between daily mean temperature and the log RR for mortality from hypertensive heart disease. 23

Figure S11: Exposure-response curves displaying the relationship between daily mean temperature and the log RR for mortality from cardiomyopathy and myocarditis. 24

Figure S12: Exposure-response curves displaying the relationship between daily mean temperature and the log RR for mortality from chronic obstructive pulmonary disease. 24

Figure S13: Exposure-response curves displaying the relationship between daily mean temperature and the log RR for mortality from diabetes mellitus. 25

Figure S14: Exposure-response curves displaying the relationship between daily mean temperature and the log RR for mortality from chronic kidney disease. 25

Figure S15: Exposure-response curves displaying the relationship between daily mean temperature and the log RR for mortality from road injuries. 26

Figure S16: Exposure-response curves displaying the relationship between daily mean temperature and the log RR for mortality from other transport-related injuries. 26

Figure S17: Exposure-response curves displaying the relationship between daily mean temperature and the log RR for mortality from drowning. 27

Figure S18: Exposure-response curves displaying the relationship between daily mean temperature and the log RR for mortality from mechanical injuries (exposure to mechanical forces). 27

Figure S19: Exposure-response curves displaying the relationship between daily mean temperature and the log RR for mortality from animal-related injuries (animal contact). 28

Figure S20: Exposure-response curves displaying the relationship between daily mean temperature and the log RR for mortality from disaster-related injuries (exposure to forces of nature). 28

Figure S21: Exposure-response curves displaying the relationship between daily mean temperature and the log RR for mortality from other unintentional injuries. 29

Figure S22: Exposure-response curves displaying the relationship between daily mean temperature and the log RR for mortality from suicide (self-harm). 29

Figure S23: Exposure-response curves displaying the relationship between daily mean temperature and the log RR for mortality from homicide (interpersonal violence). 30

Figure S24: Intra-country differences in the composition of deaths and DALYs attributable to high and low temperatures Sao Paulo (Brazil), Piaui (Brazil), Michigan (USA), and Arizona (USA), Mexico City (MEX), and Campeche (MEX) in 2019. 30

Table S5: Burden estimates for high temperature, low temperature and non-optimal temperature exposure by super-region in 1990 and 2019 (95%UI displayed in parentheses) 32

Table S6: Burden estimates for high temperature, low temperature and non-optimal temperature exposure by cause in 1990 and 2019 (95%UI displayed in parentheses) 33

Figure S25: Spatial distribution of the proportion of deaths due to lower respiratory infections attributable to high temperature (A), low temperature (B), and non-optimal temperature (C) exposure, 2019 35

Figure S26: Spatial distribution of the proportion of deaths due to ischaemic heart disease attributable to high temperature (A), low temperature (B), and non-optimal temperature (C) exposure, 2019 36

Figure S27: Spatial distribution of the proportion of deaths due to stroke attributable to high temperature (A), low temperature (B), and non-optimal temperature (C) exposure, 2019 37

Figure S28: Spatial distribution of the proportion of deaths due to hypertensive heart disease attributable to high temperature (A), low temperature (B), and non-optimal temperature (C) exposure, 2019 38

Figure S29: Spatial distribution of the proportion of deaths due to cardiomyopathy and myocarditis attributable to high temperature (A), low temperature (B), and non-optimal temperature (C) exposure, 2019 39

Figure S30: Spatial distribution of the proportion of deaths due to chronic obstructive pulmonary disease attributable to high temperature (A), low temperature (B), and non-optimal temperature (C) exposure, 2019 40

Figure S31: Spatial distribution of the proportion of deaths due to diabetes mellitus attributable to high temperature (A), low temperature (B), and non-optimal temperature (C) exposure, 2019 41

Figure S32: Spatial distribution of the proportion of deaths due to chronic kidney disease attributable to high temperature (A), low temperature (B), and non-optimal temperature (C) exposure, 2019 42

Figure S33: Spatial distribution of the proportion of deaths due to road injuries attributable to high temperature (A), low temperature (B), and non-optimal temperature (C) exposure, 2019 43

Figure S34: Spatial distribution of the proportion of deaths due to other transport-related injuries attributable to high temperature (A), low temperature (B), and non-optimal temperature (C) exposure, 2019 44

Figure S35: Spatial distribution of the proportion of deaths due to drowning attributable to high temperature (A), low temperature (B), and non-optimal temperature (C) exposure, 2019 45

Figure S36: Spatial distribution of the proportion of deaths due to mechanical injuries (exposure to mechanical forces) attributable to high temperature (A), low temperature (B), and non-optimal temperature (C) exposure, 2019 46

Figure S37: Spatial distribution of the proportion of deaths due to animal-related injuries (animal contact) attributable to high temperature (A), low temperature (B), and non-optimal temperature (C) exposure, 2019 47

Figure S38: Spatial distribution of the proportion of deaths due to disaster-related injuries (exposure to forces of nature) attributable to high temperature (A), low temperature (B), and non-optimal temperature (C) exposure, 2019 48

Figure S39: Spatial distribution of the proportion of deaths due to other unintentional injuries attributable to high temperature (A), low temperature (B), and non-optimal temperature (C) exposure, 2019 49

Figure S40: Spatial distribution of the proportion of deaths due to suicide (self-harm) attributable to high temperature (A), low temperature (B), and non-optimal temperature (C) exposure, 2019 50

Figure S41: Spatial distribution of the proportion of deaths due to homicide (interpersonal violence) attributable to high temperature (A), low temperature (B), and non-optimal temperature (C) exposure, 2019 51

Figure S42: Spatial distribution of DALYs (per 100,000) due to lower respiratory infections attributable to high temperature (A), low temperature (B), and non-optimal temperature (C) exposure, 2019 52

Figure S43: Spatial distribution of DALYs (per 100,000) due to ischaemic heart disease attributable to high temperature (A), low temperature (B), and non-optimal temperature (C) exposure, 2019 53

Figure S44: Spatial distribution of DALYs (per 100,000) due to stroke attributable to high temperature (A), low temperature (B), and non-optimal temperature (C) exposure, 2019 54

Figure S45: Spatial distribution of DALYs (per 100,000) due to hypertensive heart disease attributable to high temperature (A), low temperature (B), and non-optimal temperature (C) exposure, 2019 55

Figure S46: Spatial distribution of DALYs (per 100,000) due to cardiomyopathy and myocarditis attributable to high temperature (A), low temperature (B), and non-optimal temperature (C) exposure, 2019 56

Figure S47: Spatial distribution of DALYs (per 100,000) due to chronic obstructive pulmonary disease attributable to high temperature (A), low temperature (B), and non-optimal temperature (C) exposure, 2019 57

Figure S48: Spatial distribution of DALYs (per 100,000) due to diabetes mellitus attributable to high temperature (A), low temperature (B), and non-optimal temperature (C) exposure, 2019 58

Figure S49: Spatial distribution of DALYs (per 100,000) due to chronic kidney disease attributable to high temperature (A), low temperature (B), and non-optimal temperature (C) exposure, 2019 59

Figure S50: Spatial distribution of DALYs (per 100,000) due to road injuries attributable to high temperature (A), low temperature (B), and non-optimal temperature (C) exposure, 2019 60

Figure S51: Spatial distribution of DALYs (per 100,000) due to other transport-related injuries attributable to high temperature (A), low temperature (B), and non-optimal temperature (C) exposure, 2019 61

Figure S52: Spatial distribution of DALYs (per 100,000) due to drowning attributable to high temperature (A), low temperature (B), and non-optimal temperature (C) exposure, 2019 62
Figure S53: Spatial distribution of DALYs (per 100,000) due to mechanical injuries (exposure to mechanical forces) attributable to high temperature (A), low temperature (B), and non-optimal temperature (C) exposure, 2019 63
Figure S54: Spatial distribution of DALYs (per 100,000) due to animal-related injuries (animal contact) attributable to high temperature (A), low temperature (B), and non-optimal temperature (C) exposure, 2019 64
Figure S55: Spatial distribution of DALYs (per 100,000) due to disaster-related injuries (exposure to forces of nature) attributable to high temperature (A), low temperature (B), and non-optimal temperature (C) exposure, 2019 65
Figure S56: Spatial distribution of DALYs (per 100,000) due to other unintentional injuries attributable to high temperature (A), low temperature (B), and non-optimal temperature (C) exposure, 2019 66
Figure S57: Spatial distribution of DALYs (per 100,000) due to suicide (self-harm) attributable to high temperature (A), low temperature (B), and non-optimal temperature (C) exposure, 2019 67
Figure S58: Spatial distribution of DALYs (per 100,000) due to homicide (interpersonal violence) attributable to high temperature (A), low temperature (B), and non-optimal temperature (C) exposure, 2019 68
Section 4 Drivers of temperature-induced morbidity and mortality 69
Section 4.1 Cardiovascular disease 69
Section 4.1.1 Ischaemic heart disease 69
Section 4.1.2 Stroke 69
Section 4.2 Respiratory diseases 70
Section 4.2.1 Upper and lower respiratory infection 70
Section 4.2.2 Chronic obstructive pulmonary disease (COPD) 70
Section 4.3 Chronic kidney disease (CKD) 70
Section 4.4 Diabetes 70
Section 5 Data source citations 71
Table S7. Vital registration data source citations by location and year 71
Section 6 References 76

Section 1 List of abbreviations

**Abbreviation**	**Full phrase**
CKD	chronic kidney disease
CRA	comparative risk assessment
COPD	chronic obstructive pulmonary disease
CSMR	cause-specific mortality rate
DALY	disability-adjusted life-year
ECMWF	European Centre for Medium-Range Weather Forecasts
ERA5	ECMWF Reanalysis v5
GATHER	Guidelines for Accurate and Transparent Health Estimates Reporting
GBD	Global Burden of Diseases, Injuries, and Risk Factors Study
GHDx	Global Health Data Exchange website
HDL	high density lipoprotein
HS	haemorrhage stroke
ICD	International Classification of Diseases
IHD	ischaemic heart disease
IS	ischaemic stroke
LRI	lower respiratory infections
MR-BRT	meta-regression—Bayesian, regularised, trimmed
PAF	population attributable fraction
ROS	risk-outcome score
RR	relative risk
SEV	summary exposure value
TMREL	theoretical minimum risk exposure level
UI	uncertainty interval
YLDs	years lived with disability
YLLs	years of life lost

Section 2 Guidelines for Accurate and Transparent Health Estimates Reporting (GATHER) compliance

This study complies with GATHER recommendations.[1] We have documented the steps in our analytical procedures and detailed the data sources used. See table S1 for the GATHER checklist. The GATHER recommendations can be found on the GATHER website.

Table S1: GATHER checklist

Item #	Checklist item	Location
**Objectives and funding**		
1	Define the indicator(s), populations (including age, sex, and geographic entities), and time period(s) for which estimates were made.	Main text: Introduction
2	List the funding sources for the work.	Main text: Acknowledgments
**Data Inputs**		
For all data inputs from multiple sources that are synthesised as part of the study:		
3	Describe how the data were identified and how the data were accessed.	Main text: Methodology<br>Appendix: Section 3
4	Specify the inclusion and exclusion criteria. Identify all ad-hoc exclusions.	Main text: Methodology
5	Provide information on all included data sources and their main characteristics. For each data source used, report reference information or contact name/institution, population represented, data collection method, year(s) of data collection, sex and age range, diagnostic criteria or measurement method, and sample size, as relevant.	Main text: Methodology<br>Appendix: Section 3
6	Identify and describe any categories of input data that have potentially important biases (e.g., based on characteristics listed in item 5).	Main text: Methodology<br>Appendix: Section 3
For data inputs that contribute to the analysis but were not synthesised as part of the study:		
7	Describe and give sources for any other data inputs.	N/A
For all data inputs:		
8	Provide all data inputs in a file format from which data can be efficiently extracted (e.g., a spreadsheet rather than a PDF), including all relevant meta-data listed in item 5. For any data inputs that cannot be shared because of ethical or legal reasons, such as third-party ownership, provide a contact name or the name of the institution that retains the right to the data.	Data and citations available through: *http://ghdx.healthdata.org/gbd-2019/data-input-sources*
**Data analysis**		
9	Provide a conceptual overview of the data analysis method. A diagram may be helpful.	Main text: Methodology<br>Appendix: Section 3
10	Provide a detailed description of all steps of the analysis, including mathematical formulae. This description should cover, as relevant, data cleaning, data pre-processing, data adjustments and weighting of data sources, and mathematical or statistical model(s).	Main text: Methodology<br>Appendix: Section 3
11	Describe how candidate models were evaluated and how the final model(s) were selected.	Appendix: Section 3
12	Provide the results of an evaluation of model performance, if done, as well as the results of any relevant sensitivity analysis.	N/A
13	Describe methods for calculating uncertainty of the estimates. State which sources of uncertainty were, and were not, accounted for in the uncertainty analysis.	Main text: Methodology<br>Appendix: Section 3
14	State how analytic or statistical source code used to generate estimates can be accessed.	Available online through: *https://github.com/ihmeuw/environmental_risk_factors/tree/temperature_lancet_2021*
**Results and Discussion**		
15	Provide published estimates in a file format from which data can be efficiently extracted.	Available online through: *https://github.com/ihmeuw/environmental_risk_factors/tree/temperature_lancet_2021*
16	Report a quantitative measure of the uncertainty of the estimates (e.g. uncertainty intervals).	All estimates are reported with 95% uncertainty intervals.

17	Interpret results in light of existing evidence. If updating a previous set of estimates, describe the reasons for changes in estimates.	Main text: Discussion
18	Discuss limitations of the estimates. Include a discussion of any modelling assumptions or data limitations that affect interpretation of the estimates.	Main text: Discussion

Section 3 Methods and data

Section 3.1 Exposure data

For each pixel, we calculated two temperature metrics: First, we calculated daily mean temperature as the mean of all 24-hour temperature estimates for that pixel and day, rounded to the nearest 1°C. Uncertainty estimates for hourly data was provided for every 3-hours and for a 0.5° x 0.5° resolution. We calculated daily averages for uncertainty and mapped these to the higher resolution temperature values. Second, we calculated the temperature zone as the mean temperature in the pixel across all days between 1980 and 2016 and categorised daily mean temperature by truncating all values below the first and above the 99th percentiles, and rounded all values to the nearest 1°C.

Figure S1: Global distribution of mean annual temperature categories (ie, climate zones) used for analysis (panel a) and density distribution of daily temperatures in each mean climate zone (panel b).

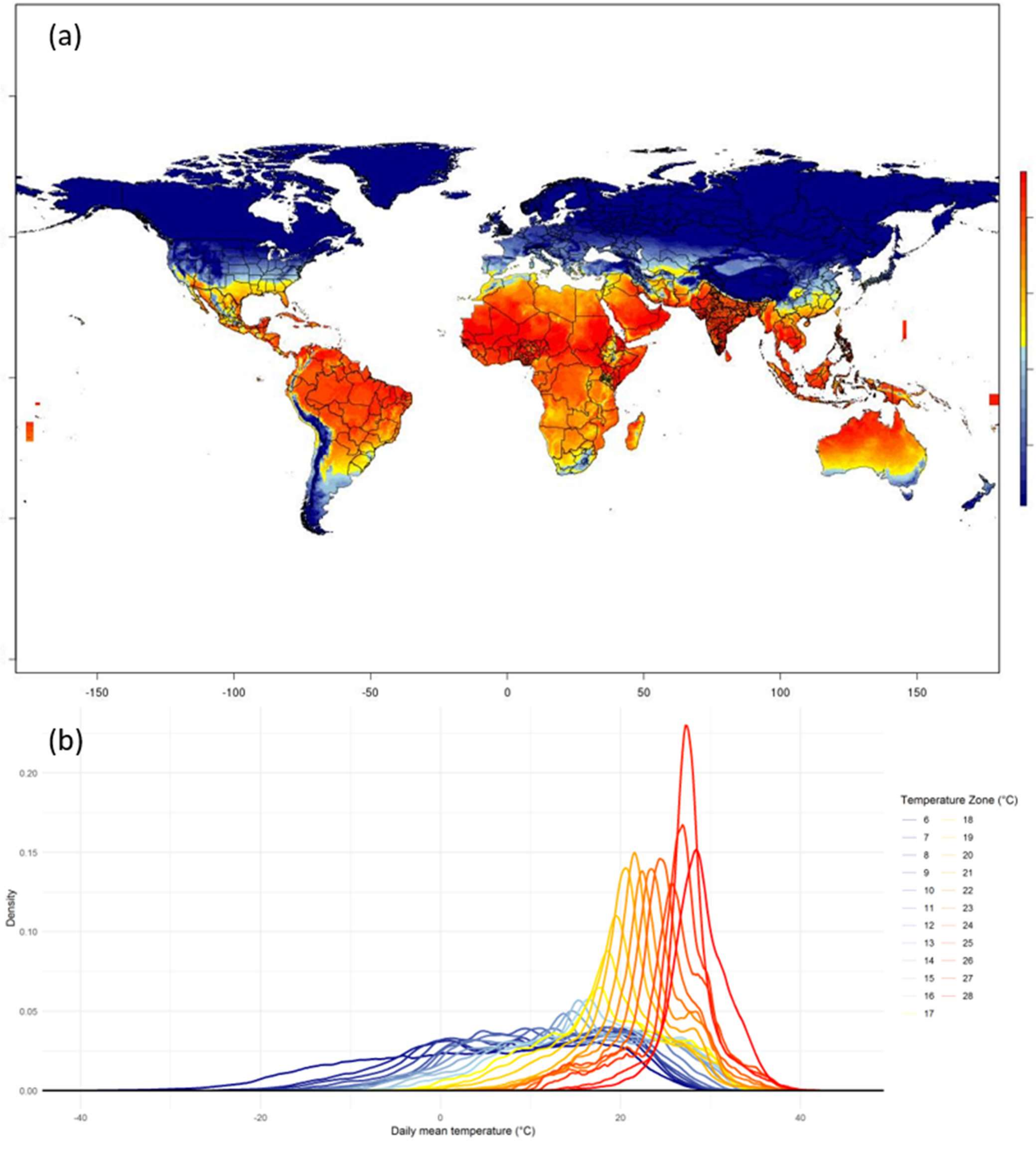

Figure S2: Time series of daily population-weighted temperatures in Brazil, Chile, China, Colombia Guatemala, Mexico, New Zealand, and the United States of America

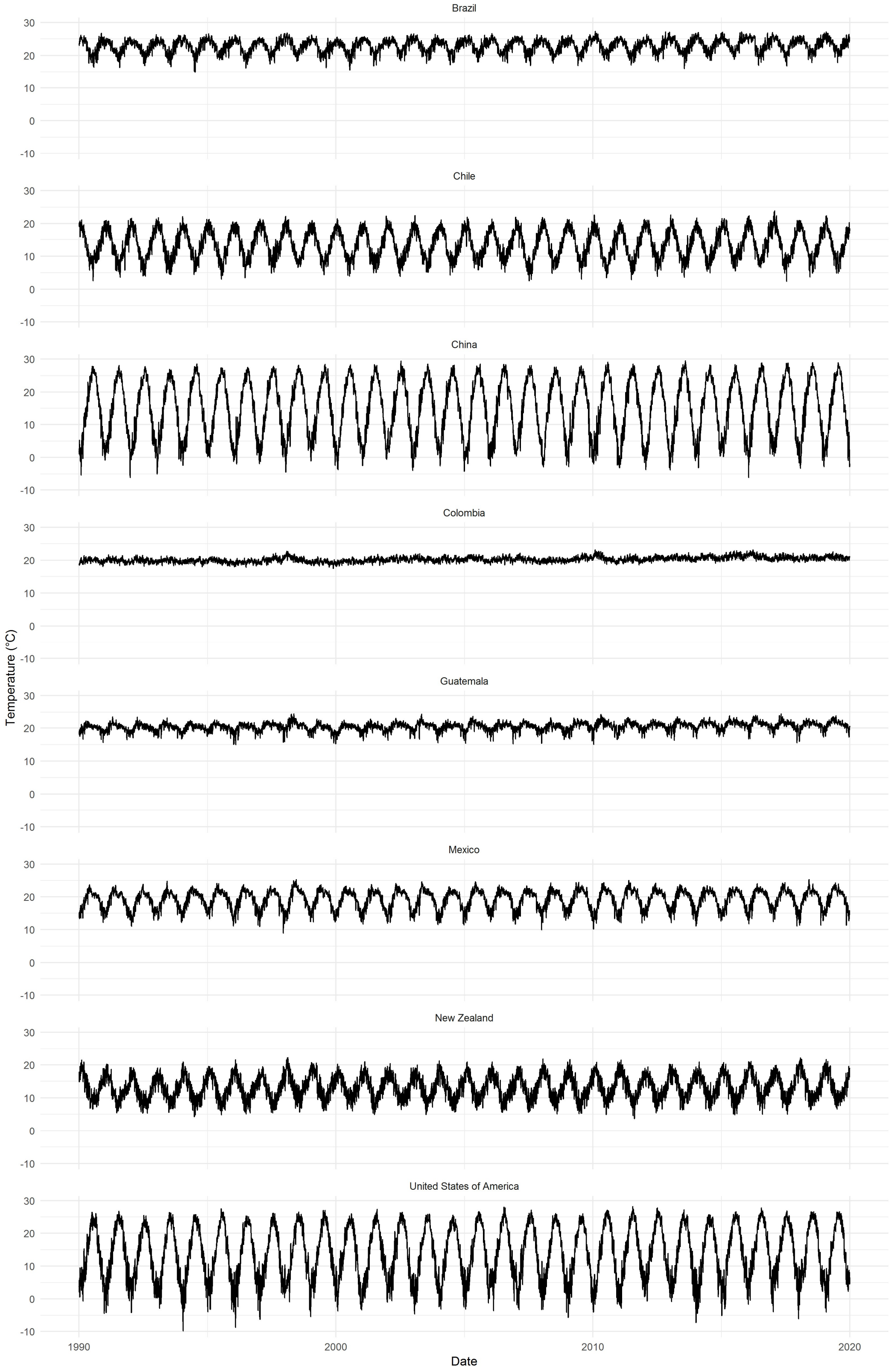

Section 3.2 Mortality data

Table S2: Overview over mortality data collected from vital registration systems

Country	Years	Admin units	Deaths
Brazil	1999 – 2016	Municipality (n=5,570)	19.9 million
Chile	1990 – 1996<br>2009 – 2011	Regions (n = 15)	0.82 million
China	2015 - 2016	County (n=2,556)	12.2 million
Colombia	2001 – 2005	Municipality (n=1,125)	0.95 million
Guatemala	2009 – 2016	Municipality (n=333)	0.49 million
Mexico	1996 – 2015	Municipality (n=2,438)	9.88 million
New Zealand	1988 – 2014	District Health Board (n=20)	0.76 million
South Africa	1997 – 2016	Province (n=1)	1.80 million
United States	1980 - 1988	County (n=3,140)	18.1 million
Total	1980 – 2016	n = 15,197	64.9 million

Table S3: Total death counts for analysed causes of death by country

The absence of counts for a given cause and location does not imply that no such deaths occurred, but that nuances of a given dataset precluded mapping deaths to a given cause. Most often this occurred where a given dataset did not include the external cause for deaths from injury causes (ie, injury codes may be for either the nature of the injury (eg, crushing injury of skull) or its external cause (eg, road injury)).

Cause	Brazil	Chile	China	Colombia	Guatemala	Mexico	New Zealand	United States	South Africa	Total
Lower respiratory infections	1,119,646	3,593	236,466	34,658	55,837	210,884	12,166	36,220	180,716	1,890,186
Ischaemic heart disease	2,734,466	76,930	2,308,685	144,614	51,504	1,236,766	171,843	2,268,912	124,524	9,118,244
Stroke	1,938,334	29,783	2,594,745	72,568	25,543	418,639	48,613	306,484	88,555	5,523,262
Hypertensive heart disease	342,487	11,621	416,538	22,015	4,443	112,218	4,295	91,814	29,127	1,034,558
Cardiomyopathy and myocarditis	260,790	583	30,525	5,217	942	11,884	3,886	387,538	17,579	718,944
Chronic obstructive pulmonary disease	999,884	19,866	1,102,349	50,434	8,475	397,773	41,772	263,460	62,624	2,946,637
Diabetes mellitus	740,414	3,611	217,465	34,938	31,349	639,670	11,865	151,700	73,201	1,904,214
Chronic kidney disease	431,817	12,705	222,572	22,320	29,832	654,484	12,738	62,082	38,356	1,486,906
Road injuries	762,256	-	359,088	42,600	6,860	234,203	6,181	-	58,002	1,469,190
Other transport injuries	31,118	-	10,100	2,416	982	11,099	1,659	143	7,676	65,193
Drowning	126,657	-	64,132	6,569	2,334	44,458	1,615	-	10,798	256,563
Exposure to mechanical forces	41,735	-	41,975	3,477	6,696	21,192	1,155	4,504	28,842	149,577
Animal contact	9,054	-	2,879	907	183	5,888	82	-	1,672	20,664
Exposure to forces of nature	4,558	-	843	875	177	2,975	234	870	355	10,887

Other unintentional injuries	52,888	-	21,893	2,087	1,054	12,155	288	147	3,348	93,859
Self-harm	212,637	-	129,935	14,048	4,638	91,449	13,893	4,158	49,665	520,422
Interpersonal violence	1,043,798	-	13,016	135,252	33,768	267,555	1,644	22,051	44,788	1,561,872

Section 3.3 Part 1: Modelling the relationship between daily temperature and cause-specific mortality and estimating the attributable burden of disease

Section 3.3.1 Data

We used temperature estimates from the ERA5, a gridded reanalysis dataset produced by the European Centre for Medium Range Weather Forecasts (ECMWF) with 0·25° x 0·25° spatial and sub-daily temporal resolutions.[2] The ERA5 is a latest-generation reanalysis dataset and, to our knowledge, it offers the highest temporal and spatial resolution among reanalysis datasets that offer both global coverage and a sufficiently long time series, currently from 1979 to present. The ERA5 dataset also provides uncertainty for estimates on a 0·5° x 0·5° spatial and three-hourly resolution, reflecting lower confidence in areas with fewer measurement stations, such as Africa or South Asia. Using temperature estimates for the period from 1 January 1980 through 31 December 2019, for each pixel we calculated the daily mean temperature, for each day, and the mean temperature across all days, hereafter referred to as the *temperature zone.*

We derived gridded population data from the WorldPop project.[3] This data source provides multi-temporal, globally consistent, high-resolution (1 km) human population data for the years 2000, 2005, 2010, 2015, and 2020. To produce annual population estimates, we interpolated between the five-year bins and extrapolated by using the 2000–2005 growth rate for back-casting until 1990.

Our analysis utilised daily cause-specific mortality data from nine countries: Brazil, Chile, China, Colombia, Guatemala, Mexico, New Zealand, South Africa, and the USA. These are all the countries where individual deaths with information on ICD code, administrative unit, and day were available based on publicly available datasets or through GBD collaborators. These data represent a range of socio-demographic conditions and span the distribution of global temperatures, with only 5% of the global population residing in zones outside of the temperature range (mean temperature zones from 6° to 28°C) represented in these data. The data were collected at the administrative level of municipalities or counties. We mapped ICD codes to GBD causes; where deaths were coded to “garbage codes,” we applied individual-level garbage-code redistribution based on GBD 2019 cause of death redistribution methods.[4] When evaluating cause-specific mortality as in our analysis, and especially when including data from multiple countries, it is important to harmonise the data to account and adjust for between-location variation in coding practices. The general methodology for the GBD cause redistribution is discussed in detail elsewhere.[5] Table S2 (section 3.2) gives a detailed description of the mortality data. Additionally, we provide further meta-information regarding vital registration data in table S7 (section 5).

For each death, we ascertained the temperature zone of location of the death, and the daily mean temperature for the day and location in which the death occurred. To avoid fitting to overly influential and noisy datapoints at extreme temperatures with few data, and to avoid extrapolations beyond the range of temperatures that correspond to the locations in our mortality data, we truncated temperature zones to the 1st and 99th percentiles of those in our mortality dataset; and we truncated daily mean temperatures to the 1st and 99th percentiles of daily temperatures within each temperature zone. We then calculated cause-specific mortality rates (CSMR) for each GBD Level 3 cause of death, c, and for each combination of administrative Level 2 location (eg, county or municipality), l_2, and daily mean temperature, t:

$$CSMR_{cl_2t} = \frac{d_{cl_2t}}{pop_{l_2} \cdot days_{l_2t}}$$

where d is the number of deaths, *pop* is the population, and *days* are the number of days of observation. We calculated the reference mortality rate for each cause and administrative Level 2 location as the mean CSMR for that cause in that location across all daily mean temperatures.

$$CSMR_{cl_2} = \frac{\sum_{t=1}^{n} d_{cl_2t}}{pop_{l_2} \cdot days_{l_2}}$$

Finally, we calculated relative risks (RR) by cause and administrative Level 2 location as the corresponding CSMR at a given daily mean temperature divided by the reference CSMR for that cause and location:

$$RR_{cl_2t} = \frac{CSMR_{cl_2t}}{CSMR_{cl_2}}$$

In converting CSMRs to RRs within each administrative Level 2 location, we were able to look at the association between temperature and mortality within a given location, rather than between locations, thereby removing any location effects that could have confounded our estimates. Since many administrative Level 2 locations have small populations, we commonly observed few or no deaths for a given cause, daily mean temperature, and location. To minimise problems with small numbers, including the inability to log-transform zero rates, we aggregated administrative Level 2 RRs by calculating the population-weighted mean RR for each

combination of administrative Level 1 location (eg, state, province), l_l, and temperature zone, z, and treated these aggregate RRs as our unit of analysis:

$$RR_{cl_1zt} = \sum_{l_2=1}^{n} RR_{cl_2t} \cdot {}^{pop_{l_2} \cdot days_{l_2t}} \Big/ {}_{pop_{l_1z} \cdot days_{l_1zt}}$$

Section 3.3.2 Relative risk modelling

To estimate outcomes based on daily mean temperature and temperature zone, we used a robust meta-regression framework, implemented through the MR-BRT tool (meta-regression—Bayesian, regularised, trimmed).[6–8] The tool allows three features that are essential to the analysis: First, a meta-analytic framework that can handle heterogeneous data sources. Modelling along different temperature zones and integrating data from all locations into one model allowed us to stabilise our estimates across zones. Second, a robust approach to outlier detection and removal using trimming methodology which is standard in a vast array of inference and machine learning problems.[9–11] and third, specification of the functional dependence of outcome versus daily mean temperature and temperature zone as a two-dimensional surface through a spline interface.[6] The functional relationship between any outcome *y* and input variables (*t1, t2*) models y as a linear combination of 2D spline basis elements. Each spline basis element is a product of individual basis elements for 1D splines for *t1* and *t2*. Therefore, the inference problem looks for a combination of simple curvilinear 2D elements that fit the data while preserving smoothness across element boundaries. The MR-BRT tool also allows prior information to influence the shape of the spline, particularly in areas with sparse data and prior knowledge about the exposure–response relationship. For exposure–response relationships that depicted a J- or U-shape, we fixed the curve to monotonically decrease or increase beyond the threshold temperature. For exposure–response relationships that displayed a one-directional relationship (ie, all external causes) we imposed monotonicity over the entire exposure range. In the direction of temperature zone, we used a cubic spline with two knots; and in the direction of daily mean temperature, we used a cubic spline with three knots for outcomes with J- or U-shape and a linear spline with two knots for monotonically increasing outcomes. To evaluate and select final models, we ran iterative models in MR-BRT with different numbers of knots and shape constraints. We evaluated models based on visual inspection of curves, choosing those that best balanced flexibility and stability. Figure 1 in the main text displays raw RR and modelled splines.

Section 3.3.3 Cause selection

Starting with a list of all Level 3 causes in the GBD cause hierarchy (N=176), we first reduced the set of potential causes by excluding those causes for which no deaths were recorded in our mortality dataset (N=44). We also excluded causes for which vital registration data are not used to model mortality due to known inconsistencies in classification practices across countries, including dementia and protein-energy malnutrition (N=2).[12] We estimated RR surfaces and uncertainties for all remaining (N=130) Level 3 causes in the GBD cause hierarchy. For each cause, we calculated a risk-outcome score using methods developed for evaluating risk-outcome pairs in the GBD study. Briefly, for each cause and each temperature zone we normalised the risk curve so that the RR was equal to 1·0 at the temperature associated with the lowest risk, and log-transformed the resulting normalised curves. We then estimated the area between the lower bound of the 95% UI and the null (ie, log-RR of 0·0). Regions of the risk curve for which the 95% UI includes the null will produce negative scores, and regions that exclude the null will produce positive scores; a score of zero indicates that the lower bound is equal to the null. With that, we included all causes for which the mean risk-outcome score exceeded zero. A table with average risk-outcome scores for individual causes is provided in table S4 (section 3.4).

Section 3.3.4 Applying risk curves to estimates of the burden of disease

Section 3.3.4.1 Comparative risk assessment framework

We estimated burden attributable to daily non-optimal temperature using the comparative risk assessment metric (CRA) framework developed by Murray and Lopez.[13] This approach uses four inputs: 1) the exposure levels for the risk factor, 2) the relative risk of the outcome as a function of exposure, 3) the counterfactual level of risk factor exposure, or theoretical minimum-risk exposure level (TMREL), and 4) the estimate of the burden being assessed for the cause (ie, number of deaths, years of life lost [YLLs], years lived with disability [YLDs], or disability-adjusted life-years [DALYs]).[13]

Section 3.3.4.2 TMREL estimation

To estimate risk-attributable burden, we established a counterfactual level of exposure that was associated with the lowest overall burden across all causes that were included. Specifically, the TMREL was the location-specific daily temperature associated with the lowest mortality rates for all included causes combined. As comprehensive cause of death estimates do not exist at a finer spatial resolution than GBD locations, we assumed that the cause composition of deaths was consistent across all pixels within a given GBD national or subnational location.

For a given temperature zone, *z*, daily temperature, *t*, GBD location, *l*, and year, *y*, we calculated the all-cause relative risk as the death-weighted mean of the cause-specific risk curves for all causes, *c*, that were included as outcomes of temperature exposure:

$$RR_{ztly} = \frac{\sum_{c=1}^{n} deaths_{cztly} \bullet RR_{cztly}}{deaths_{ztly}}$$

Then, for each temperature zone, location, and year, we calculated the TMREL as the value of daily temperature with the lowest RR:

$$TMREL_{zly} = \arg\min_{t}(RR_{tzly})$$

This approach results in location-specific TMRELs that vary as a function of each location's mean temperature and cause composition, and it represents the first use of spatially varying TMRELs in the GBD study.

Section 3.3.4.3 PAF calculation and burden of disease estimations

We calculated the PAF, ie, the proportion of cause-specific deaths attributable to high or low temperatures, for each pixel and day, based on the RR associated with a given daily mean temperature within a given temperature zone. We calculated PAFs for high and low temperatures above and below the TMREL. The non-optimal temperature PAF is an aggregate of the high and low temperature PAFs.

$$PAF_{cztd} = \frac{RR_{czt} - 1}{RR_{czt}}$$

Where *c* is the cause, *z* is the temperature zone, and *t* is the daily mean temperature for a given pixel and day *d*. We then calculated population-weighted averages for each day in location l and derived annual PAFs for each year *y* as the sum of daily PAFs.

$$PAF_{cld} = \sum_{p=1}^{n} {pop_{ply}}/{pop_{ly}} \bullet PAF_{cztd}$$

$$PAF_{cly} = \sum_{d=1}^{365/366} PAF_{cld}$$

We used estimates of cause-specific burden from GBD 2019[14] and estimated the temperature-attributable burden for each cause as the product of the total burden for that cause and the corresponding PAF for each GBD location, year, age group, and sex.

We propagated uncertainty from all components of the modelling chain using posterior simulation: for each quantity of interest, we pulled 1000 random draws from the posterior distribution of the estimate and performed all calculations at the draw level. For example, in estimating PAFs, we took 1000 draws from the posterior distribution of our estimates of RRs and 1000 draws from the distribution of temperature estimates, and for each of those 1000 draws calculated the resulting PAF to produce 1000 draws for our estimate of the PAF.

Section 3.3.4.4 Summary exposure values

The SEVs are measures of risk-weighted prevalence of exposure and range from 0% to 100%.[15] A SEV of 0% reflects no risk exposure, while 100% indicates that the entire population is exposed to the maximum possible level for that risk. For each cause and temperature zone, we estimated RR_{max} as the 99th percentile of RRs experienced for that cause and in that zone, across all years and locations, weighted by person-days of exposure. We then calculated the SEV for each cause, c, temperature zone, *z*, GBD location, *l*, and year, *y:*

$$SEV_{czly} = \frac{\sum_{t=t_{min_{zly}}}^{t_{max_{zly}}} {pop_{ztly}}/{pop_{zly}} \bullet (RR_{czt} - 1)}{RR_{max_{cz}}}$$

where *t* is daily mean temperature, and $t_{min_{zly}}$ and $t_{max_{zly}}$ are the minimum and maximum observed daily mean temperatures in location *l*, zone *z*, and year *y*. We then calculated the SEV for a given GBD location and year as the population-weighted average of

the zone-specific SEVs, and the all-cause SEVs as the simple means of all cause-specific SEVs; UIs were estimated based on 1000 draws of temperature, TMREL, and RR.

Section 3.4 Part 2: Global estimations of the temperature-attributable burden of disease

For generating global estimations, we applied the derived cause-specific exposure–response curves to a grid of global daily temperature exposure data from the ERA5 dataset. We calculated the PAF for each temperature pixel and population-weighted pixels before aggregating to the country level. The exposure–response curves were truncated at the outer ends of the temperature distribution observed in locations included in the mortality dataset. This means that the RR were kept constant for maximum and minimum mean annual and daily temperature. For instance, for areas with a mean annual temperature below 6°C or above 28°C, the exposure–response curves for 6°C and 28°C were applied. Similarly, if daily mean daily temperatures in the extrapolation areas were below or above the daily mean temperatures within the locations with mortality, the RR at the maximum range of the curve was applied. The calculation of PAFs, TMRELs, and the attributable burden of disease was carried out analogously to the estimations for within-sample locations as described above.

RRs were calculated in R and Python, PAFs and TMRELs were calculated in R and burden of disease was calculated in Python.

Table S4: Average risk-outcome scores across climate zones for individual causes. Causes with an average score above 0 were included into GBD.

Rank	Cause (level 3 [a])	Parent cause (level 2 [a])	Cause label (level 3 [a])	Score average
1	Drowning	Unintentional injuries	inj_drowning	0.361087
2	Other unintentional injuries	Unintentional injuries	inj_othunintent	0.269039
3	Lower respiratory infections	Respiratory infections and tuberculosis	lri	0.118761
4	Chronic obstructive pulmonary disease	Chronic respiratory diseases	resp_copd	0.105613
5	Animal-related injuries	Unintentional injuries	inj_animal	0.09697
6	Ischaemic heart disease	Cardiovascular diseases	cvd_ihd	0.08433
7	Stroke	Cardiovascular diseases	cvd_stroke	0.071678
8	Suicide	Self–harm and interpersonal violence	inj_suicide	0.070617
9	Disaster-related injuries	Unintentional injuries	inj_disaster	0.068935
10	Homicide	Self–harm and interpersonal violence	inj_homicide	0.068804
11	Mechanical injuries	Unintentional injuries	inj_mech	0.066174
12	Other transport-related injuries	Transport injuries	inj_trans_other	0.052465
13	Hypertensive heart disease	Cardiovascular diseases	cvd_htn	0.05067
14	Transport-related injuries	Transport injuries	inj_trans_road	0.048922
15	Diabetes	Diabetes and kidney diseases	diabetes	0.044704
16	Cardiomyopathy and myocarditis	Cardiovascular diseases	cvd_cmp	0.044378
17	Chronic kidney disease	Diabetes and kidney diseases	ckd	0.032174

[a] Within the GBD hierarchy, causes are grouped into four levels: Level 1 includes non-communicable diseases; injuries; and a summary category of infectious diseases, maternal and neonatal disorders, and nutritional deficiencies. Level 2, reflects a list of 22 disease and injury, such as respiratory infections and tuberculosis, cardiovascular diseases, and transport injuries. Level 3 includes specific causes such as tuberculosis, stroke, and road injuries. In some cases, these Level 3 causes are the most detailed classification, whereas for others a more detailed category is specified at Level 4. Examples of Level 4 causes include latent tuberculosis infection, ischaemic stroke, and pedestrian road injuries.

Figure S3: Joint exposure-response curves displaying the relationship between daily mean temperature and the log RR for mortality from 17 selected causes

The 17 selected causes include lower respiratory infections, ischaemic heart disease, stroke, hypertensive heart disease, cardiomyopathy and myocarditis, chronic obstructive pulmonary disease, diabetes, chronic kidney disease, road injuries, other transport injuries, drowning, exposure to mechanical forces, animal contact, exposure to forces of nature, other unintentional injuries, self-harm (suicide), and interpersonal violence (homicide). The RR is referenced to the theoretical minimum exposure risk level (TMREL) which represents the minimum mortality temperature for death-weighted multi-cause curves in each mean annual temperature category. The black solid line depicts the location of the TMREL in each mean annual temperature category with dashed lines depicting 95% UI of the TMREL

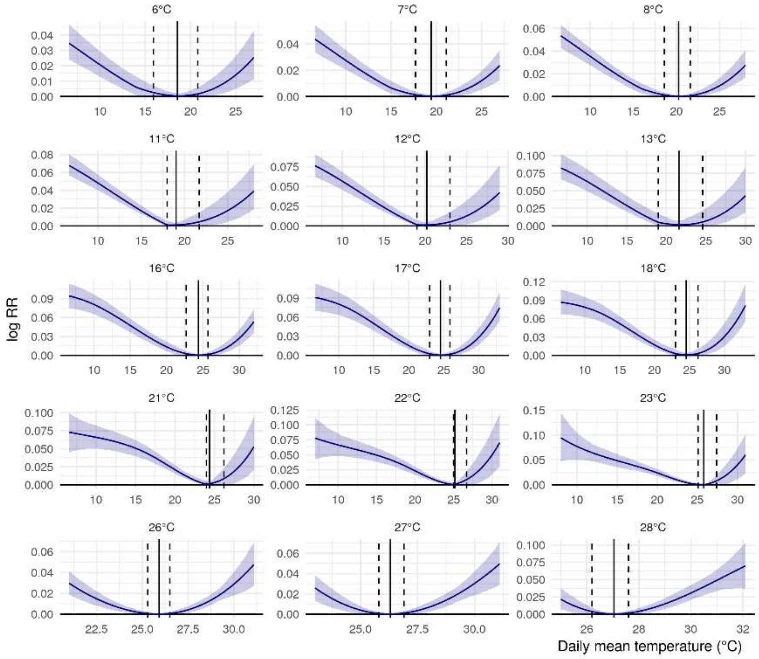

Section 3.4.1 Theoretical minimum risk exposure level

Generally, we found higher TMRELs in warmer locations (figure S4). The highest TMRELs were found in low-latitude areas, such as north Africa, south Asia, and Australia, and the lowest TMRELs were found in high-latitude areas of the northern hemisphere, such as Russia, Scandinavia, and Canada. We observed several clusters of relatively low TMRELs surrounded by higher TMRELs, especially in sub-Saharan Africa, Asia, and South America. Comparing TMRELs in 1990 and 2019, we also see a global increase, reflecting changes in cause of death composition.

Figure S4: Global distribution of theoretical minimum risk exposure level (TMREL) in 1990 and 2019

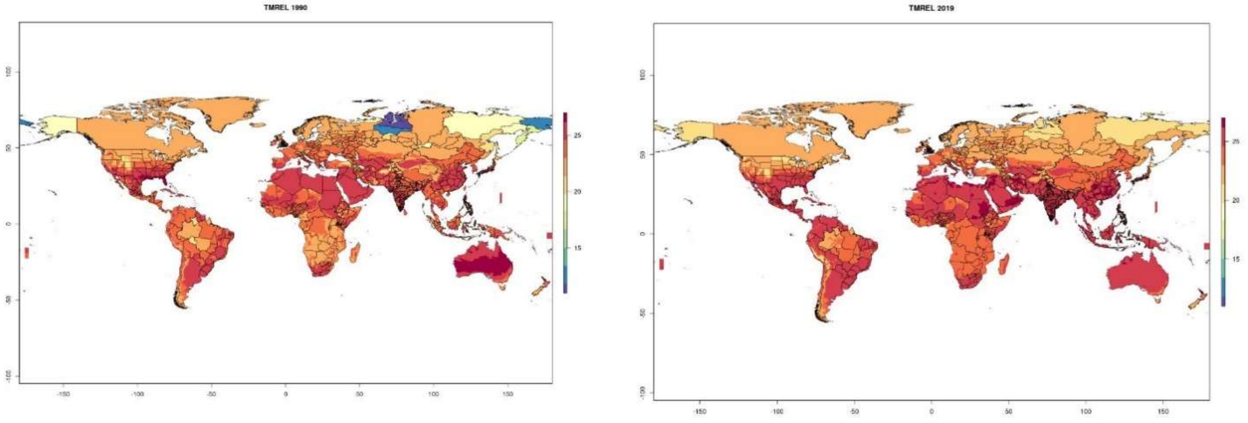

Figure S5: GBD super-regions

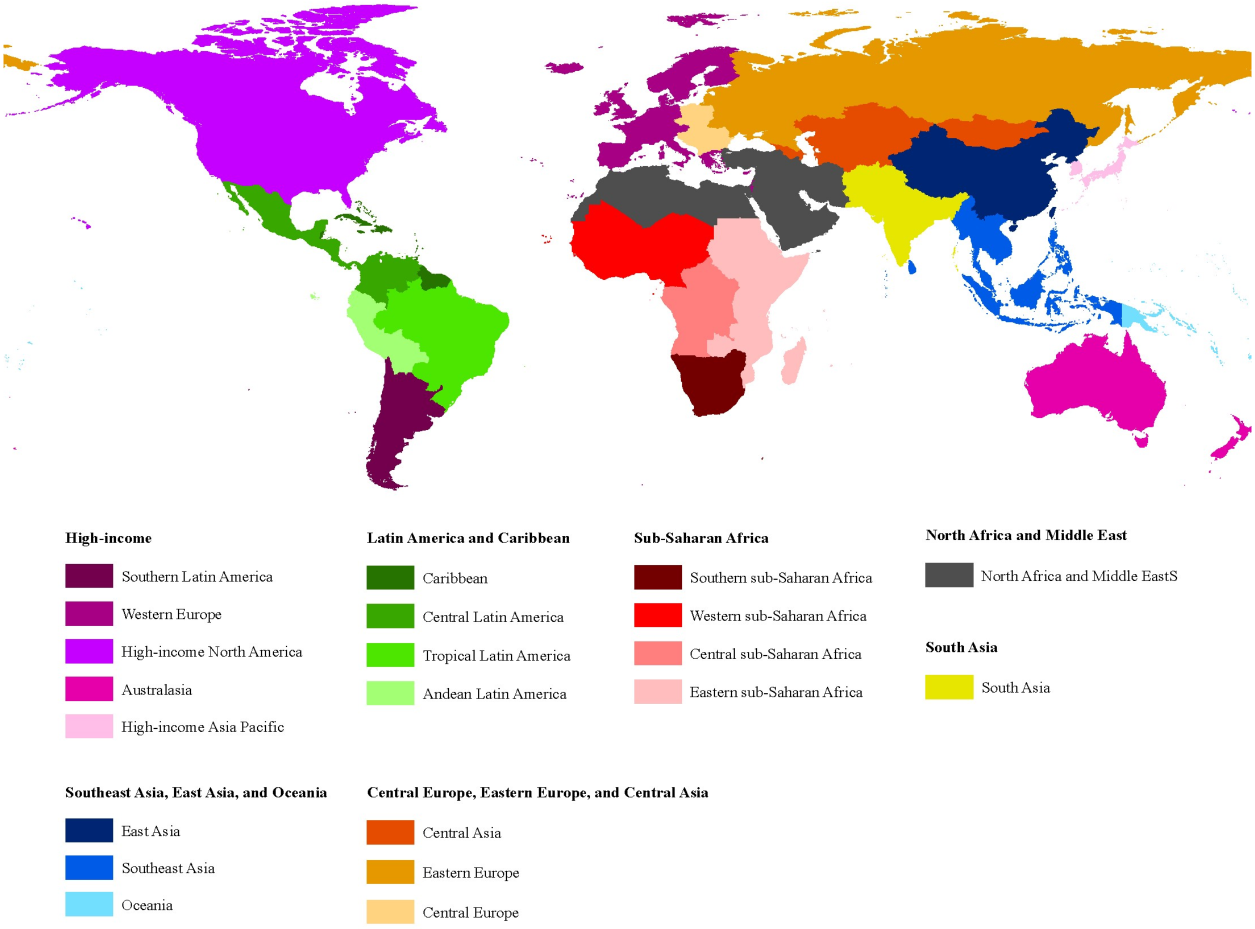

Figure S6: Isopleth diagrams depicting the risk surface of the log RR of mortality of selected causes along daily and annual mean temperature.

Green and blue dots illustrate data points; red dotted line illustrates trough of surface.

Lower respiratory infections

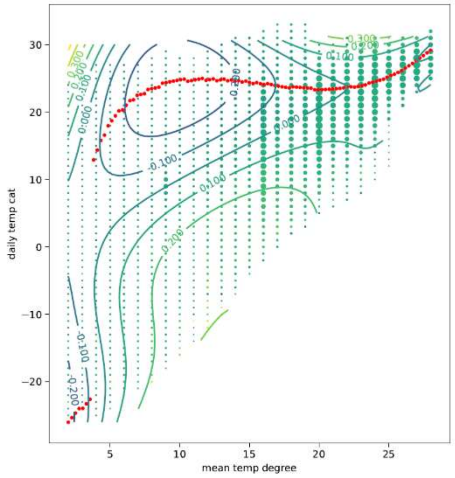

Ischaemic heart disease

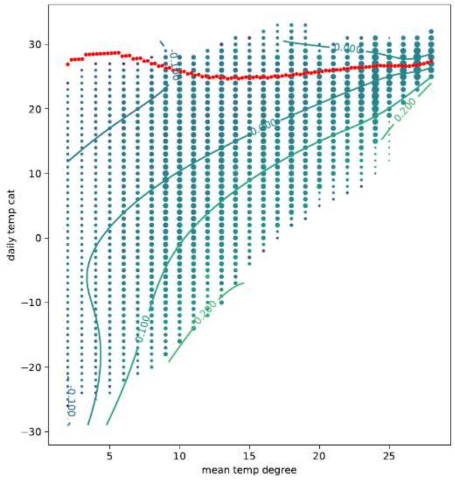

Stroke

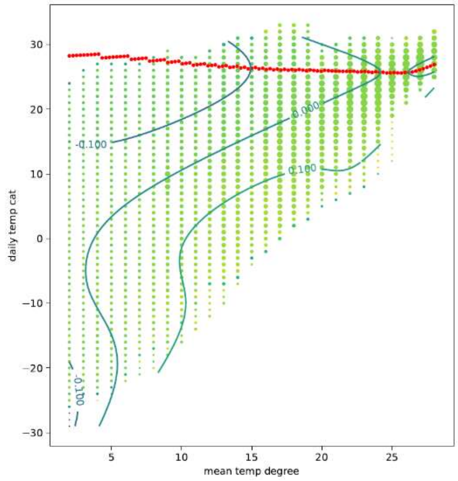

Hypertensive heart disease

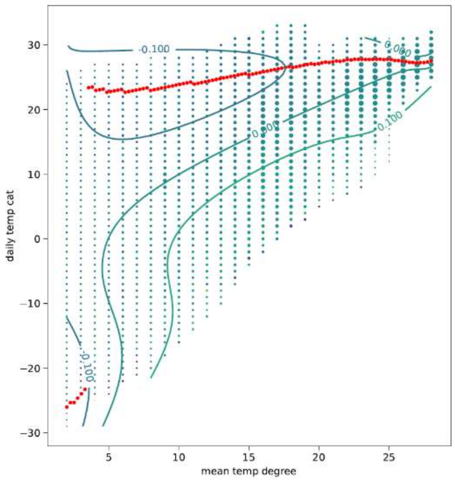

Cardiomyopathy and myocarditis

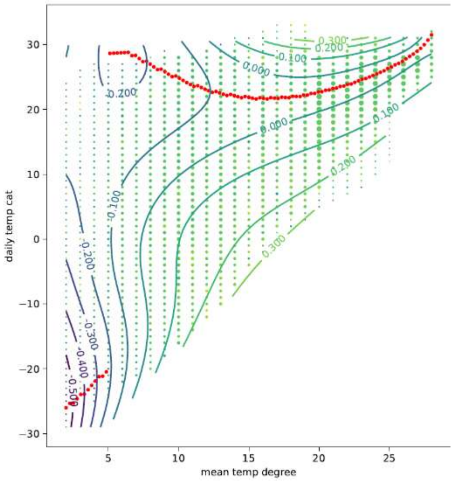

COPD

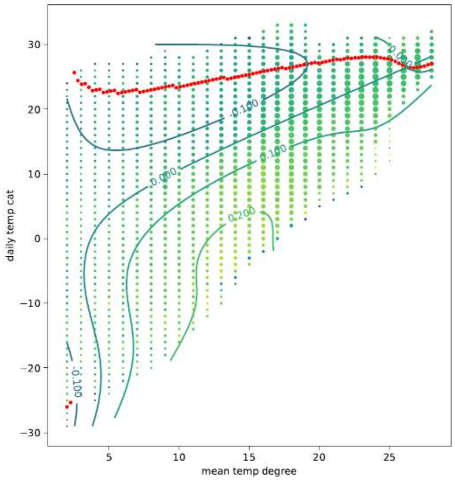

Diabetes

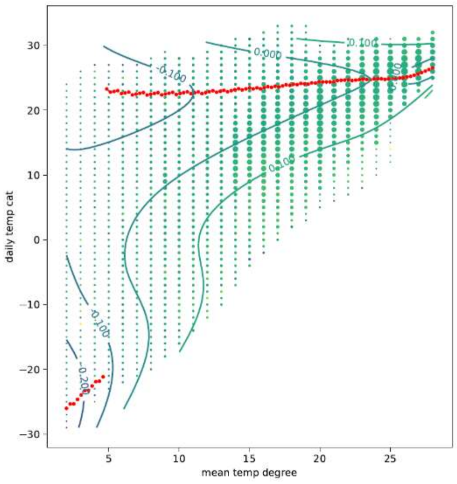

Chronic kidney disease

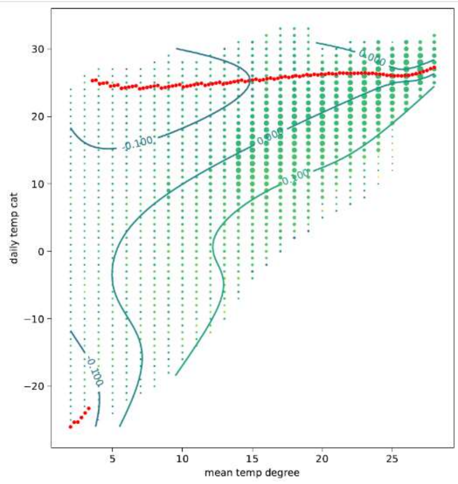

Road injuries

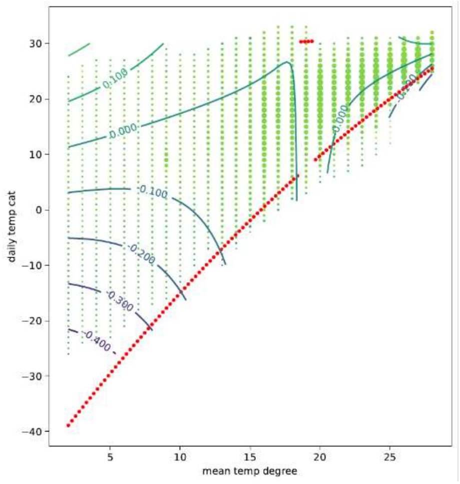

Other transport-related injuries

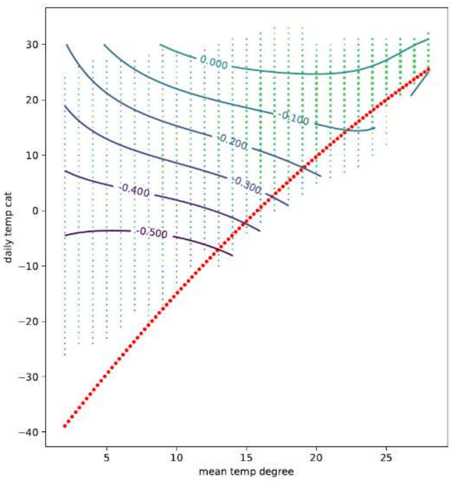

Drowning

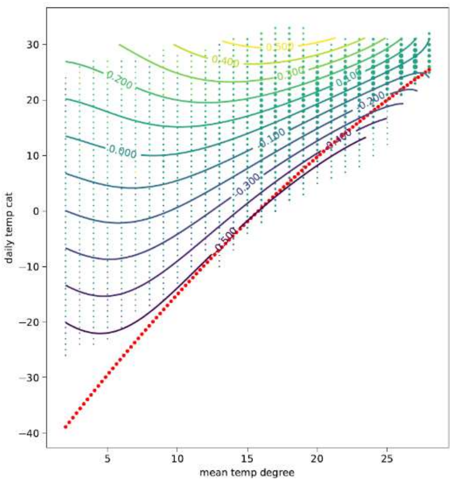

Mechanical injuries

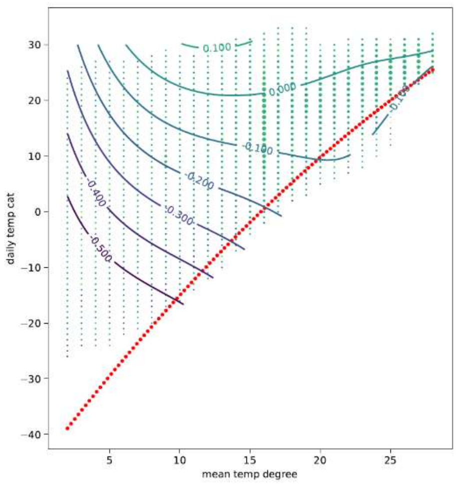

Animal-related injuries

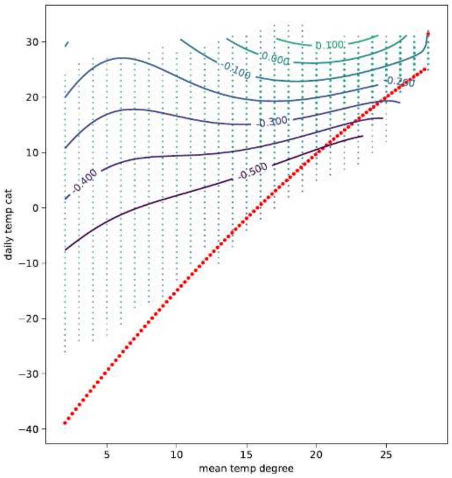

Disaster-related injuries

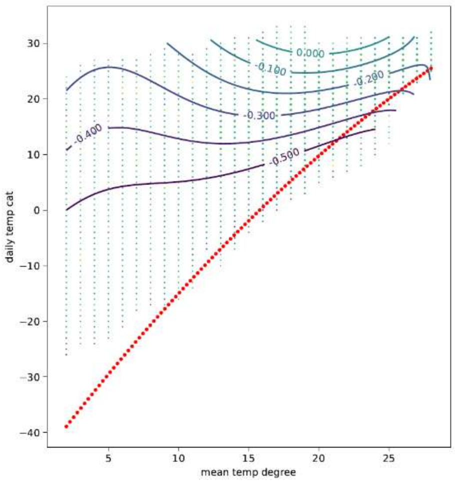

Other unintentional injuries

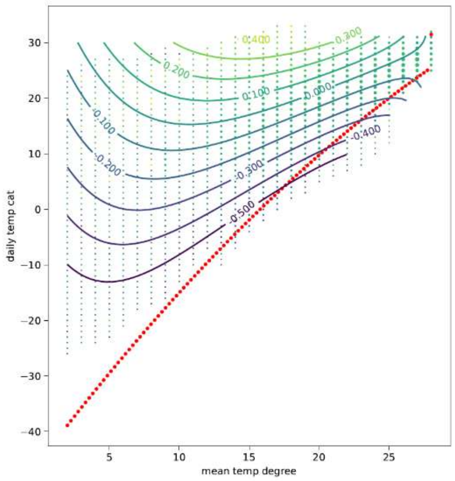

Suicide

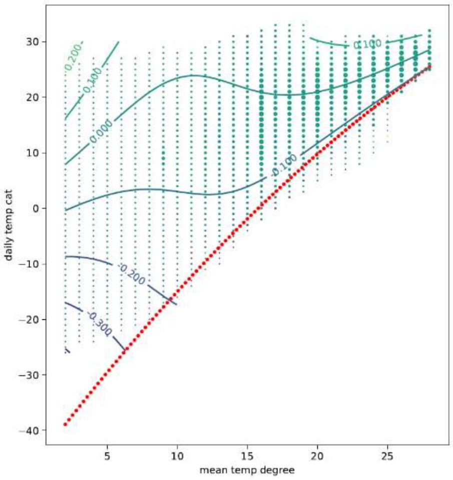

Homicide

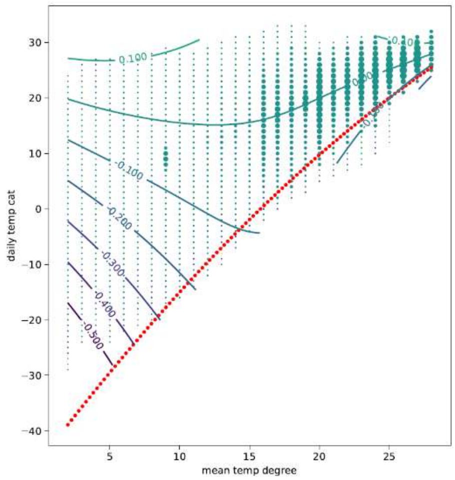

Figure S7: Exposure-response curves displaying the relationship between daily mean temperature and the log RR for mortality from lower respiratory infections.

The RR is referenced to the theoretical minimum risk exposure level (TMREL), which represents the minimum mortality temperature for death-weighted multi-cause curves in each mean annual temperature category. The black solid line depicts the location of the TMREL in each mean annual temperature category, with dashed lines depicting 95% UI of the TMREL.

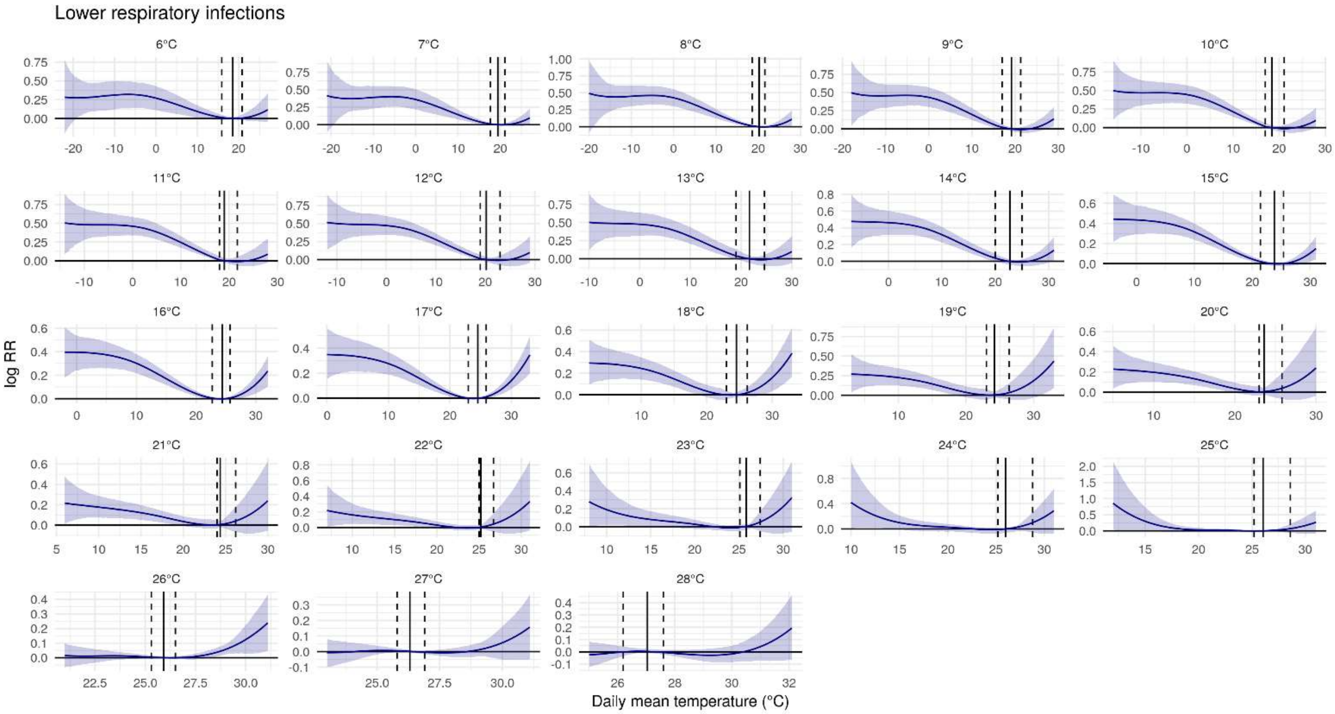

Figure S8: Exposure-response curves displaying the relationship between daily mean temperature and the log RR for mortality from ischaemic heart disease (IHD).

The RR is referenced to the theoretical minimum risk exposure level (TMREL), which represents the minimum mortality temperature for death-weighted multi-cause curves in each mean annual temperature category. The black solid line depicts the location of the TMREL in each mean annual temperature category, with dashed lines depicting 95% UI of the TMREL.

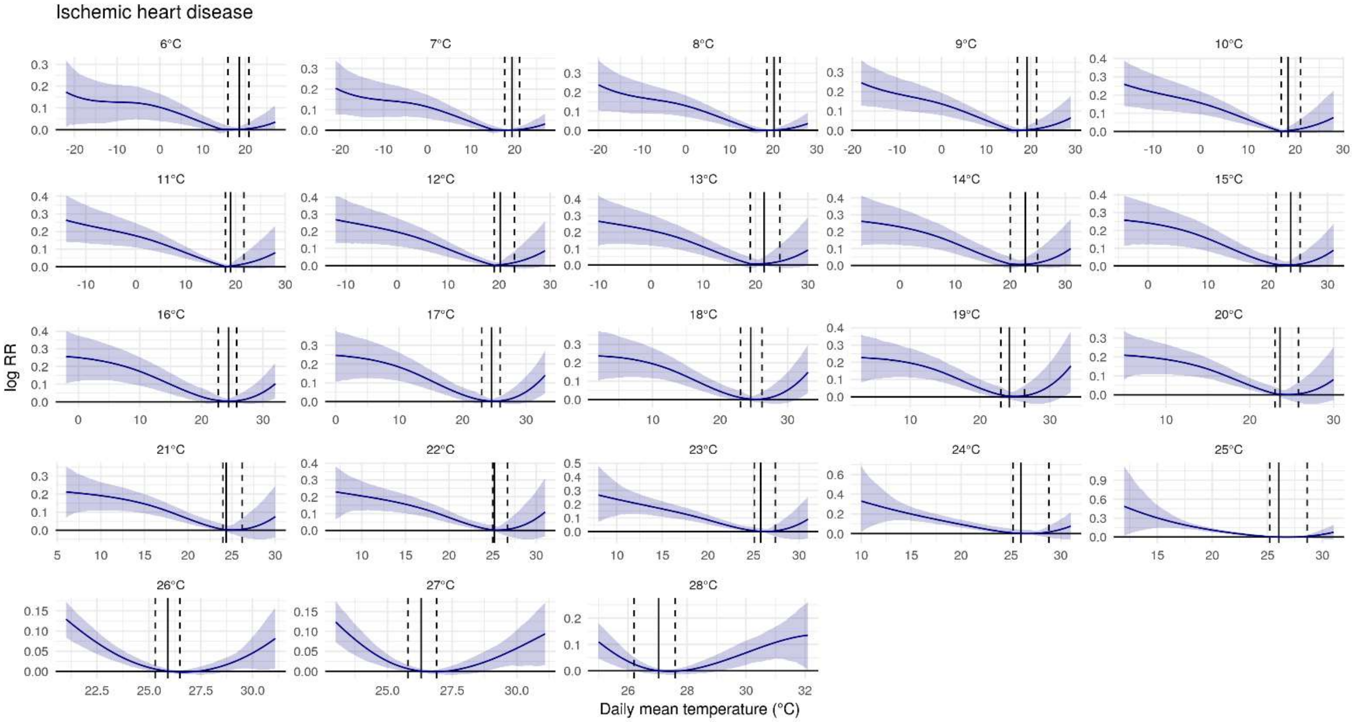

Figure S9: Exposure-response curves displaying the relationship between daily mean temperature and the log RR for mortality from stroke.

The RR is referenced to the theoretical minimum risk exposure level (TMREL), which represents the minimum mortality temperature for death-weighted multi-cause curves in each mean annual temperature category. The black solid line depicts the location of the TMREL in each mean annual temperature category, with dashed lines depicting 95% UI of the TMREL.

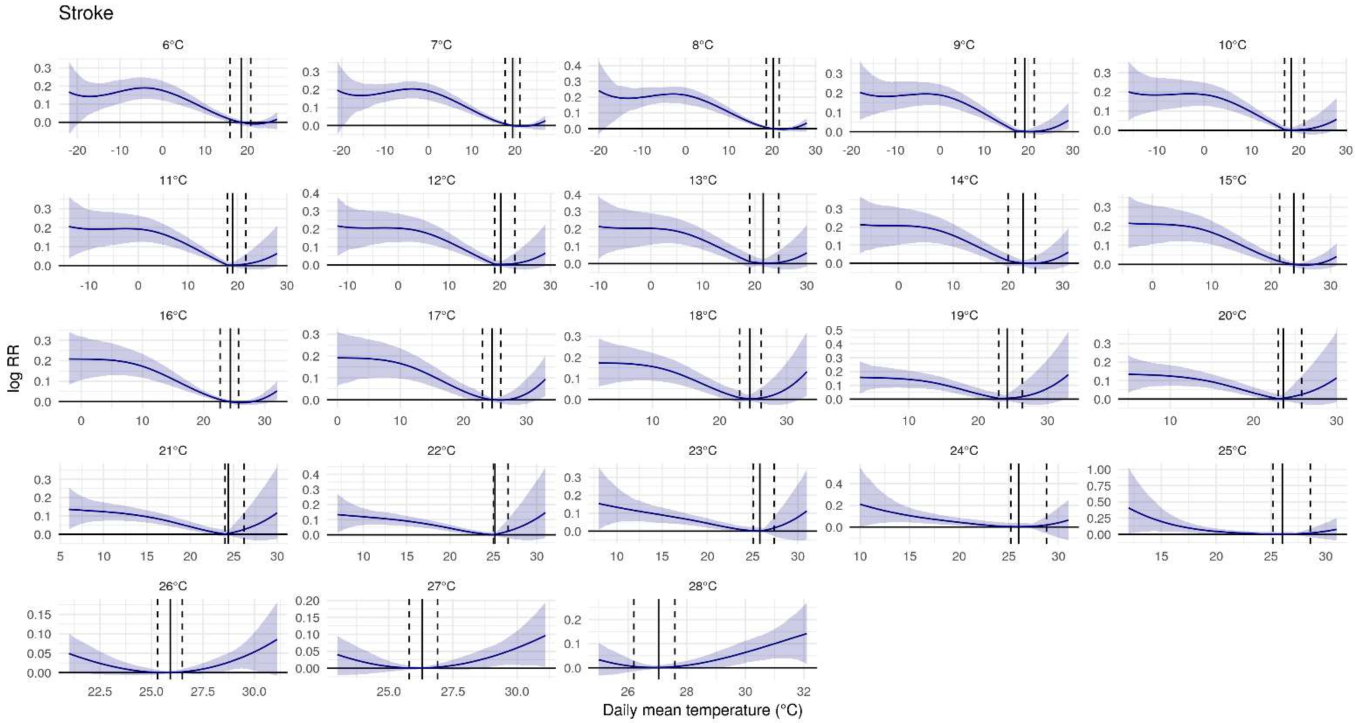

Figure S10: Exposure-response curves displaying the relationship between daily mean temperature and the log RR for mortality from hypertensive heart disease.

The RR is referenced to the theoretical minimum risk exposure level (TMREL), which represents the minimum mortality temperature for death-weighted multi-cause curves in each mean annual temperature category. The black solid line depicts the location of the TMREL in each mean annual temperature category, with dashed lines depicting 95% UI of the TMREL.

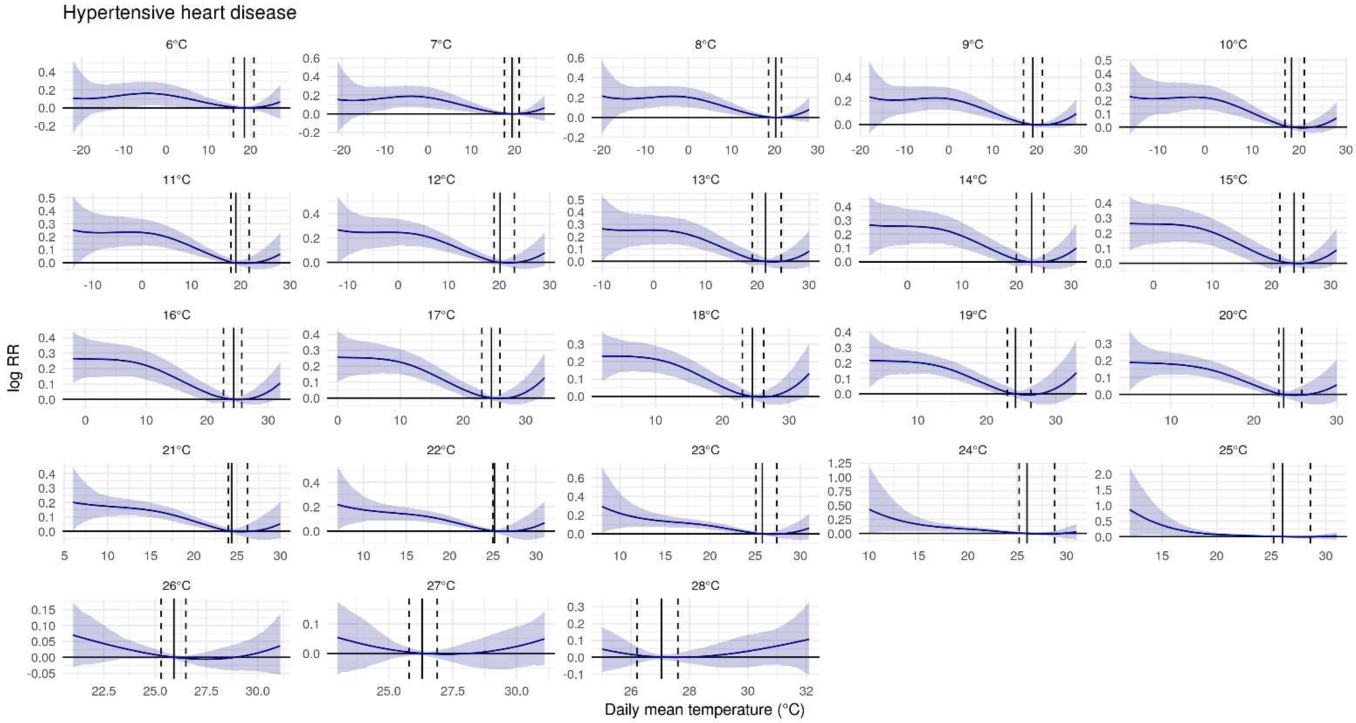

Figure S11: Exposure-response curves displaying the relationship between daily mean temperature and the log RR for mortality from cardiomyopathy and myocarditis.

The RR is referenced to the theoretical minimum risk exposure level (TMREL), which represents the minimum mortality temperature for death-weighted multi-cause curves in each mean annual temperature category. The black solid line depicts the location of the TMREL in each mean annual temperature category, with dashed lines depicting 95% UI of the TMREL.

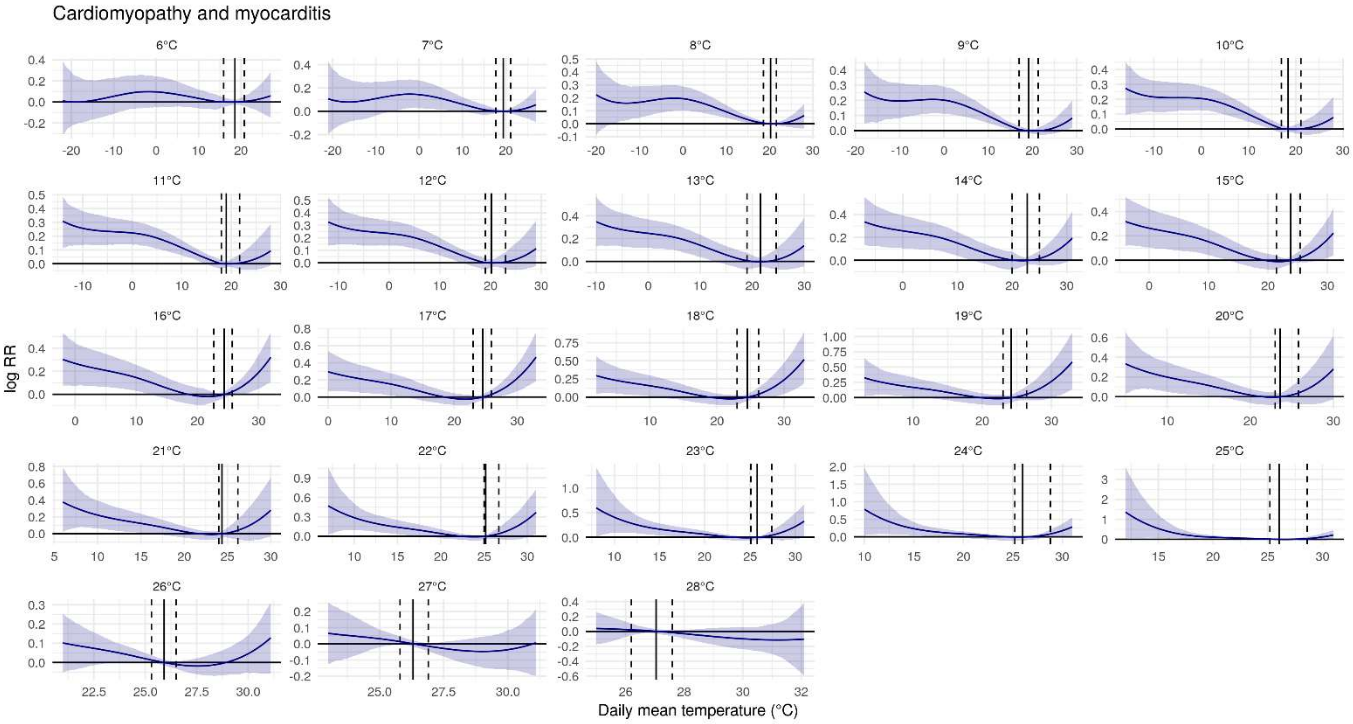

Figure S12: Exposure-response curves displaying the relationship between daily mean temperature and the log RR for mortality from chronic obstructive pulmonary disease.

The RR is referenced to the theoretical minimum risk exposure level (TMREL), which represents the minimum mortality temperature for death-weighted multi-cause curves in each mean annual temperature category. The black solid line depicts the location of the TMREL in each mean annual temperature category, with dashed lines depicting 95% UI of the TMREL.

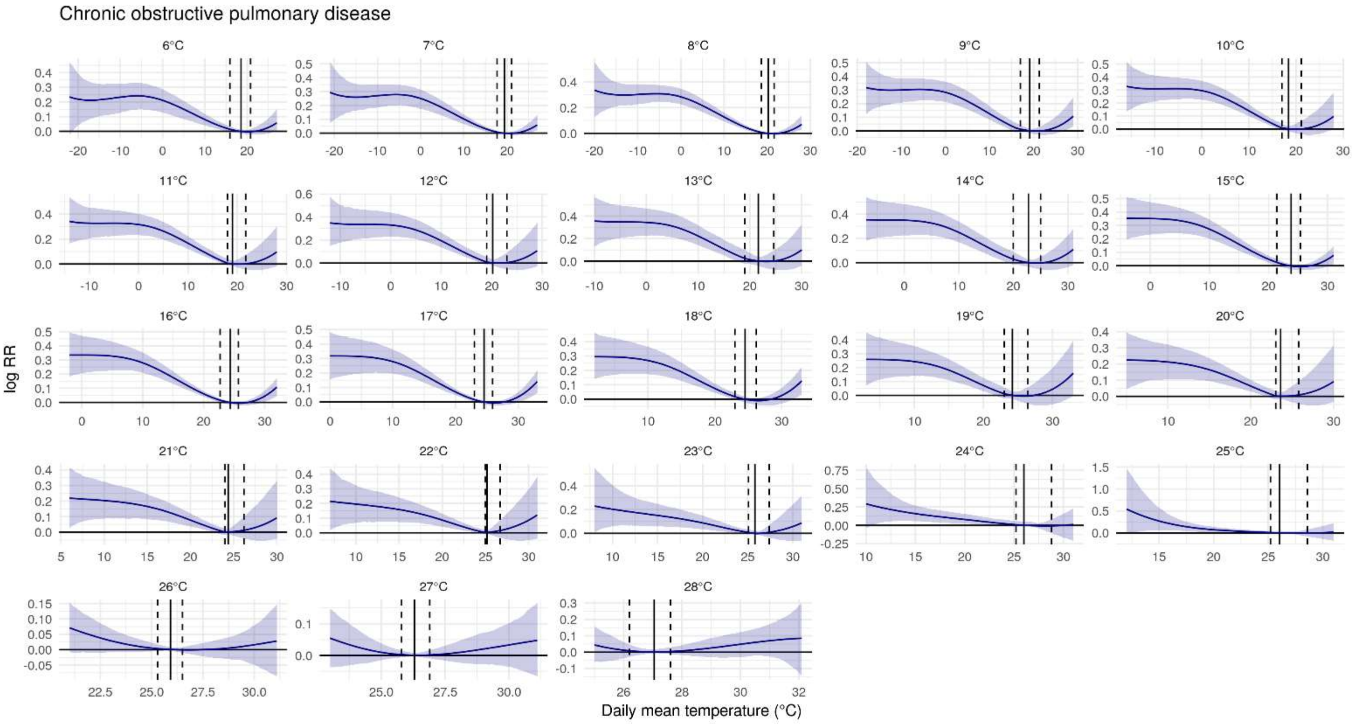

Figure S13: Exposure-response curves displaying the relationship between daily mean temperature and the log RR for mortality from diabetes mellitus.

The RR is referenced to the theoretical minimum risk exposure level (TMREL), which represents the minimum mortality temperature for death-weighted multi-cause curves in each mean annual temperature category. The black solid line depicts the location of the TMREL in each mean annual temperature category, with dashed lines depicting 95% UI of the TMREL.

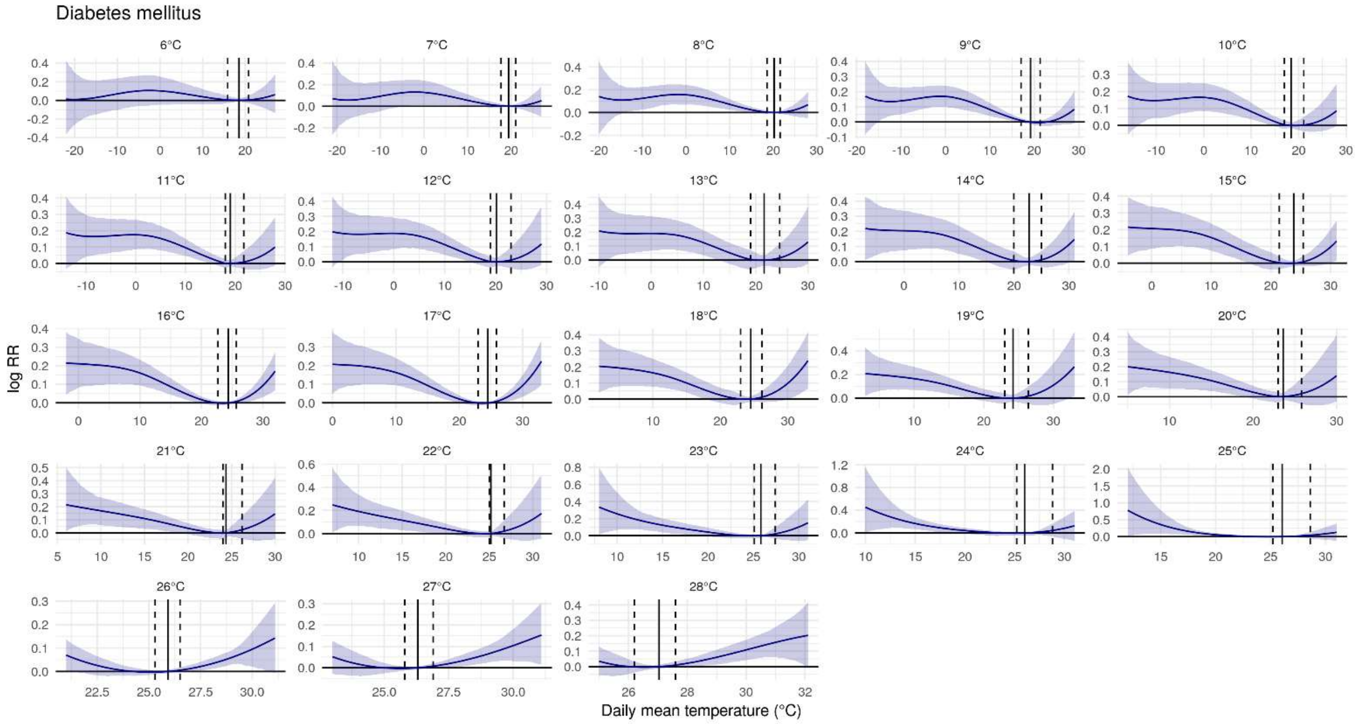

Figure S14: Exposure-response curves displaying the relationship between daily mean temperature and the log RR for mortality from chronic kidney disease.

The RR is referenced to the theoretical minimum risk exposure level (TMREL), which represents the minimum mortality temperature for death-weighted multi-cause curves in each mean annual temperature category. The black solid line depicts the location of the TMREL in each mean annual temperature category, with dashed lines depicting 95% UI of the TMREL.

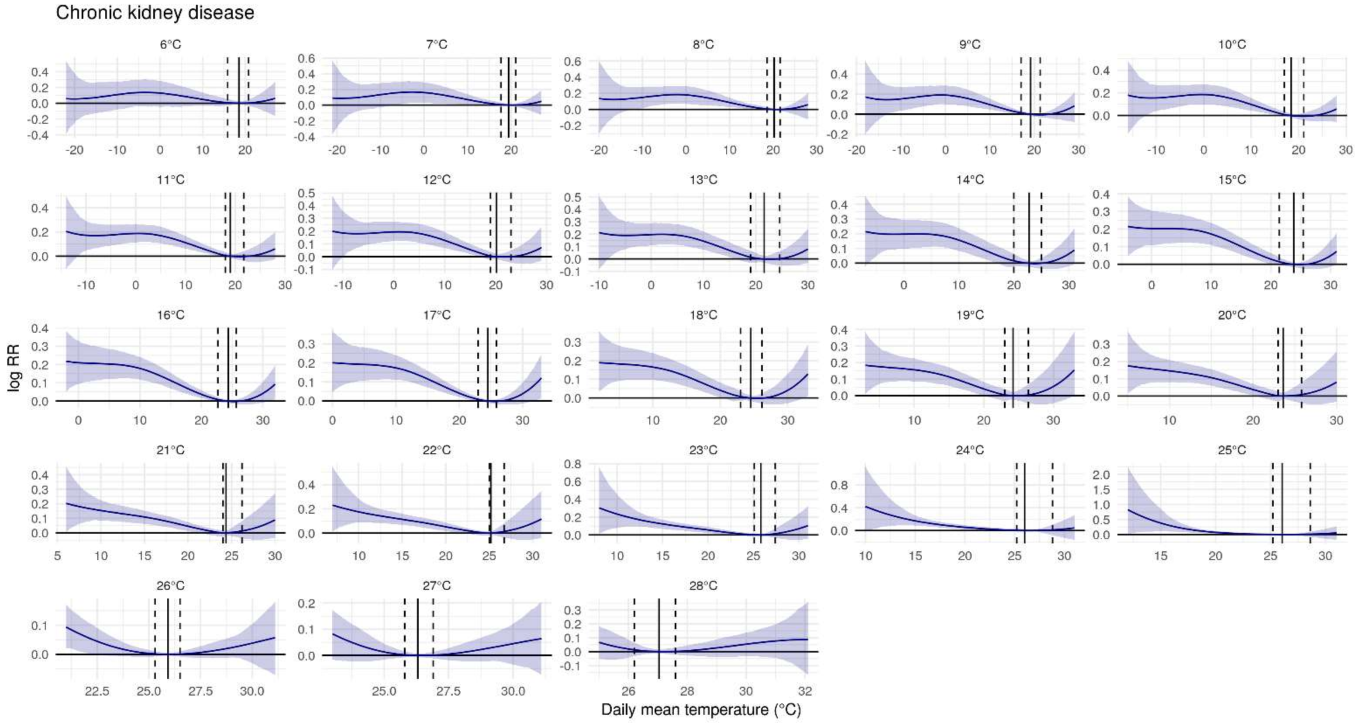

Figure S15: Exposure-response curves displaying the relationship between daily mean temperature and the log RR for mortality from road injuries.
The RR is referenced to the theoretical minimum risk exposure level (TMREL), which represents the minimum mortality temperature for death-weighted multi-cause curves in each mean annual temperature category. The black solid line depicts the location of the TMREL in each mean annual temperature category, with dashed lines depicting 95% UI of the TMREL.

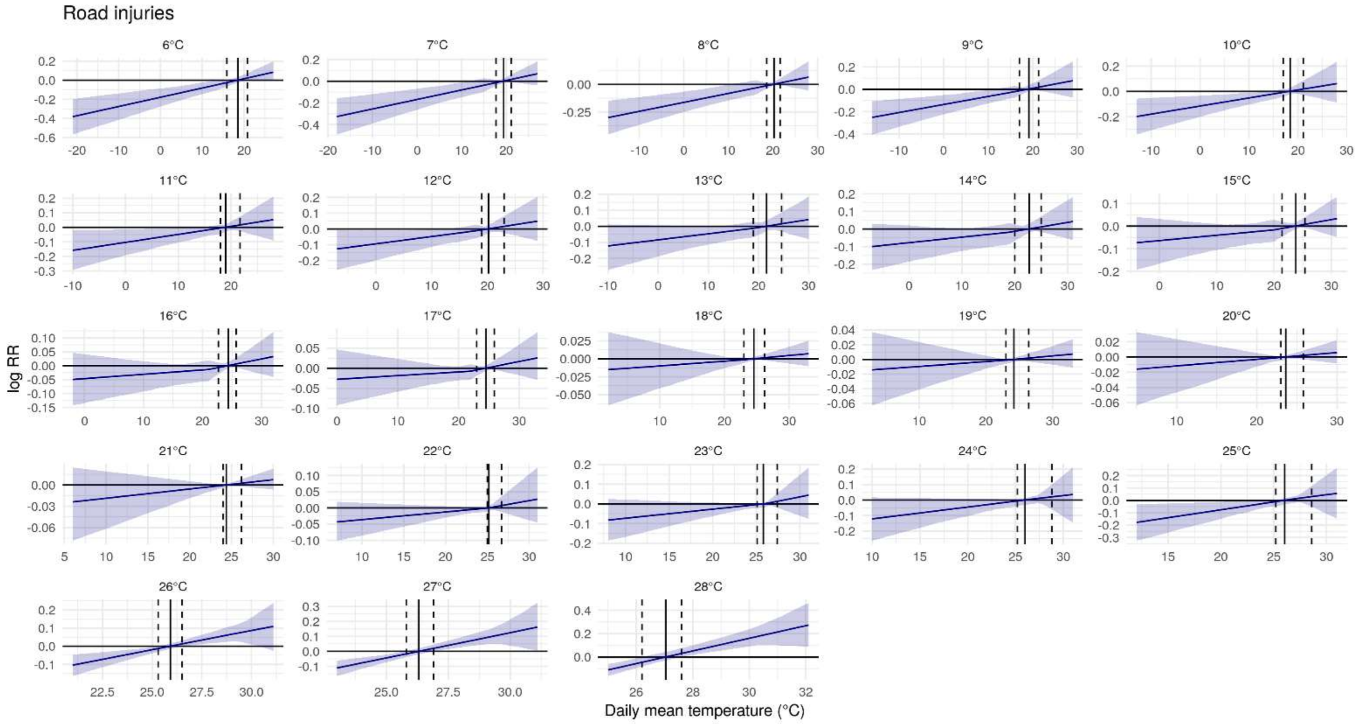

Figure S16: Exposure-response curves displaying the relationship between daily mean temperature and the log RR for mortality from other transport-related injuries.
The RR is referenced to the theoretical minimum risk exposure level (TMREL), which represents the minimum mortality temperature for death-weighted multi-cause curves in each mean annual temperature category. The black solid line depicts the location of the TMREL in each mean annual temperature category, with dashed lines depicting 95% UI of the TMREL.

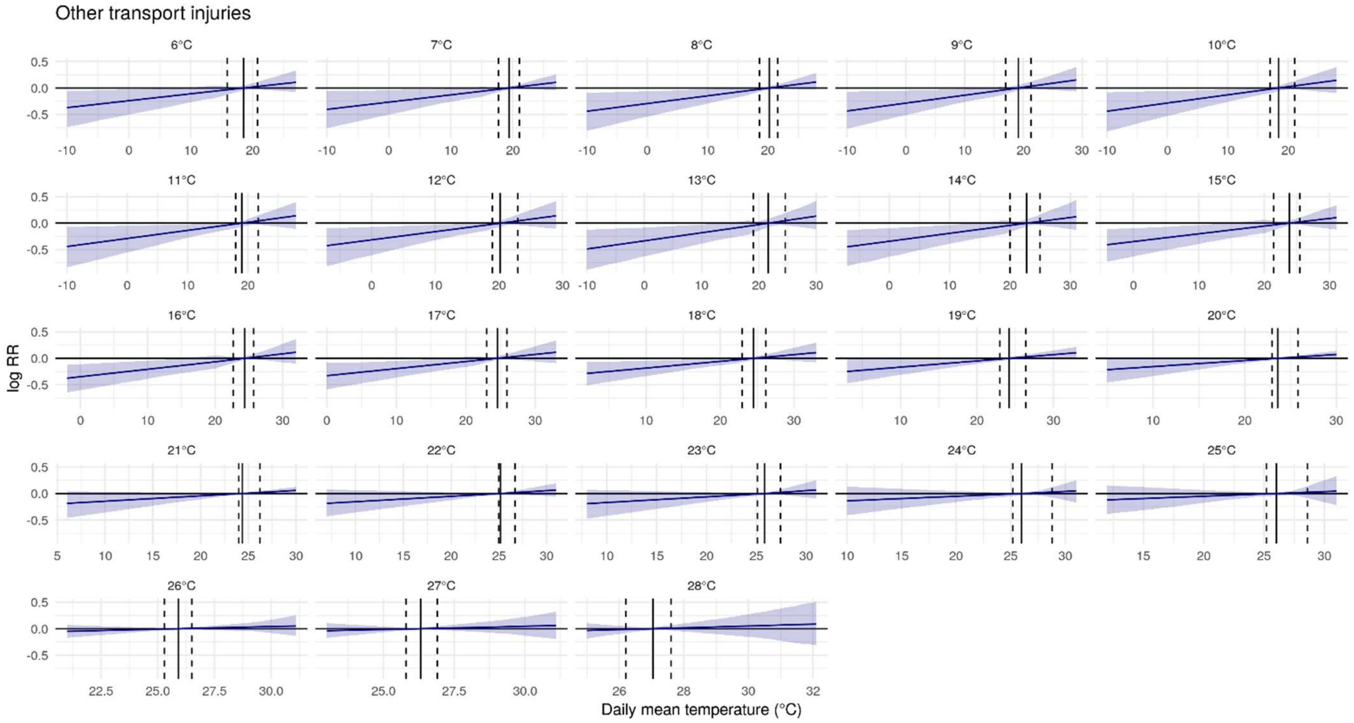

Figure S17: Exposure-response curves displaying the relationship between daily mean temperature and the log RR for mortality from drowning.

The RR is referenced to the theoretical minimum risk exposure level (TMREL), which represents the minimum mortality temperature for death-weighted multi-cause curves in each mean annual temperature category. The black solid line depicts the location of the TMREL in each mean annual temperature category, with dashed lines depicting 95% UI of the TMREL.

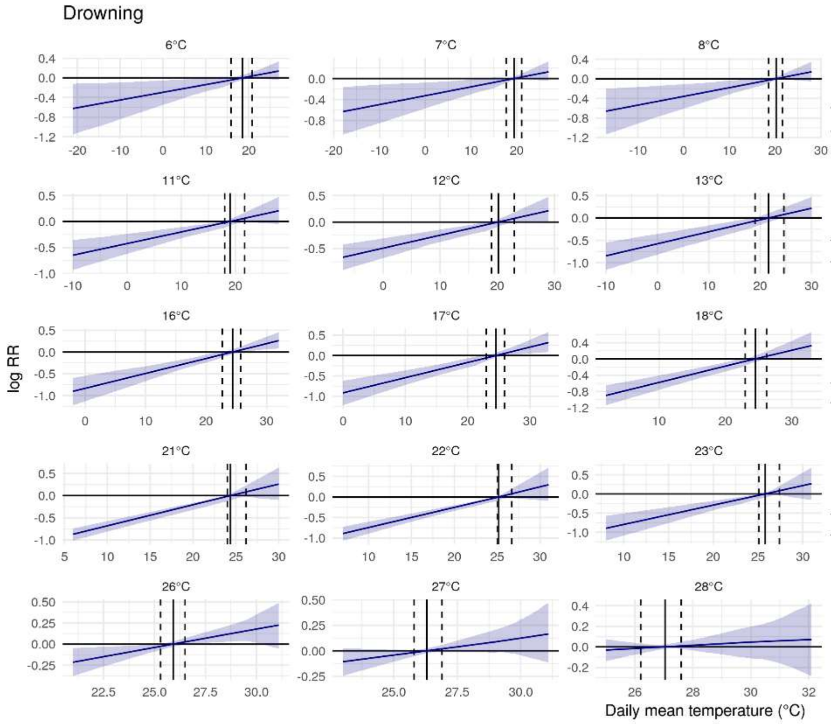

Figure S18: Exposure-response curves displaying the relationship between daily mean temperature and the log RR for mortality from mechanical injuries (exposure to mechanical forces).

The RR is referenced to the theoretical minimum risk exposure level (TMREL), which represents the minimum mortality temperature for death-weighted multi-cause curves in each mean annual temperature category. The black solid line depicts the location of the TMREL in each mean annual temperature category, with dashed lines depicting 95% UI of the TMREL.

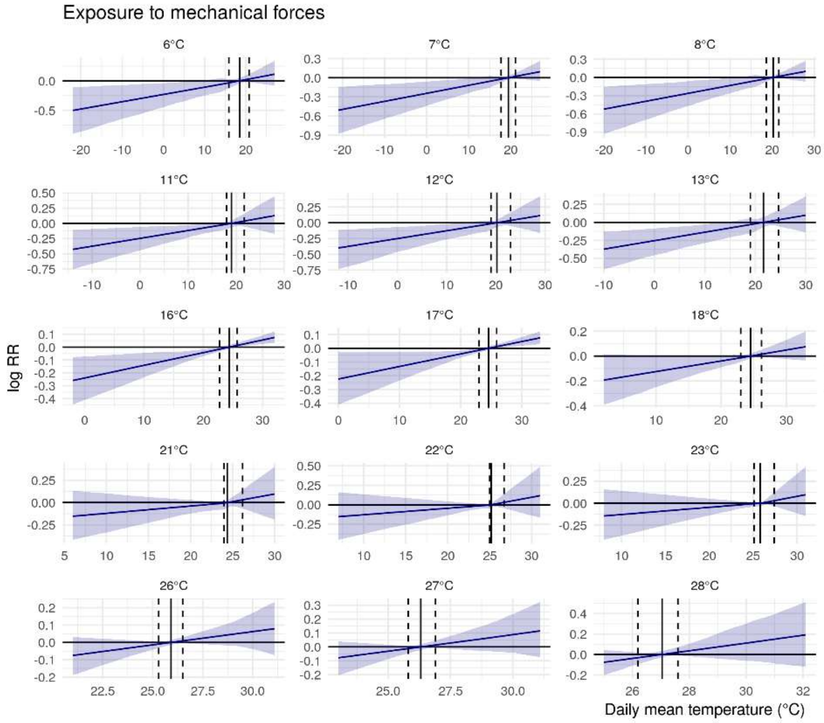

Figure S19: Exposure-response curves displaying the relationship between daily mean temperature and the log RR for mortality from animal-related injuries (animal contact).

The RR is referenced to the theoretical minimum risk exposure level (TMREL), which represents the minimum mortality temperature for death-weighted multi-cause curves in each mean annual temperature category. The black solid line depicts the location of the TMREL in each mean annual temperature category, with dashed lines depicting 95% UI of the TMREL.

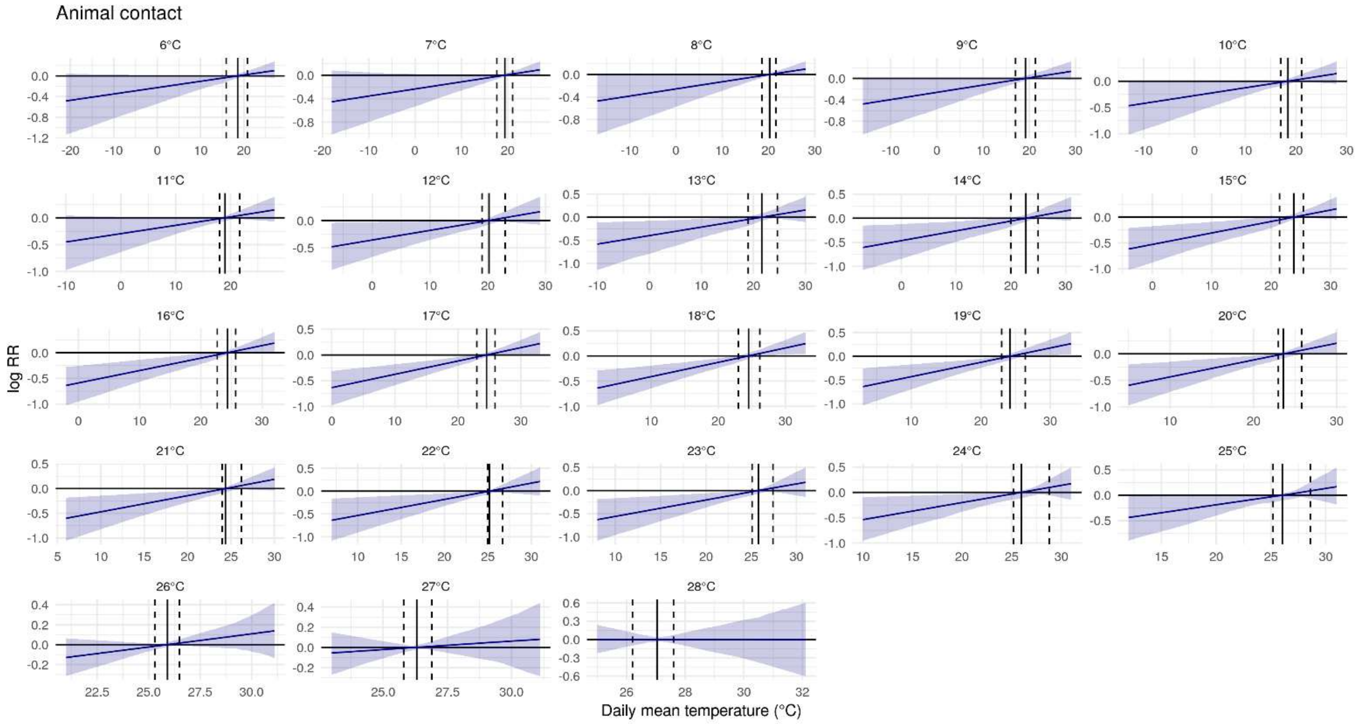

Figure S20: Exposure-response curves displaying the relationship between daily mean temperature and the log RR for mortality from disaster-related injuries (exposure to forces of nature).

The RR is referenced to the theoretical minimum risk exposure level (TMREL), which represents the minimum mortality temperature for death-weighted multi-cause curves in each mean annual temperature category. The black solid line depicts the location of the TMREL in each mean annual temperature category, with dashed lines depicting 95% UI of the TMREL.

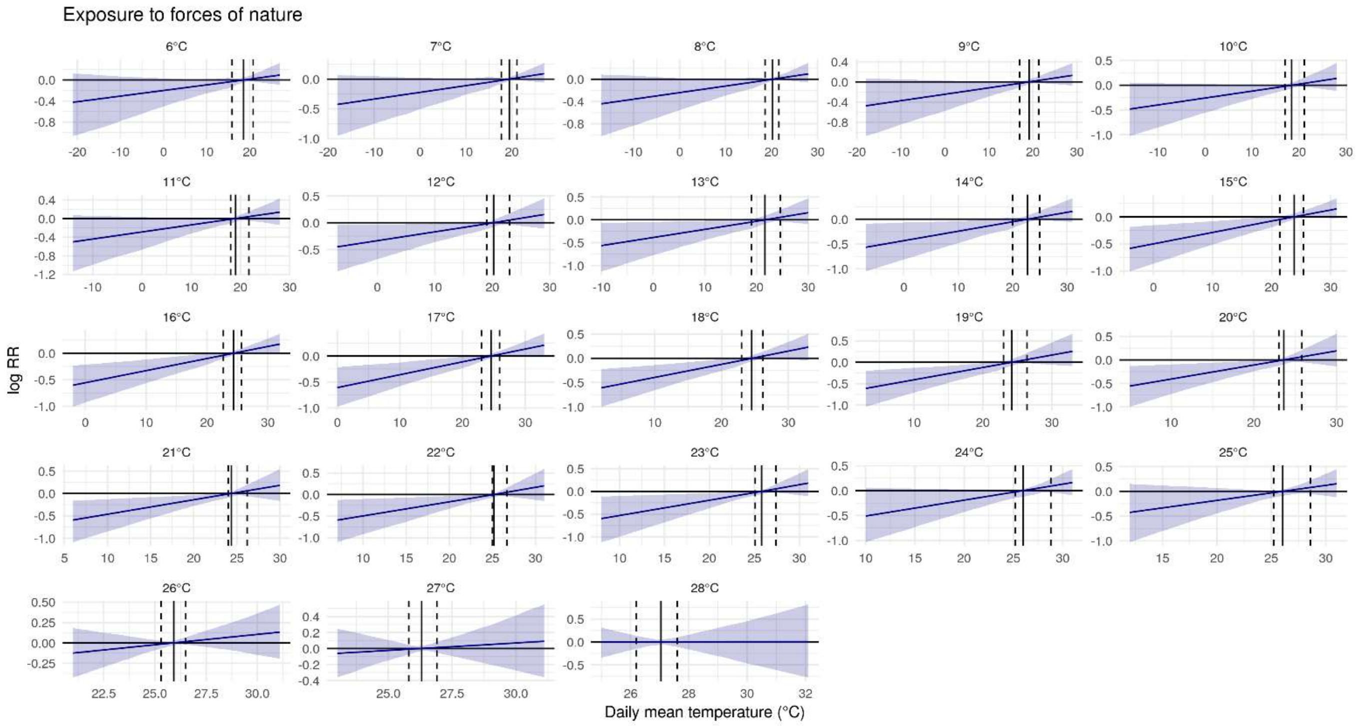

Figure S21: Exposure-response curves displaying the relationship between daily mean temperature and the log RR for mortality from other unintentional injuries.

The RR is referenced to the theoretical minimum risk exposure level (TMREL), which represents the minimum mortality temperature for death-weighted multi-cause curves in each mean annual temperature category. The black solid line depicts the location of the TMREL in each mean annual temperature category, with dashed lines depicting 95% UI of the TMREL.

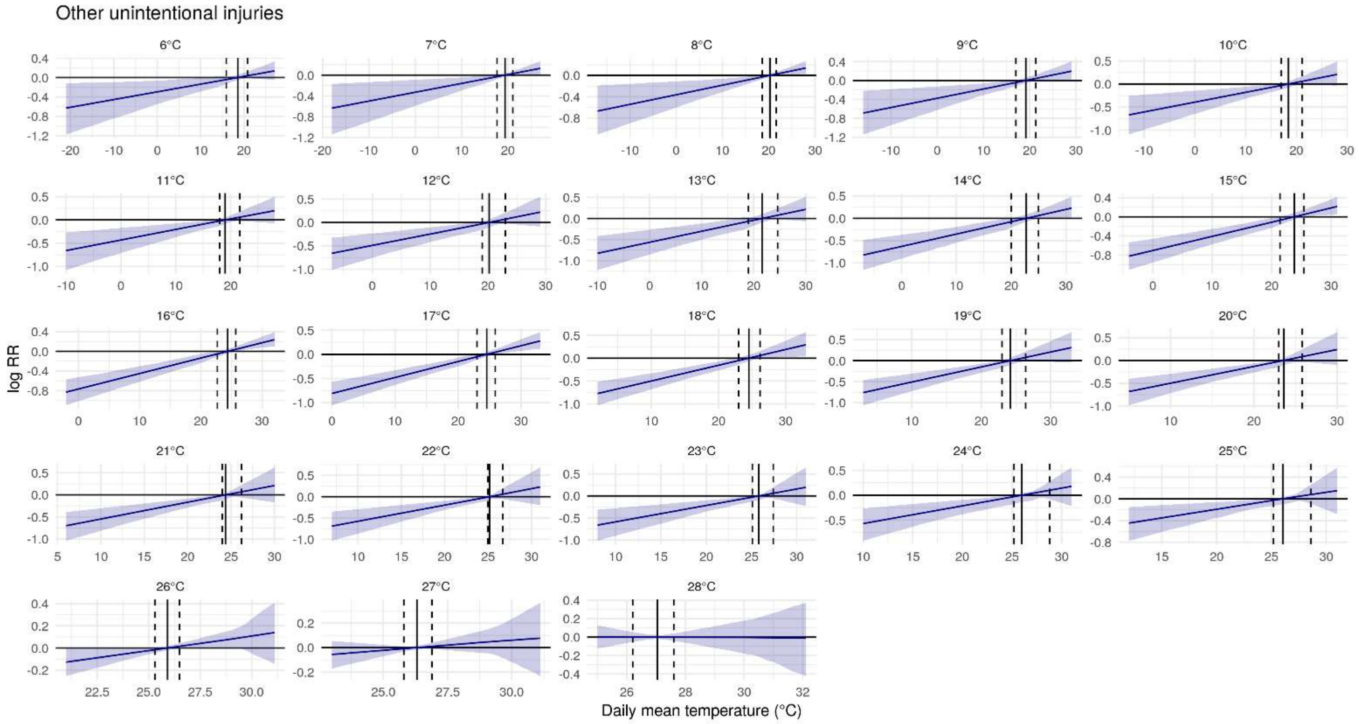

Figure S22: Exposure-response curves displaying the relationship between daily mean temperature and the log RR for mortality from suicide (self-harm).

The RR is referenced to the theoretical minimum risk exposure level (TMREL), which represents the minimum mortality temperature for death-weighted multi-cause curves in each mean annual temperature category. The black solid line depicts the location of the TMREL in each mean annual temperature category, with dashed lines depicting 95% UI of the TMREL.

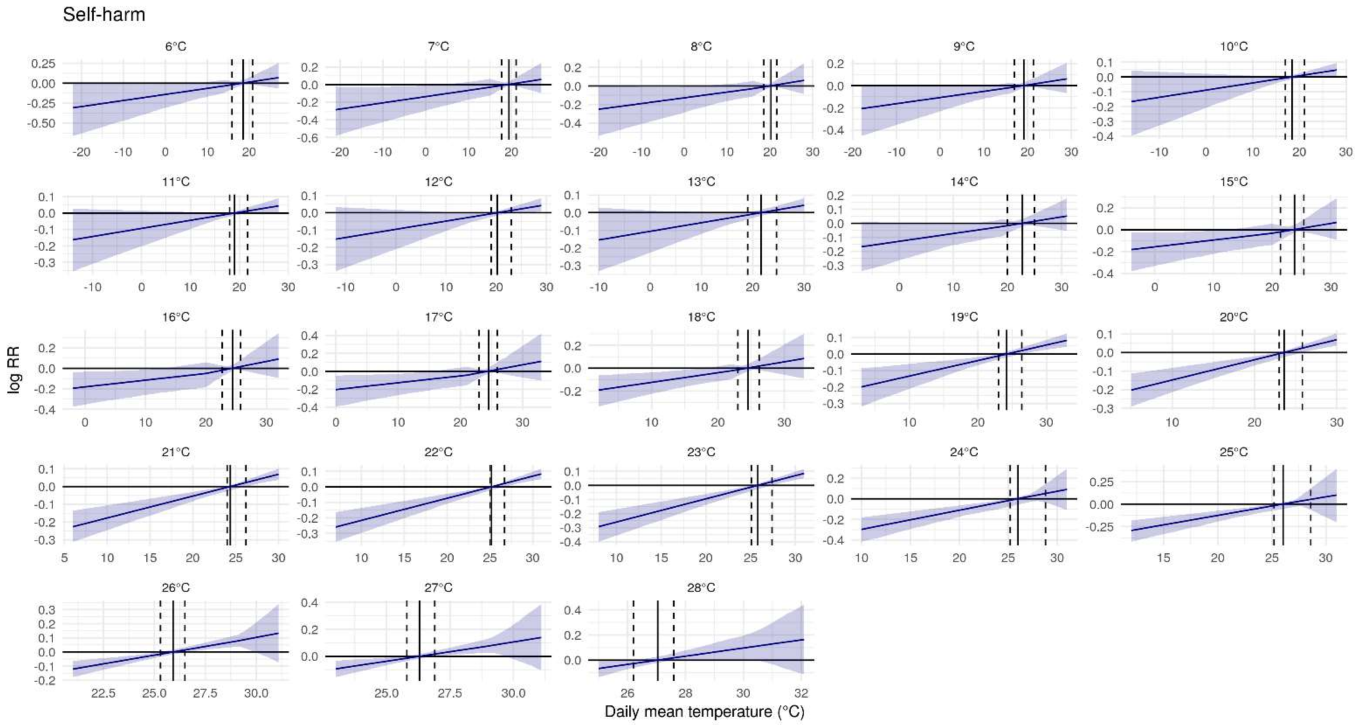

Figure S23: Exposure-response curves displaying the relationship between daily mean temperature and the log RR for mortality from homicide (interpersonal violence).

The RR is referenced to the theoretical minimum risk exposure level (TMREL), which represents the minimum mortality temperature for death-weighted multi-cause curves in each mean annual temperature category. The black solid line depicts the location of the TMREL in each mean annual temperature category, with dashed lines depicting 95% UI of the TMREL.

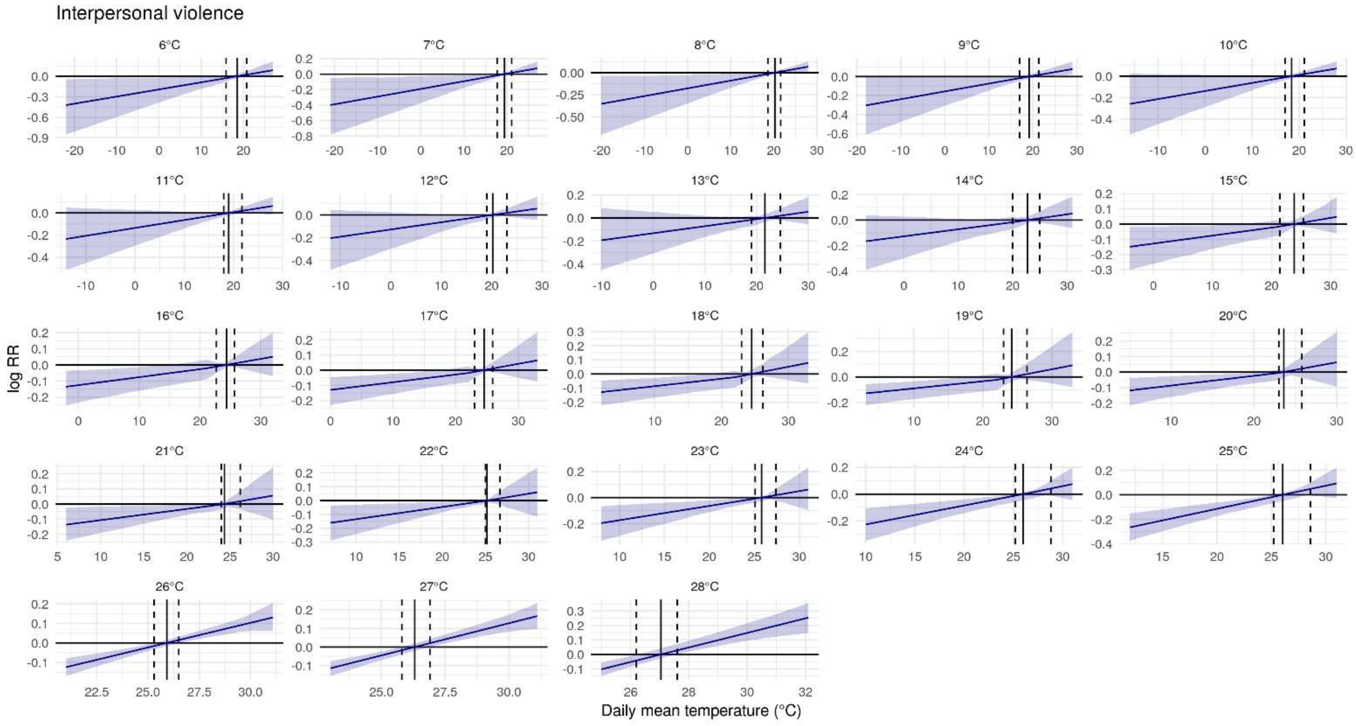

Figure S24: Intra-country differences in the composition of deaths and DALYs attributable to high and low temperatures Sao Paulo (Brazil), Piaui (Brazil), Michigan (USA), and Arizona (USA), Mexico City (MEX), and Campeche (MEX) in 2019.

Note that the x-axis scales vary by location.

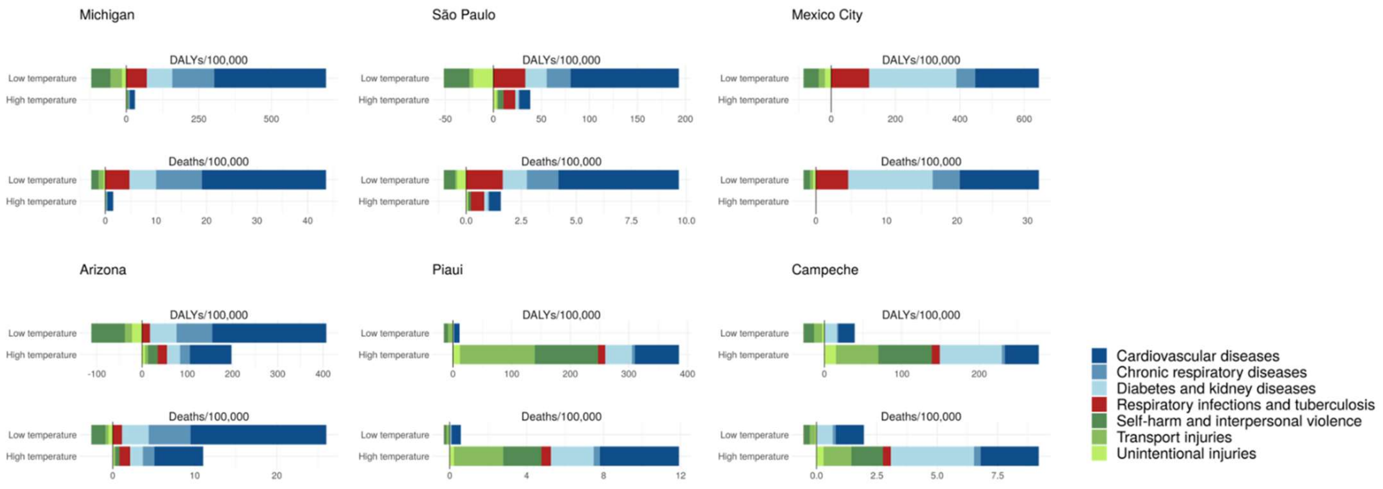

Figure S24 displays the cause composition of heat and cold effects for selected subnational locations in the USA, Brazil, and Mexico. Michigan, a northern USA state, displays strong cold effects with 40·9 deaths (33·4 –48·7) and 569·1 DALYs (457·5 – 690·2) per 100,000. These cold effects are primarily driven by cardio-respiratory and metabolic disease. Cold exerts a protective effect on external causes, and the cold-attributable burden of self-harm and interpersonal violence, as well as injuries in Michigan, is negative with -2·71 deaths (-3·63– -1·93) and -118·9 DALYs (-158·6– -85·3) per 100,000. Arizona, in the southwestern USA, displays substantially higher heat-attributable burden with 11·0 deaths (5·36–17·9) and 196·3 DALYs (104·5–

307·0) per 100,000 which is mostly driven by non-external causes, but external causes contribute a small share. São Paulo, in Brazil, exhibits substantial cold-related burden with 8·65 deaths (6·90– 10·7) and 141·8 DALYs (110·3-178·4) per 100,000. Contrary to that, Piauí in the north of the country displays only a small cold-attributable burden of non-external causes that is offset by protective cold effects on external causes; the total cold-related burden in Piauí amounts to 0·28 deaths (0·052 – 0·68) and -4·07 DALYs (-11·0 –1.68) per 100,000. Piauí exhibits a large heat-attributable burden of 11·9 deaths (9·07-15·2) and 385·3 DALYs (295·7-500·3) per 100,000 driven by external as well as cardiovascular and metabolic causes. Mexico City displays an average share of cold-attributable burden with 29·8 deaths (22·3 –39.4) and 559·8 DALYs (412·6– 746·4) per 100,000 but no heat-attributable burden. Campeche, in the southeast of Mexico, shows a comparably small cold-attributable burden with 1·43 deaths (0·68–2·20) and 12·3 DALYs (-2·78–29·3) per 100,000 but a large heat-attributable burden with 9·20 deaths (6·19–12·7) and 276 8 DALYs (192·2– 376·7) per 100,000, driven equally by external and non-external causes in 2019.

Table S5: Burden estimates for high temperature, low temperature and non-optimal temperature exposure by super-region in 1990 and 2019 (95%UI displayed in parentheses)

	1990			2019		
Location	Attributable DALYs (thousands)	Attributable Deaths (thousands)	PAFs (% deaths)	Attributable DALYs (thousands)	Attributable Deaths (thousands)	PAFs (% deaths)
**High temperature**						
Global	11,113 (8,067 - 14,356)	205 (163 - 248)	0.44 (0.35-0.53)	11,630 (9,300 - 14,228)	356 (289 - 430)	0.63 (0.51-0.75)
Southeast Asia, East Asia, and Oceania	2,306 (1,821 - 2,863)	49 (40 - 60)	0.41 (0.34-0.49)	2,026 (1,657 - 2,418)	83 (68 - 101)	0.54 (0.44-0.64)
Central Europe, Eastern Europe, and Central Asia	170 (120 - 235)	5 (3 - 7)	0.11 (0.06-0.16)	239 (165 - 341)	10 (6 - 15)	0.21 (0.13-0.31)
High-income	251 (179 - 336)	11 (8 - 16)	0.14 (0.1-0.2)	309 (237 - 393)	18 (13 - 23)	0.18 (0.13-0.24)
Latin America and Caribbean	290 (211 - 384)	5 (4 - 6)	0.21 (0.16-0.26)	478 (385 - 583)	13 (11 - 16)	0.37 (0.31-0.45)
North Africa and Middle East	1,189 (919 - 1,517)	23 (18 - 28)	0.91 (0.73-1.09)	1,130 (862 - 1,411)	36 (28 - 45)	1.16 (0.92-1.44)
South Asia	5,391 (3,247 - 7,714)	90 (61 - 121)	0.83 (0.57-1.11)	5,389 (3,989 - 6,916)	159 (114 - 208)	1.33 (0.97-1.7)
Sub-Saharan Africa	1,517 (760 - 2,564)	22 (12 - 34)	0.32 (0.18-0.49)	2,058 (1,160 - 3,296)	37 (24 - 52)	0.48 (0.32-0.67)
**Low temperature**						
Global	22,191 (19,689 - 25,193)	1,021 (938 - 1,104)	2.19 (2.01-2.35)	21,240 (19,042 - 23,435)	1,337 (1,187 - 1,456)	2.37 (2.12-2.57)
Southeast Asia, East Asia, and Oceania	8,510 (7,161 - 9,737)	360 (315 - 401)	2.99 (2.67-3.26)	7,565 (6,392 - 8,762)	484 (413 - 552)	3.11 (2.82-3.4)
Central Europe, Eastern Europe, and Central Asia	3,340 (2,815 - 3,882)	180 (154 - 205)	4.18 (3.56-4.76)	3,162 (2,608 - 3,706)	194 (164 - 223)	4.1 (3.46-4.66)
High-income	4,220 (3,832 - 4,583)	296 (267 - 321)	3.85 (3.47-4.17)	4,178 (3,679 - 4,562)	342 (292 - 377)	3.44 (2.94-3.79)
Latin America and Caribbean	709 (605 - 815)	27 (24 - 29)	1.12 (1.02-1.23)	631 (538 - 726)	42 (37 - 48)	1.18 (1.04-1.3)
North Africa and Middle East	1,986 (1,701 - 2,423)	62 (56 - 69)	2.42 (2.22-2.68)	1,965 (1,702 - 2,233)	99 (87 - 111)	3.2 (2.91-3.46)
South Asia	1,788 (739 - 3,015)	62 (46 - 81)	0.58 (0.42-0.76)	2,915 (2,090 - 3,777)	143 (114 - 176)	1.2 (0.97-1.44)
Sub-Saharan Africa	1,638 (1,215 - 2,366)	34 (28 - 43)	0.5 (0.41-0.62)	825 (627 - 1,144)	32 (28 - 38)	0.42 (0.36-0.5)
**Non-optimal temperature**						
Global	33,297 (29,683 - 37,296)	1,224 (1,132 - 1,312)	2.62 (2.43-2.8)	32,753 (29,567 - 36,062)	1,686 (1,515 - 1,826)	2.98 (2.7-3.2)
Southeast Asia, East Asia, and Oceania	10,838 (9,387 - 12,246)	409 (360 - 451)	3.39 (3.06-3.65)	9,567 (8,296 - 10,882)	565 (488 - 638)	3.63 (3.32-3.92)
Central Europe, Eastern Europe, and Central Asia	3,510 (2,998 - 4,048)	185 (159 - 210)	4.29 (3.68-4.85)	3,394 (2,863 - 3,930)	204 (174 - 233)	4.3 (3.67-4.85)
High-income	4,466 (4,081 - 4,830)	307 (277 - 331)	3.99 (3.6-4.3)	4,475 (3,985 - 4,833)	359 (307 - 393)	3.61 (3.1-3.95)
Latin America and Caribbean	1,002 (887 - 1,142)	32 (29 - 35)	1.34 (1.24-1.44)	1,110 (978 - 1,265)	56 (50 - 62)	1.55 (1.4-1.68)
North Africa and Middle East	3,167 (2,751 - 3,796)	84 (77 - 94)	3.32 (3.06-3.62)	3,073 (2,637 - 3,531)	134 (118 - 150)	4.32 (3.95-4.66)
South Asia	7,161 (4,983 - 9,591)	152 (120 - 185)	1.4 (1.1-1.71)	8,251 (6,553 - 10,104)	299 (243 - 360)	2.51 (2.07-2.94)
Sub-Saharan Africa	3,153 (2,297 - 4,387)	56 (46 - 70)	0.82 (0.67-1.03)	2,883 (1,984 - 4,060)	69 (56 - 85)	0.91 (0.75-1.07)

Table S6: Burden estimates for high temperature, low temperature and non-optimal temperature exposure by cause in 1990 and 2019 (95%UI displayed in parentheses)

	1990			2019		
Cause	Attributable DALYs (thousands)	Attributable Deaths (thousands)	PAFs (% deaths)	Attributable DALYs (thousands)	Attributable Deaths (thousands)	PAFs (% deaths)
**High temperature**						
All causes	11,113 (8,067 - 14,356)	205 (163 - 248)	0.44 (0.35-0.53)	11,630 (9,300 - 14,228)	356 (289 - 430)	0.63 (0.51-0.75)
Lower respiratory infections	5,323 (2,576 - 8,253)	71 (35 - 108)	1.5 (0.76-2.32)	3,183 (1,547 - 5,004)	64 (32 - 98)	2.26 (1.15-3.41)
Ischaemic heart disease	551 (200 - 882)	24 (10 - 38)	0.61 (0.16-1)	1,997 (1,238 - 2,846)	92 (58 - 131)	1.5 (0.9-2.14)
Stroke	537 (226 - 840)	22 (9 - 35)	0.63 (0.24-1.02)	1,288 (673 - 1,941)	60 (31 - 90)	1.38 (0.69-2.06)
Hypertensive heart disease	18 (-36 - 67)	1 (-1 - 3)	0.14 (-0.28-0.51)	81 (-11 - 177)	4 (0 - 9)	0.46 (-0.22-1.18)
Cardiomyopathy and myocarditis	25 (-6 - 59)	1 (0 - 2)	0.36 (-0.11-0.83)	49 (-1 - 101)	2 (0 - 4)	0.55 (-0.14-1.29)
Chronic obstructive pulmonary disease	127 (-112 - 366)	7 (-4 - 18)	0.23 (-0.31-0.72)	315 (-174 - 815)	18 (-8 - 45)	0.65 (-0.33-1.56)
Diabetes mellitus	193 (108 - 280)	7 (4 - 11)	1.19 (0.62-1.75)	734 (425 - 1,014)	32 (19 - 44)	2.21 (1.26-3.04)
Chronic kidney disease	71 (-39 - 182)	2 (-1 - 6)	0.38 (-0.28-1)	295 (15 - 575)	11 (1 - 21)	1 (-0.06-2.05)
Road injuries	989 (635 - 1,401)	17 (11 - 24)	1.53 (1.02-2.14)	1,283 (772 - 1,861)	26 (16 - 38)	2.27 (1.45-3.21)
Other transport injuries	50 (-3 - 102)	1 (0 - 2)	1.18 (-0.1-2.38)	55 (-20 - 127)	1 (0 - 3)	1.44 (-0.58-3.32)
Drowning	1,376 (782 - 2,044)	19 (11 - 27)	3.1 (2-4.26)	554 (293 - 842)	10 (5 - 14)	4 (2.33-5.76)
Exposure to mechanical forces	124 (44 - 223)	2 (1 - 4)	1.58 (0.64-2.65)	126 (36 - 231)	2 (1 - 4)	1.98 (0.66-3.33)
Animal contact	211 (-2 - 480)	3 (0 - 8)	3.39 (0.04-6.91)	163 (-25 - 377)	3 (-1 - 8)	3.66 (-0.52-7.73)
Exposure to forces of nature	301 (210 - 406)	5 (3 - 7)	10.1 (6.94-13.37)	11 (0 - 22)	0 (0 - 0)	3.36 (-0.03-6.89)
Other unintentional injuries	176 (59 - 311)	3 (1 - 5)	2.37 (1.01-3.87)	153 (29 - 284)	3 (1 - 5)	2.89 (0.72-4.97)
Self-harm	619 (356 - 880)	12 (7 - 17)	1.74 (1.01-2.51)	761 (352 - 1,186)	16 (8 - 25)	2.37 (1.06-3.58)
Interpersonal violence	419 (314 - 558)	7 (5 - 10)	1.92 (1.44-2.51)	581 (435 - 741)	11 (8 - 13)	2.46 (1.89-3.11)
**Low temperature**						
All causes	22,191 (19,689 - 25,193)	1,021 (938 - 1,104)	2.19 (2.01-2.35)	21,240 (19,042 - 23,435)	1,337 (1,187 - 1,456)	2.37 (2.12-2.57)
Lower respiratory infections	12,546 (10,395 - 15,471)	212 (183 - 250)	6.25 (5.52-7.09)	3,796 (3,052 - 4,763)	146 (124 - 168)	4.5 (3.8-5.28)
Ischaemic heart disease	6,785 (5,986 - 7,653)	337 (296 - 379)	5.15 (4.59-5.77)	9,109 (7,986 - 10,303)	493 (426 - 561)	4.56 (3.99-5.2)
Stroke	5,915 (5,241 - 6,617)	292 (257 - 327)	4.97 (4.41-5.54)	6,336 (5,498 - 7,214)	359 (308 - 409)	3.85 (3.33-4.43)
Hypertensive heart disease	905 (713 - 1,061)	47 (37 - 54)	6.62 (5.73-7.54)	1,179 (861 - 1,368)	74 (53 - 87)	4.86 (4.2-5.58)
Cardiomyopathy and myocarditis	396 (296 - 520)	15 (12 - 19)	5.52 (3.71-7.28)	429 (271 - 600)	18 (13 - 24)	4.53 (2.55-6.54)
Chronic obstructive pulmonary disease	4,396 (3,526 - 4,972)	234 (192 - 263)	7.65 (6.7-8.57)	4,007 (3,426 - 4,609)	255 (218 - 292)	5.19 (4.49-5.92)
Diabetes mellitus	667 (551 - 782)	29 (24 - 34)	3.46 (2.78-4.12)	1,148 (907 - 1,377)	56 (44 - 66)	3.01 (2.37-3.65)

Chronic kidney disease	819<br>(698 - 940)	30<br>(26 - 35)	4.31<br>(3.62-5.02)	1,397<br>(1,177 - 1,620)	69<br>(58 - 79)	3.44<br>(2.79-4.11)
Road injuries	-1,694<br>(-2,085 - -1,319)	-32<br>(-39 - -25)	-3.03<br>(-3.71- -2.39)	-1,352<br>(-1,713 - -1,036)	-30<br>(-37 - -23)	-2.48<br>(-3.06- -1.92)
Other transport injuries	-252<br>(-316 - -189)	-5<br>(-6 - -4)	-6.04<br>(-7.56- -4.44)	-174<br>(-225 - -121)	-4<br>(-5 - -3)	-4.3<br>(-5.75- -2.85)
Drowning	-4,248<br>(-4,838 - -3,632)	-60<br>(-69 - -52)	-13.43<br>(-15.29- -11.45)	-1,418<br>(-1,661 - -1,219)	-28<br>(-33 - -24)	-11.65<br>(-13.23- -10)
Exposure to mechanical forces	-389<br>(-505 - -295)	-7<br>(-9 - -5)	-6.04<br>(-7.6- -4.61)	-284<br>(-370 - -198)	-6<br>(-8 - -4)	-5<br>(-6.12- -3.8)
Animal contact	-347<br>(-515 - -189)	-6<br>(-9 - -3)	-5.72<br>(-8.03- -3.62)	-240<br>(-372 - -131)	-5<br>(-8 - -3)	-5.38<br>(-7.7- -3.2)
Exposure to forces of nature	-93<br>(-135 - -39)	-2<br>(-2 - -1)	-2.86<br>(-4- -1.32)	-22<br>(-31 - -13)	0<br>(-1 - 0)	-6.62<br>(-9.3- -3.76)
Other unintentional injuries	-712<br>(-841 - -608)	-13<br>(-15 - -11)	-10.82<br>(-13.32- -9.14)	-506<br>(-599 - -408)	-10<br>(-12 - -8)	-9.83<br>(-11.41- -8.48)
Self-harm	-1,656<br>(-2,112 - -1,205)	-36<br>(-46 - -26)	-4.66<br>(-6.06- -3.39)	-1,443<br>(-1,846 - -1,081)	-34<br>(-44 - -25)	-4.3<br>(-5.43- -3.27)
Interpersonal violence	-846<br>(-1,078 - -623)	-15<br>(-20 - -11)	-3.72<br>(-4.73- -2.75)	-721<br>(-897 - -551)	-14<br>(-18 - -10)	-2.93<br>(-3.66- -2.28)
**Non-optimal temperature**						
All causes	33,297<br>(29,683 - 37,296)	1,224<br>(1,132 - 1,312)	2.62<br>(2.43-2.8)	32,753<br>(29,567 - 36,062)	1,686<br>(1,515 - 1,826)	2.98<br>(2.7-3.2)
Lower respiratory infections	17,699<br>(14,345 - 21,760)	281<br>(238 - 332)	7.71<br>(6.89-8.69)	6,900<br>(5,271 - 8,824)	208<br>(174 - 245)	6.7<br>(5.59-7.95)
Ischaemic heart disease	7,303<br>(6,458 - 8,187)	359<br>(317 - 403)	5.73<br>(5.06-6.44)	11,011<br>(9,592 - 12,418)	580<br>(501 - 653)	5.99<br>(5.19-6.83)
Stroke	6,435<br>(5,685 - 7,178)	313<br>(278 - 350)	5.59<br>(4.92-6.22)	7,589<br>(6,576 - 8,678)	417<br>(361 - 473)	5.19<br>(4.4-5.96)
Hypertensive heart disease	922<br>(720 - 1,082)	47<br>(38 - 55)	6.75<br>(5.85-7.58)	1,255<br>(927 - 1,467)	79<br>(57 - 91)	5.3<br>(4.41-6.15)
Cardiomyopathy and myocarditis	419<br>(317 - 556)	16<br>(12 - 19)	5.84<br>(4.07-7.63)	474<br>(314 - 649)	20<br>(14 - 26)	5.03<br>(2.94-7.22)
Chronic obstructive pulmonary disease	4,513<br>(3,590 - 5,141)	240<br>(196 - 272)	7.86<br>(6.85-8.88)	4,305<br>(3,534 - 5,092)	272<br>(224 - 318)	5.82<br>(4.73-6.98)
Diabetes mellitus	854<br>(711 - 982)	36<br>(30 - 42)	4.62<br>(3.79-5.37)	1,862<br>(1,499 - 2,208)	87<br>(70 - 102)	5.16<br>(4.09-6.14)
Chronic kidney disease	887<br>(732 - 1,044)	33<br>(28 - 38)	4.67<br>(3.75-5.6)	1,682<br>(1,352 - 2,047)	80<br>(66 - 94)	4.4<br>(3.19-5.64)
Road injuries	-686<br>(-1,185 - -141)	-14<br>(-23 - -4)	-1.47<br>(-2.31- -0.59)	-45<br>(-560 - 509)	-3<br>(-14 - 8)	-0.16<br>(-1.12-0.83)
Other transport injuries	-199<br>(-266 - -132)	-4<br>(-5 - -3)	-4.8<br>(-6.32- -3.14)	-117<br>(-192 - -49)	-3<br>(-4 - -1)	-2.8<br>(-4.76--0.98)
Drowning	-2,729<br>(-3,477 - -1,992)	-40<br>(-50 - -30)	-10<br>(-12.24- -7.81)	-812<br>(-1,116 - -520)	-18<br>(-23 - -13)	-7.26<br>(-9.5 - -5.11)
Exposure to mechanical forces	-261<br>(-388 - -141)	-5<br>(-7 - -3)	-4.39<br>(-6.1 - -2.75)	-155<br>(-260 - -58)	-4<br>(-6 - -2)	-2.95<br>(-4.52 - -1.35)
Animal contact	-123<br>(-335 - 101)	-2<br>(-6 - 1)	-2.12<br>(-5.22-1.18)	-66<br>(-242 - 110)	-2<br>(-5 - 2)	-1.48<br>(-5.19-2.12)
Exposure to forces of nature	216<br>(144 - 293)	3<br>(2 - 5)	7.46<br>(4.9-10.08)	-11<br>(-19 - -3)	0<br>(0 - 0)	-3.01<br>(-5.46--0.57)
Other unintentional injuries	-520<br>(-670 - -370)	-9<br>(-12 - -7)	-8.22<br>(-11.13--5.84)	-338<br>(-480 - -196)	-7<br>(-10 - -4)	-6.66<br>(-9.19--4)
Self-harm	-1,016<br>(-1,538 - -565)	-23<br>(-34 - -14)	-2.86<br>(-4.42--1.5)	-656<br>(-1,168 - -172)	-17<br>(-29 - -7)	-1.85<br>(-3.42--0.39)
Interpersonal violence	-417<br>(-664 - -167)	-8<br>(-12 - -3)	-1.76<br>(-2.88--0.61)	-127<br>(-353 - 102)	-3<br>(-7 - 1)	-0.42<br>(-1.28-0.48)

Figure S25: Spatial distribution of the proportion of deaths due to lower respiratory infections attributable to high temperature (A), low temperature (B), and non-optimal temperature (C) exposure, 2019

A) High temperature PAFs for lower respiratory infections

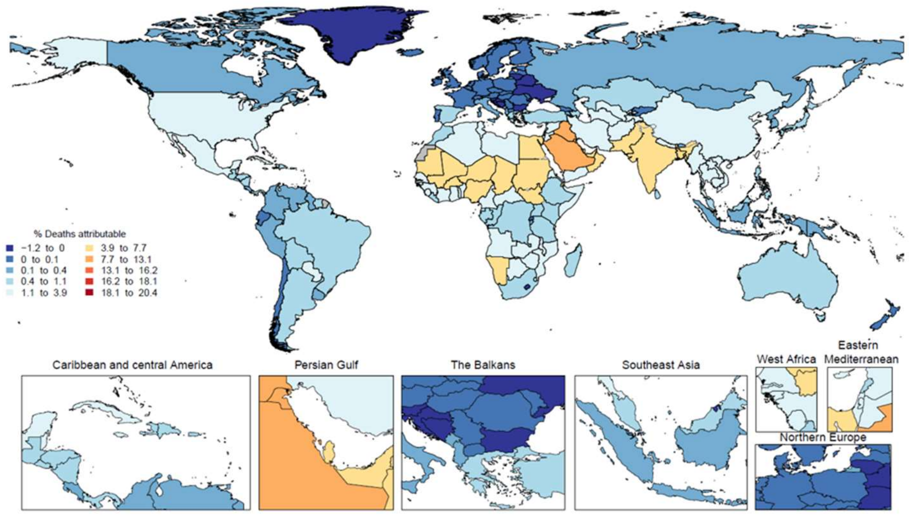

B) Low temperature PAFs for lower respiratory infections

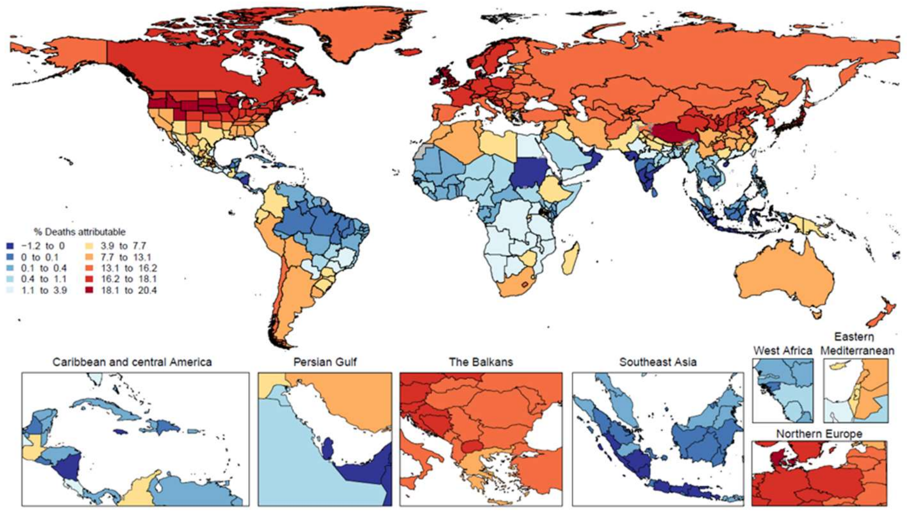

C) Non−optimal temperature PAFs for lower respiratory infections

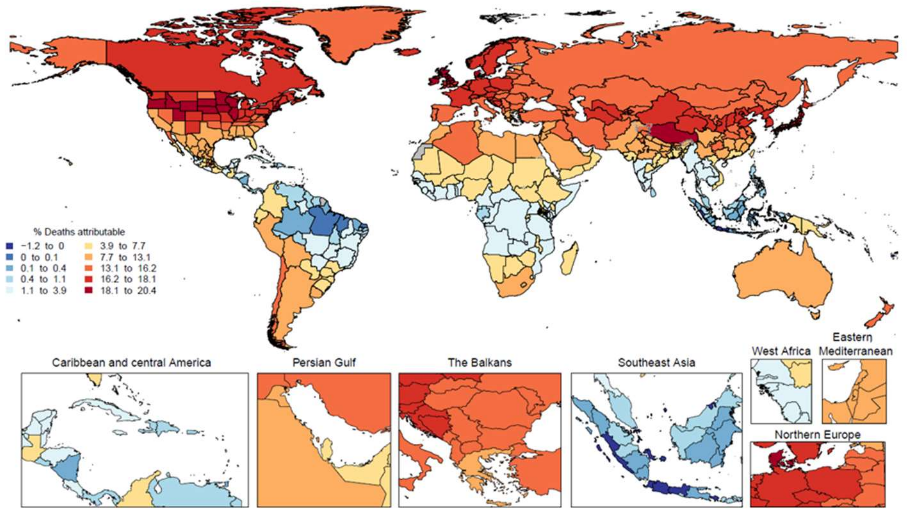

Figure S26: Spatial distribution of the proportion of deaths due to ischaemic heart disease attributable to high temperature (A), low temperature (B), and non-optimal temperature (C) exposure, 2019

A) High temperature PAFs for ischemic heart disease

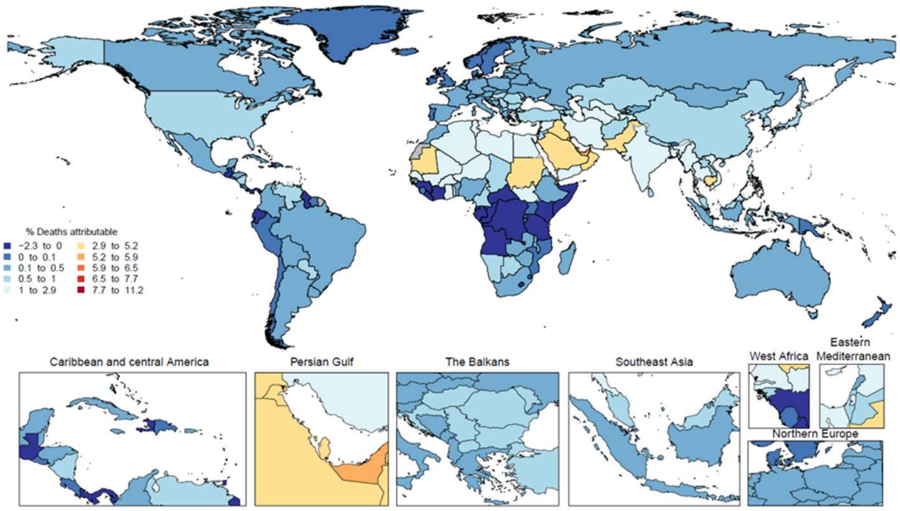

B) Low temperature PAFs for ischemic heart disease

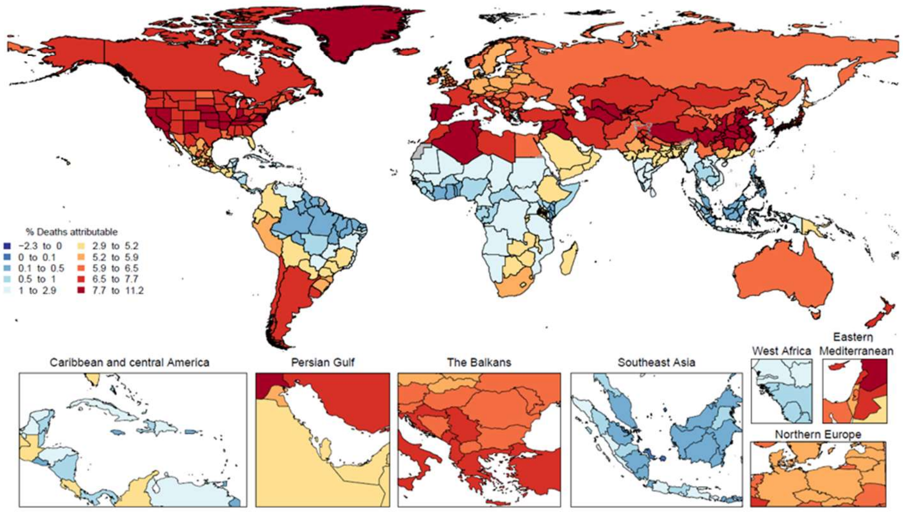

C) Non−optimal temperature PAFs for ischemic heart disease

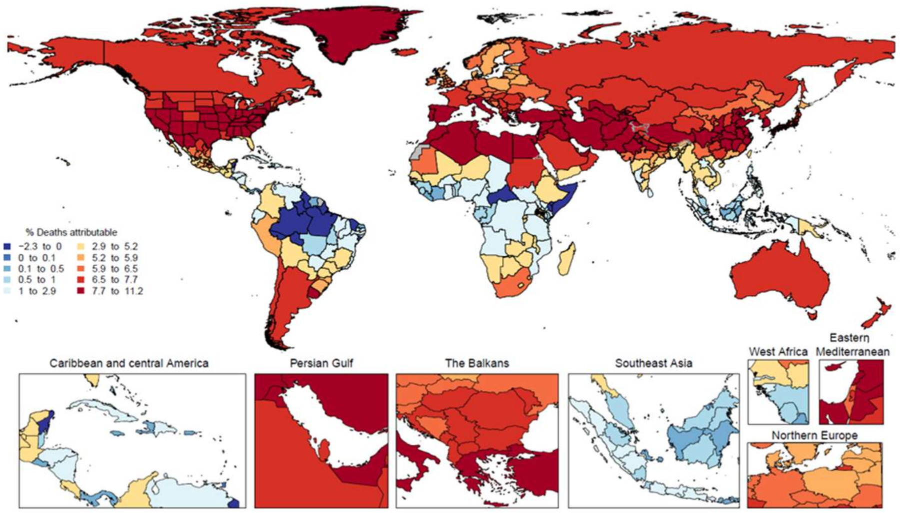

Figure S27: Spatial distribution of the proportion of deaths due to stroke attributable to high temperature (A), low temperature (B), and non-optimal temperature (C) exposure, 2019

A) High temperature PAFs for stroke

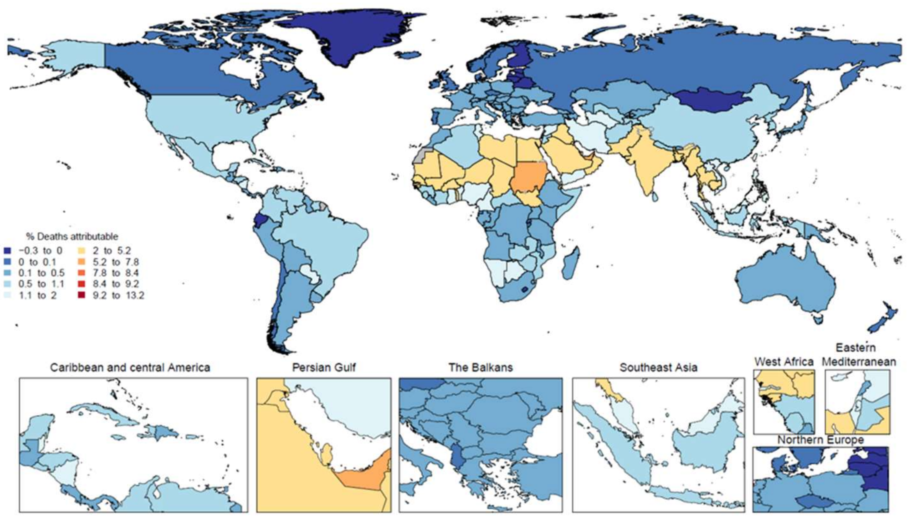

B) Low temperature PAFs for stroke

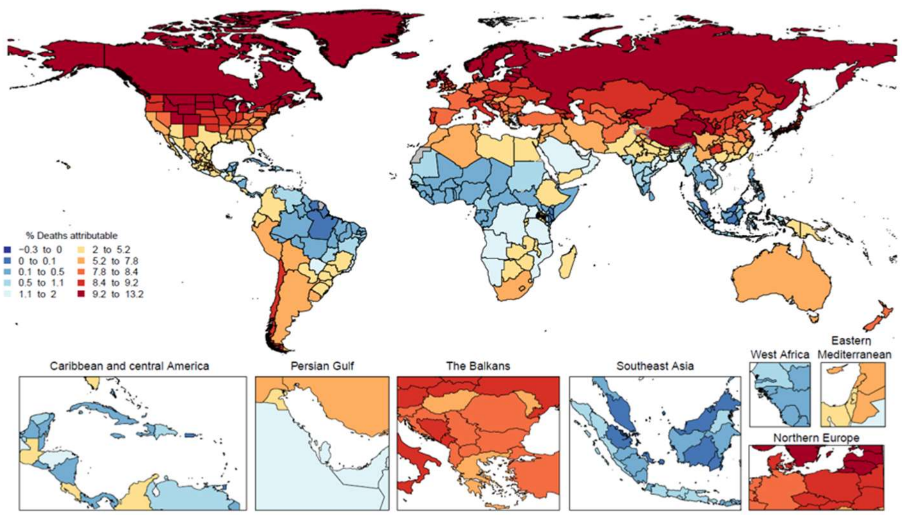

C) Non−optimal temperature PAFs for stroke

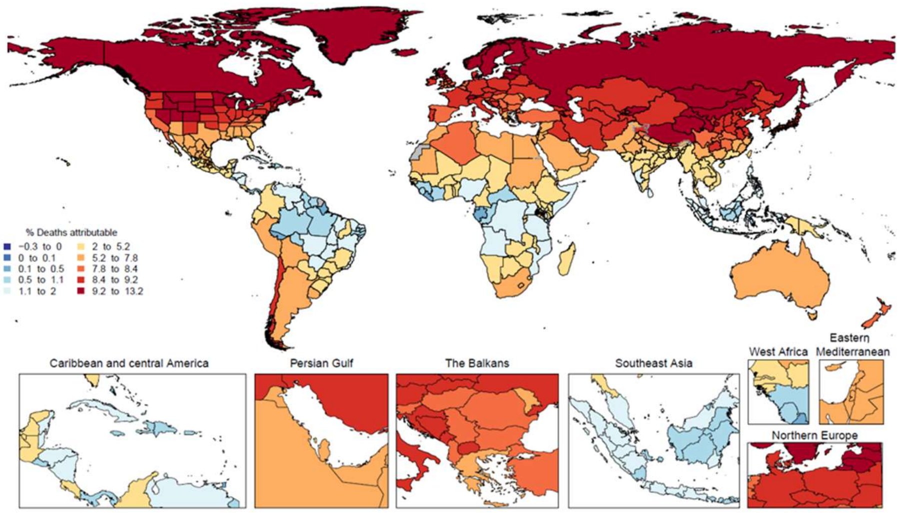

Figure S28: Spatial distribution of the proportion of deaths due to hypertensive heart disease attributable to high temperature (A), low temperature (B), and non-optimal temperature (C) exposure, 2019

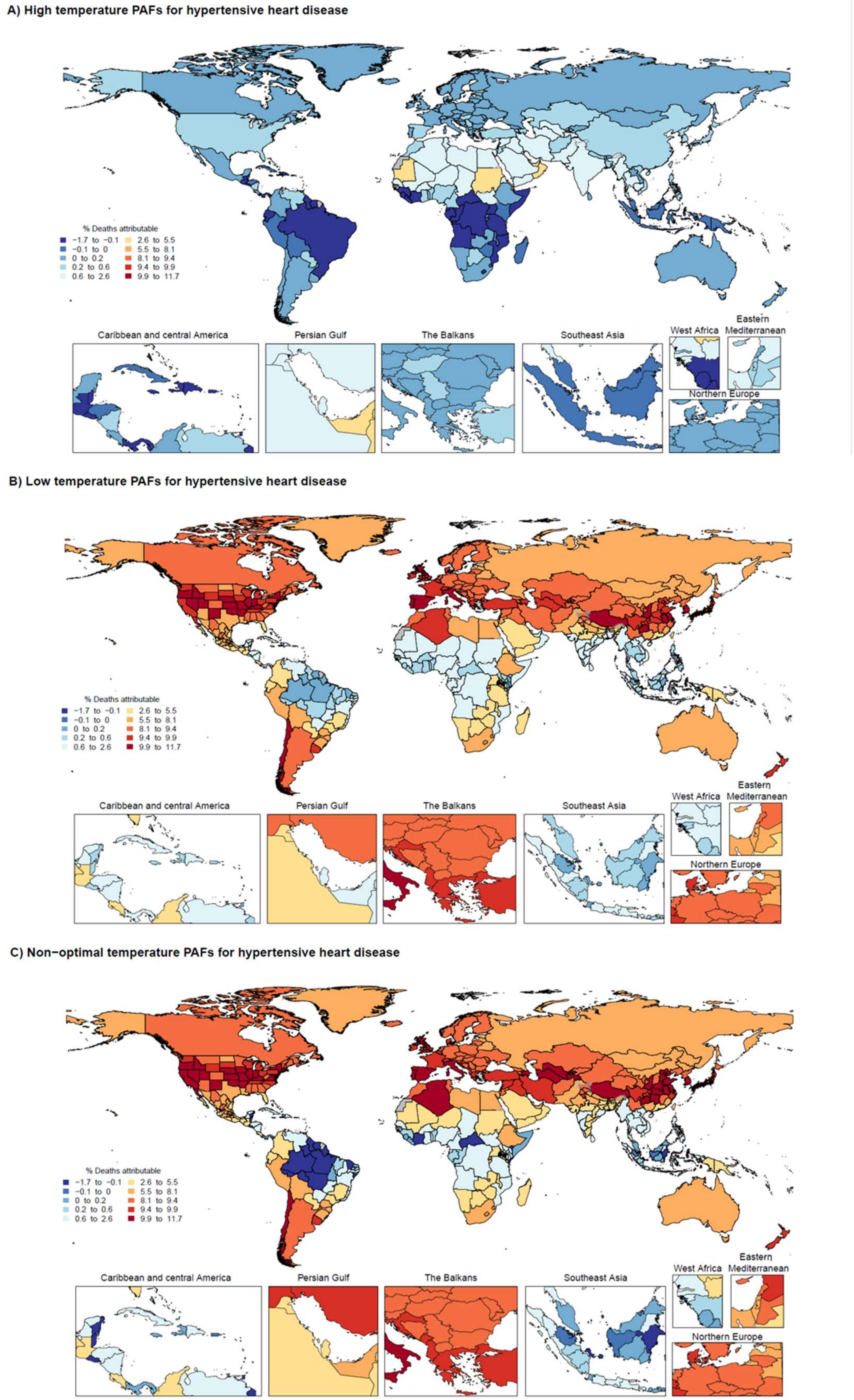

Figure S29: Spatial distribution of the proportion of deaths due to cardiomyopathy and myocarditis attributable to high temperature (A), low temperature (B), and non-optimal temperature (C) exposure, 2019

A) High temperature PAFs for cardiomyopathy and myocarditis

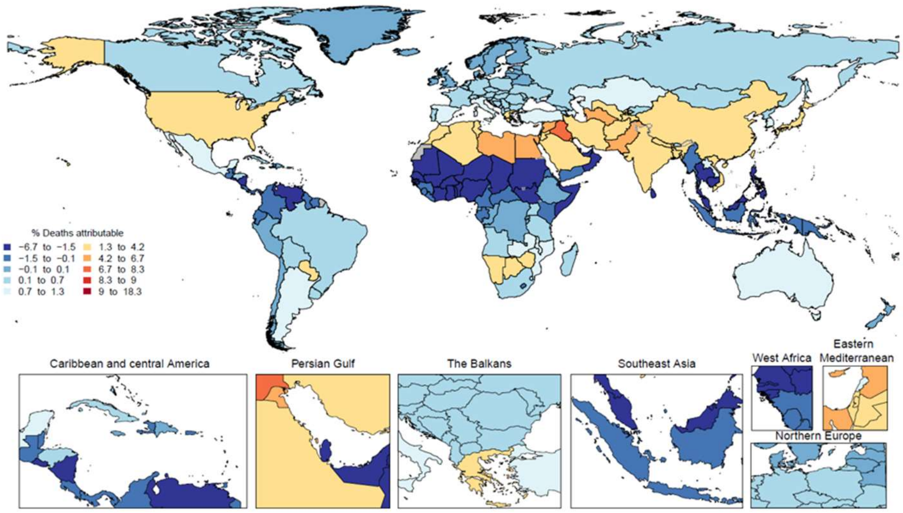

B) Low temperature PAFs for cardiomyopathy and myocarditis

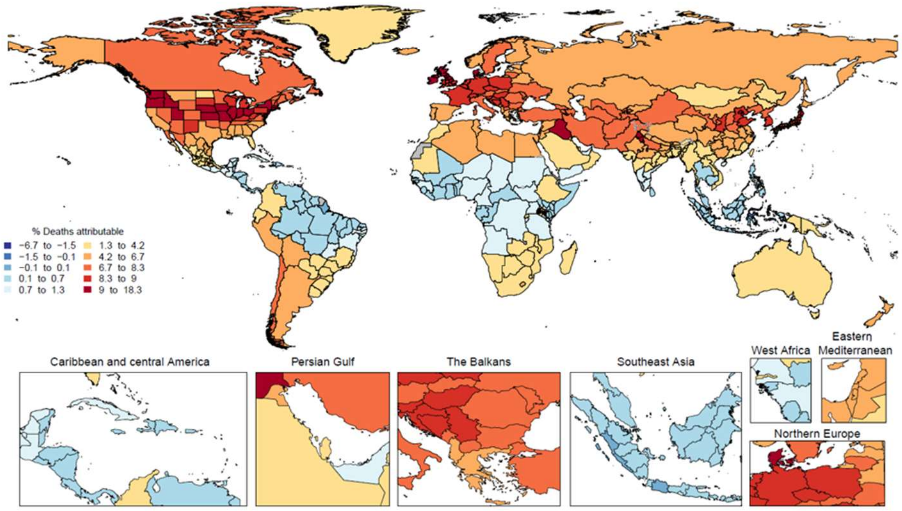

C) Non−optimal temperature PAFs for cardiomyopathy and myocarditis

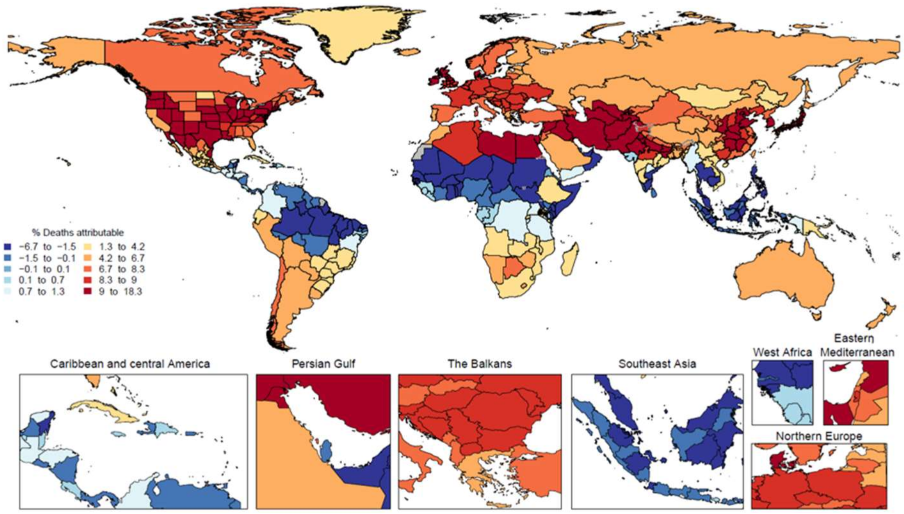

Figure S30: Spatial distribution of the proportion of deaths due to chronic obstructive pulmonary disease attributable to high temperature (A), low temperature (B), and non-optimal temperature (C) exposure, 2019

A) High temperature PAFs for chronic obstructive pulmonary disease

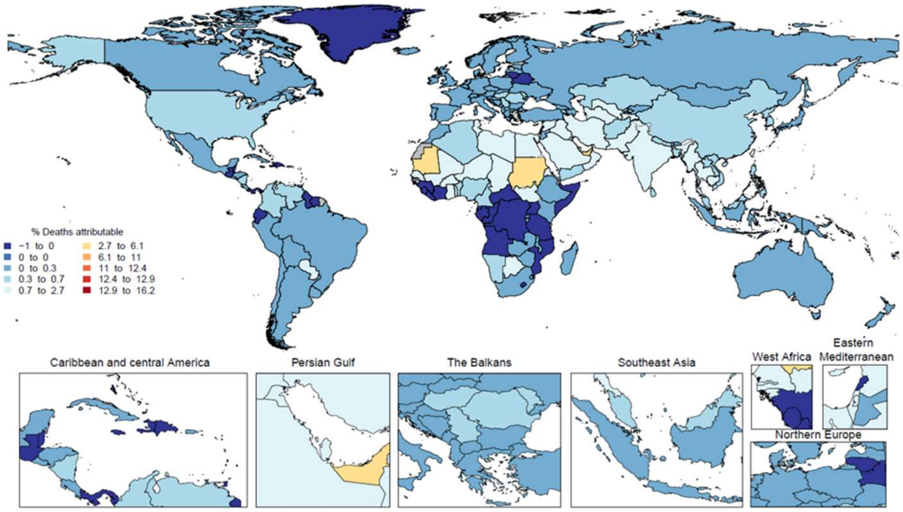

B) Low temperature PAFs for chronic obstructive pulmonary disease

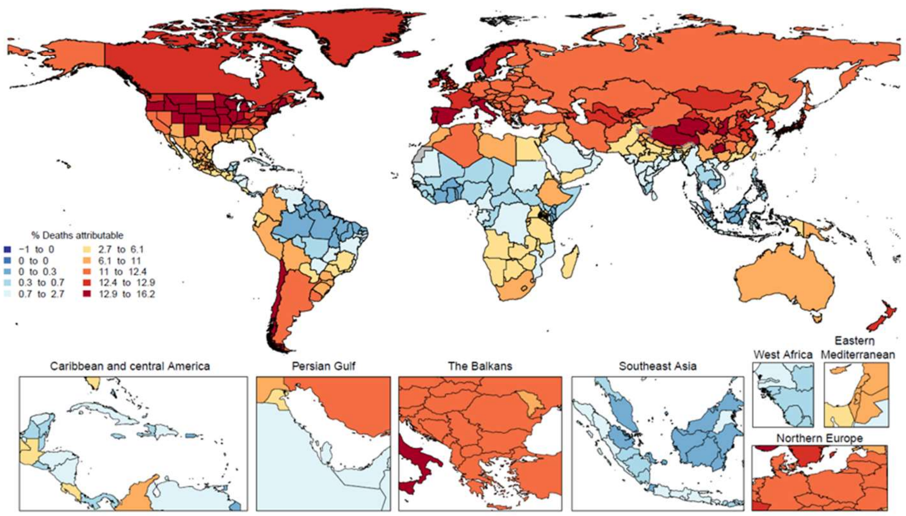

C) Non−optimal temperature PAFs for chronic obstructive pulmonary disease

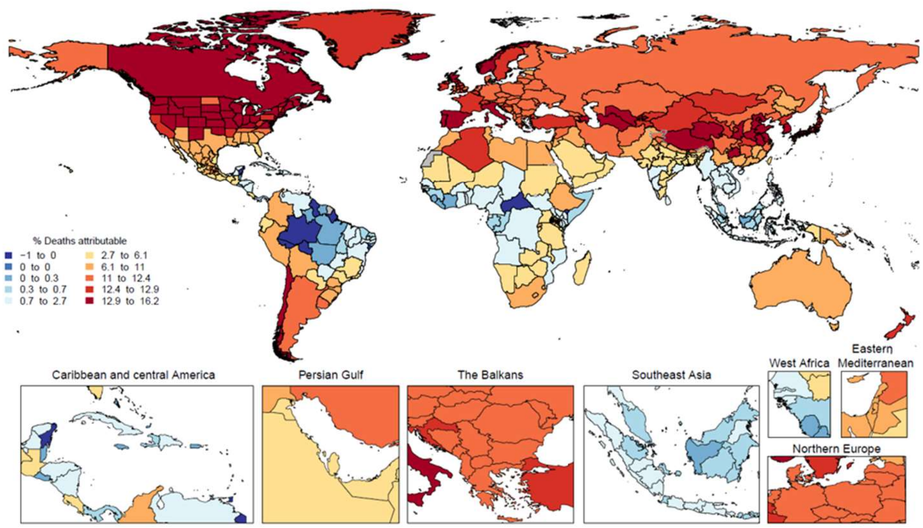

Figure S31: Spatial distribution of the proportion of deaths due to diabetes mellitus attributable to high temperature (A), low temperature (B), and non-optimal temperature (C) exposure, 2019

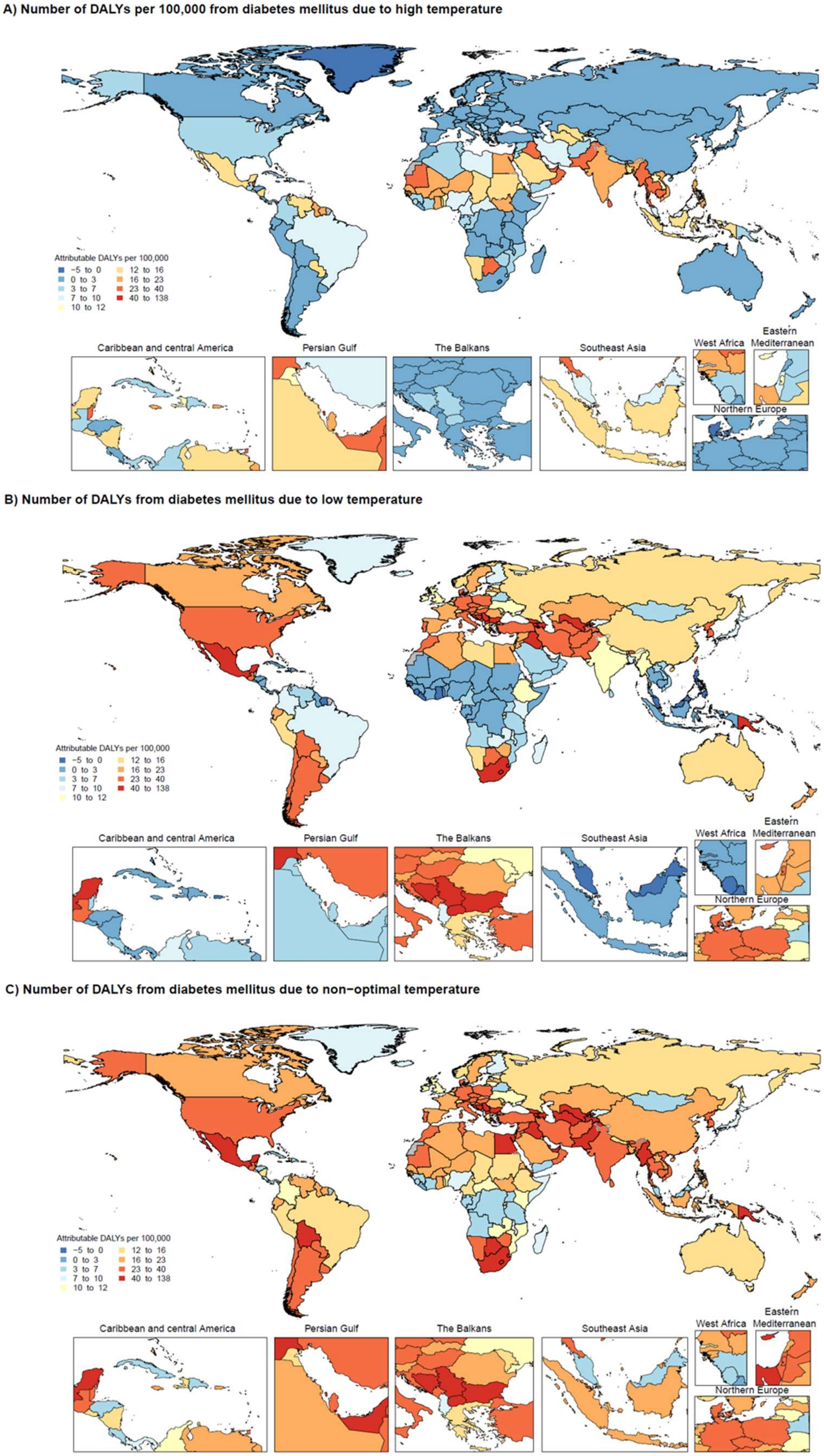

Figure S32: Spatial distribution of the proportion of deaths due to chronic kidney disease attributable to high temperature (A), low temperature (B), and non-optimal temperature (C) exposure, 2019

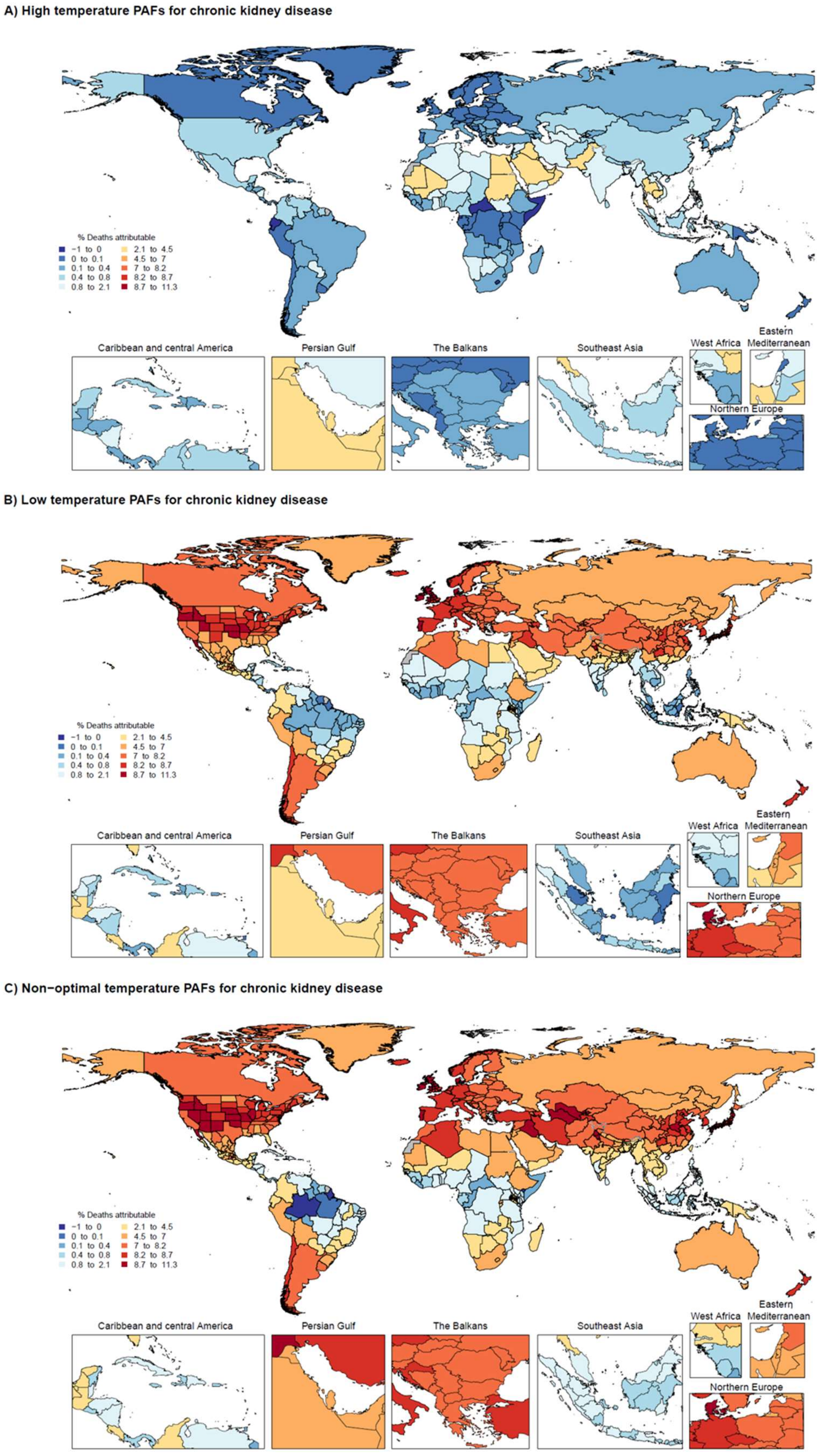

Figure S33: Spatial distribution of the proportion of deaths due to road injuries attributable to high temperature (A), low temperature (B), and non-optimal temperature (C) exposure, 2019

A) High temperature PAFs for road injuries

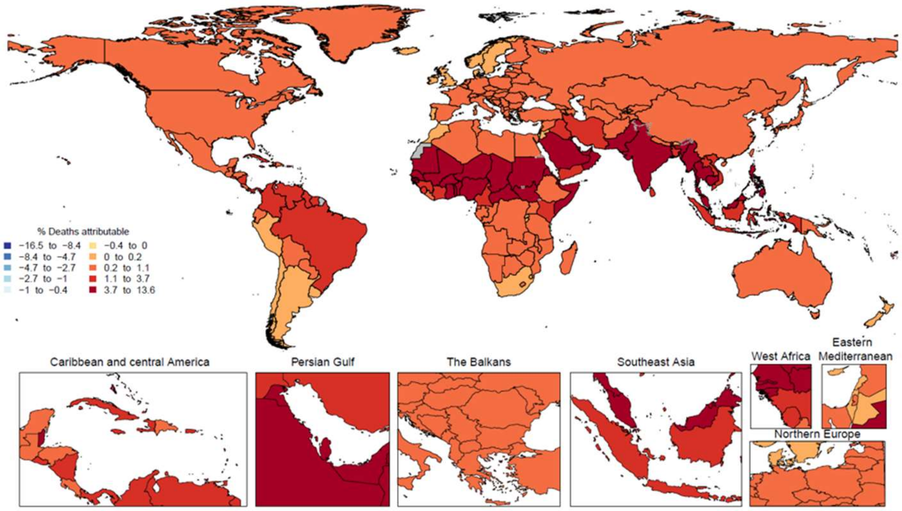

B) Low temperature PAFs for road injuries

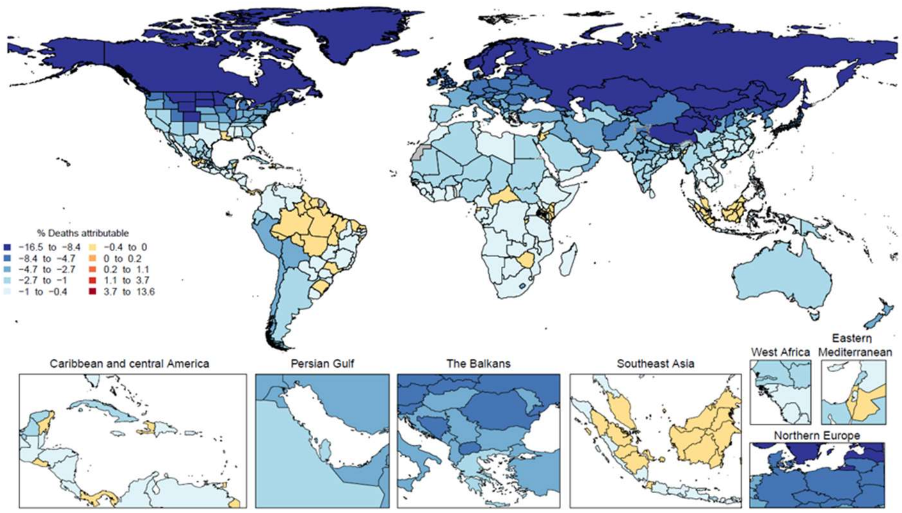

C) Non−optimal temperature PAFs for road injuries

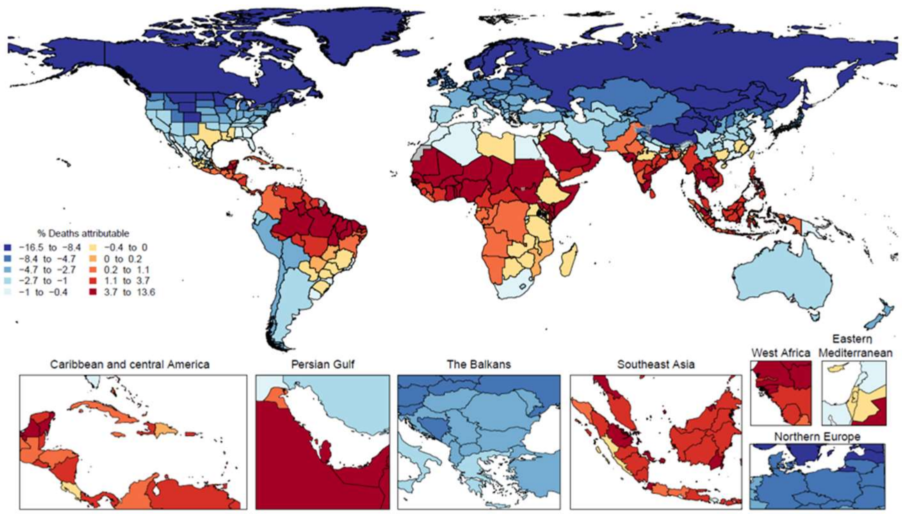

Figure S34: Spatial distribution of the proportion of deaths due to other transport-related injuries attributable to high temperature (A), low temperature (B), and non-optimal temperature (C) exposure, 2019

A) High temperature PAFs for other transport injuries

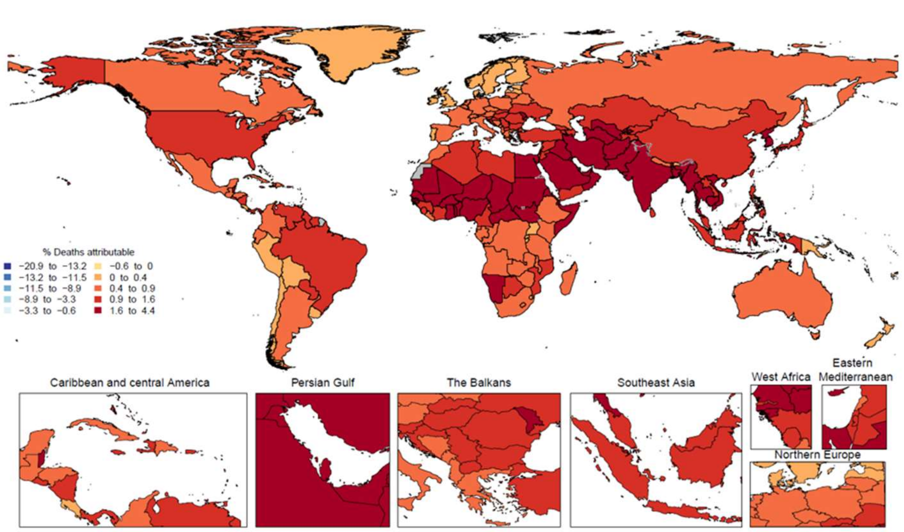

B) Low temperature PAFs for other transport injuries

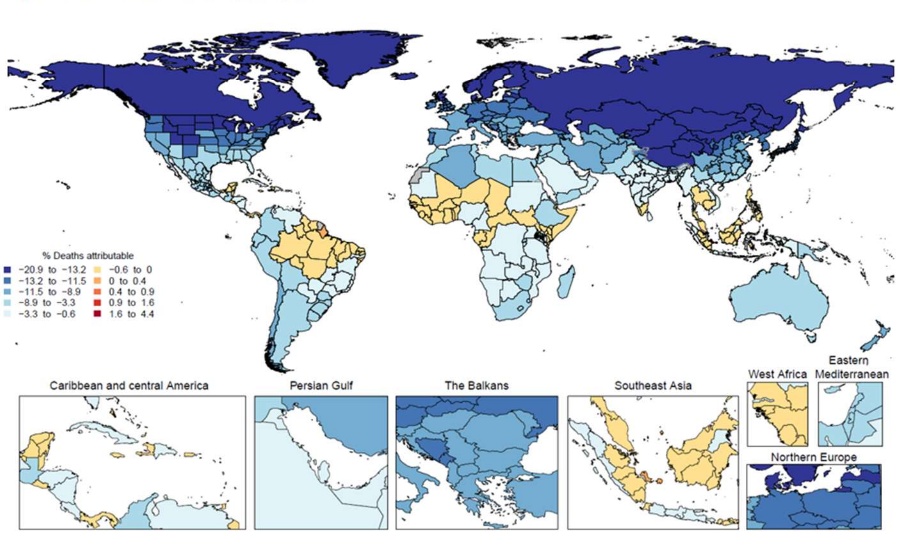

C) Non−optimal temperature PAFs for other transport injuries

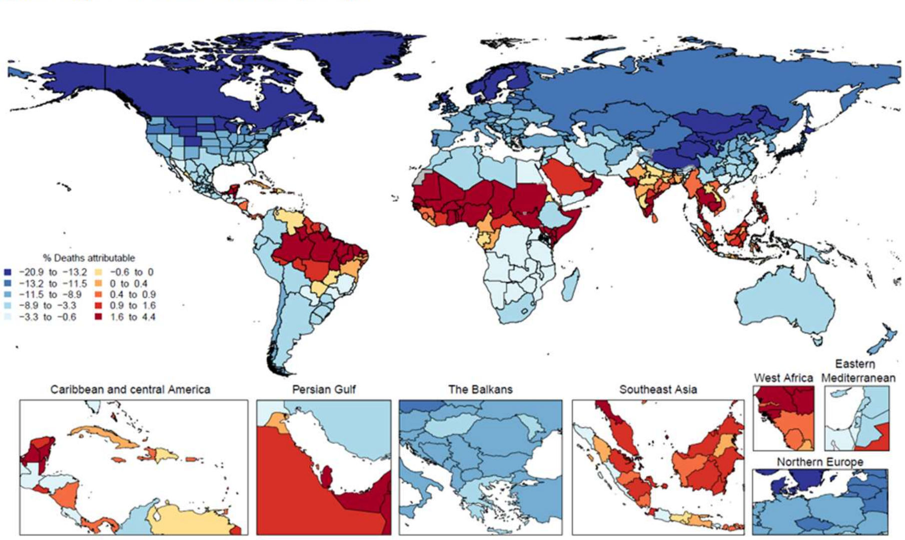

Figure S35: Spatial distribution of the proportion of deaths due to drowning attributable to high temperature (A), low temperature (B), and non-optimal temperature (C) exposure, 2019

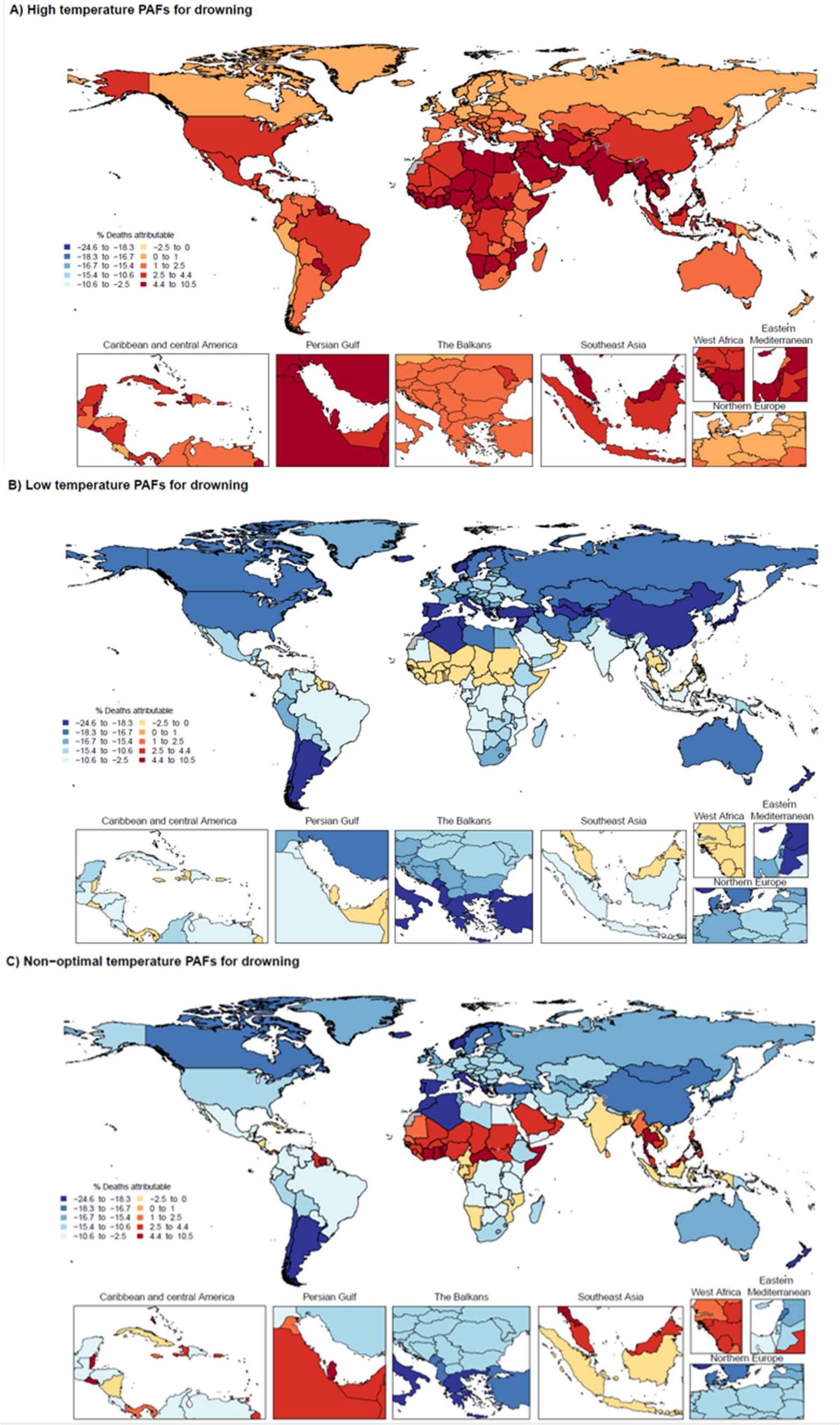

Figure S36: Spatial distribution of the proportion of deaths due to mechanical injuries (exposure to mechanical forces) attributable to high temperature (A), low temperature (B), and non-optimal temperature (C) exposure, 2019

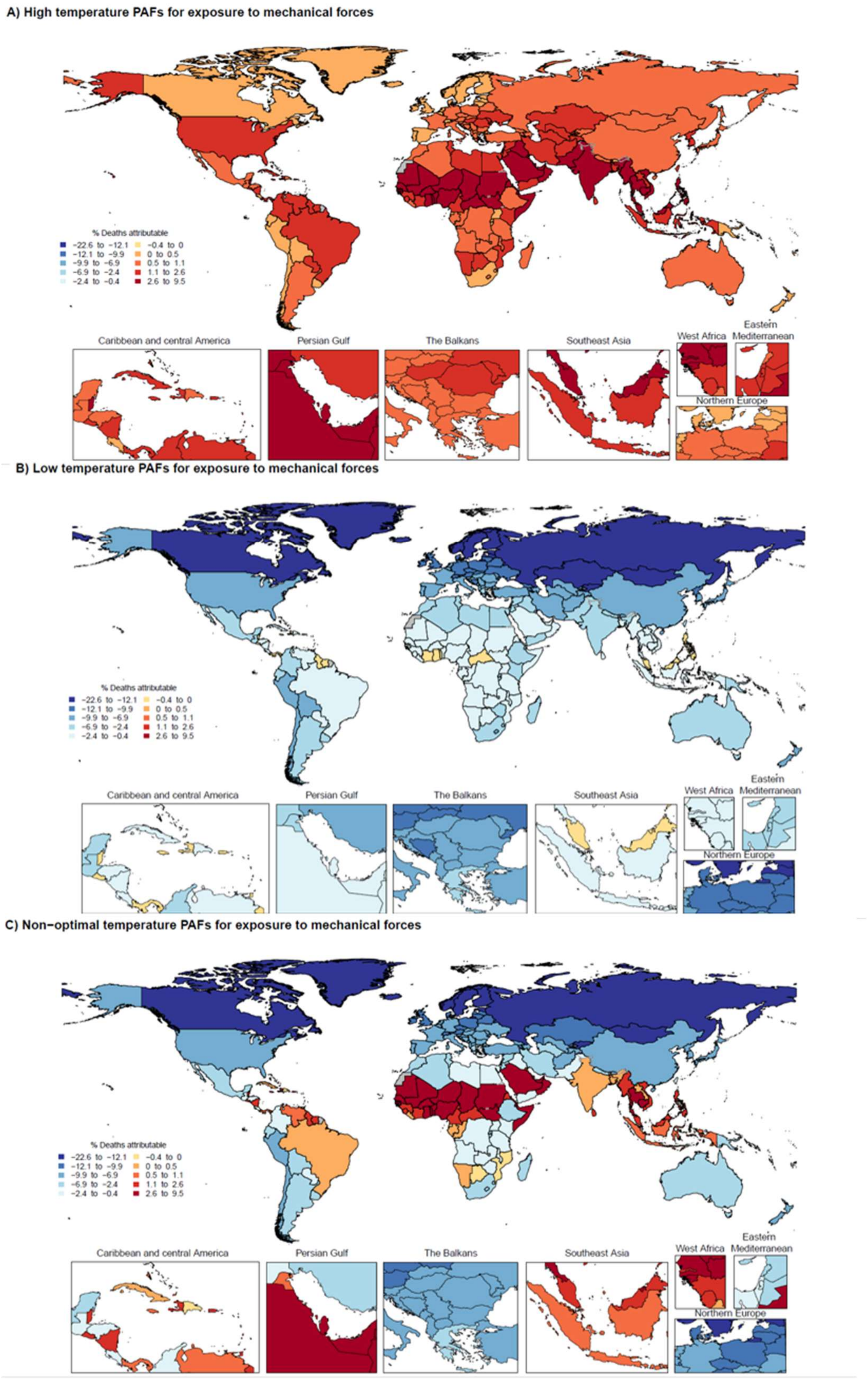

Figure S37: Spatial distribution of the proportion of deaths due to animal-related injuries (animal contact) attributable to high temperature (A), low temperature (B), and non-optimal temperature (C) exposure, 2019

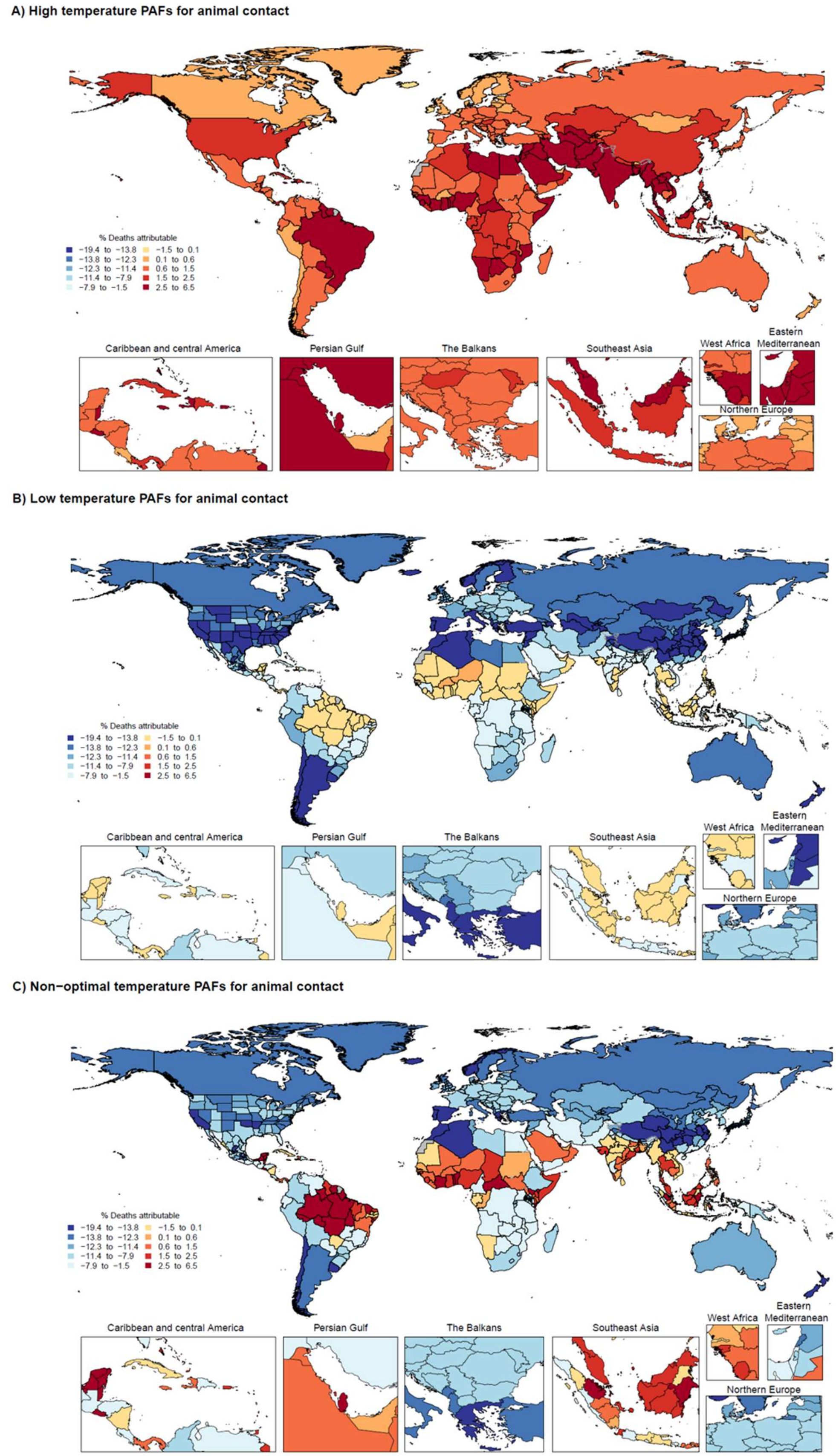

Figure S38: Spatial distribution of the proportion of deaths due to disaster-related injuries (exposure to forces of nature) attributable to high temperature (A), low temperature (B), and non-optimal temperature (C) exposure, 2019

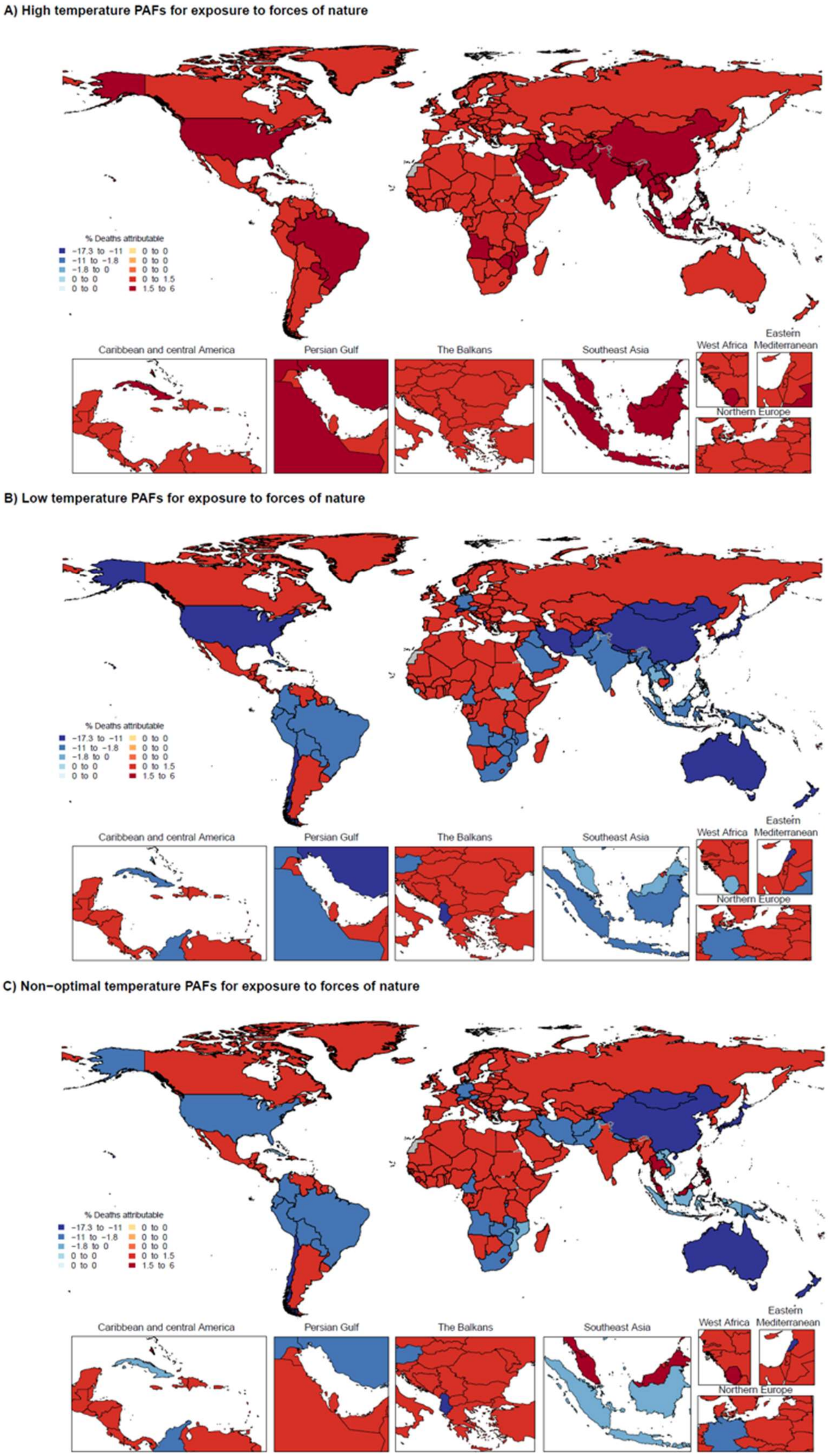

Figure S39: Spatial distribution of the proportion of deaths due to other unintentional injuries attributable to high temperature (A), low temperature (B), and non-optimal temperature (C) exposure, 2019

A) High temperature PAFs for other unintentional injuries

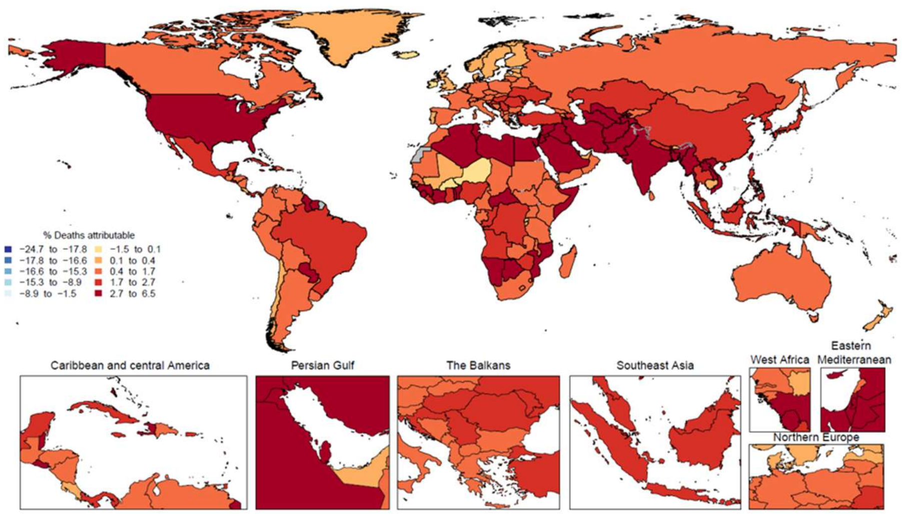

B) Low temperature PAFs for other unintentional injuries

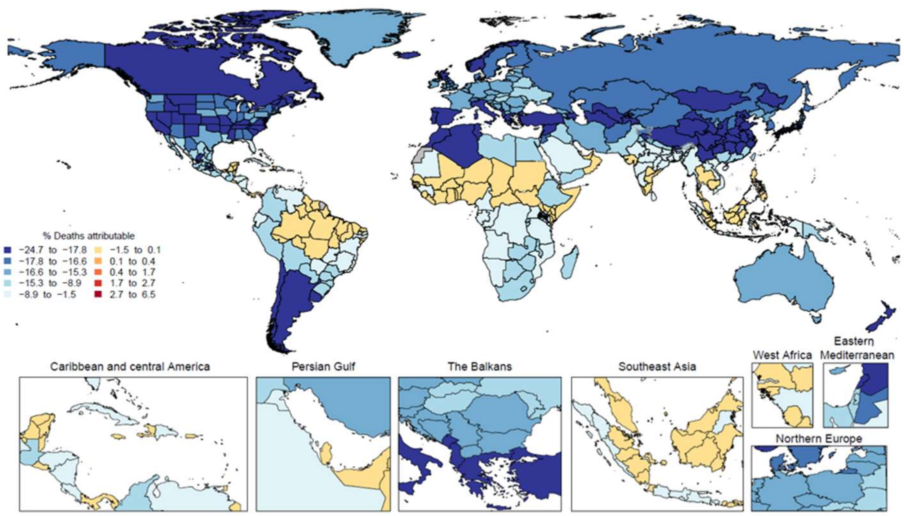

C) Non−optimal temperature PAFs for other unintentional injuries

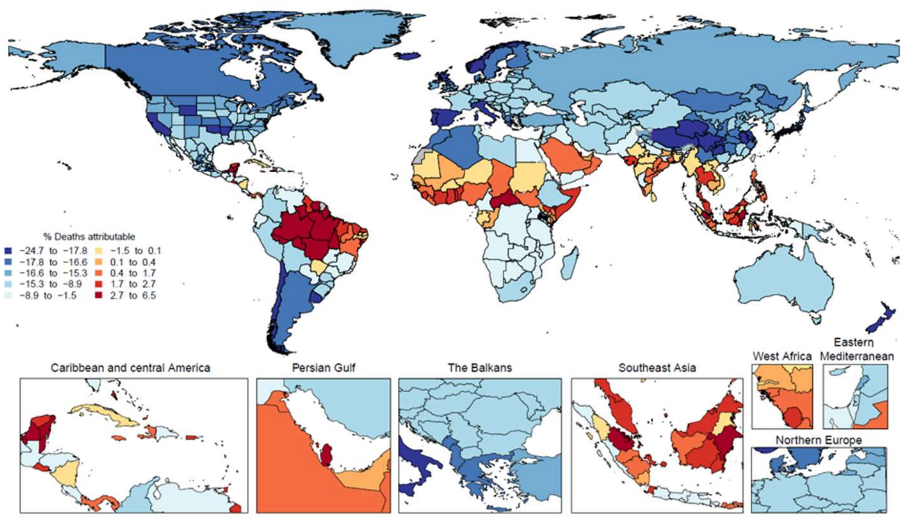

Figure S40: Spatial distribution of the proportion of deaths due to suicide (self-harm) attributable to high temperature (A), low temperature (B), and non-optimal temperature (C) exposure, 2019

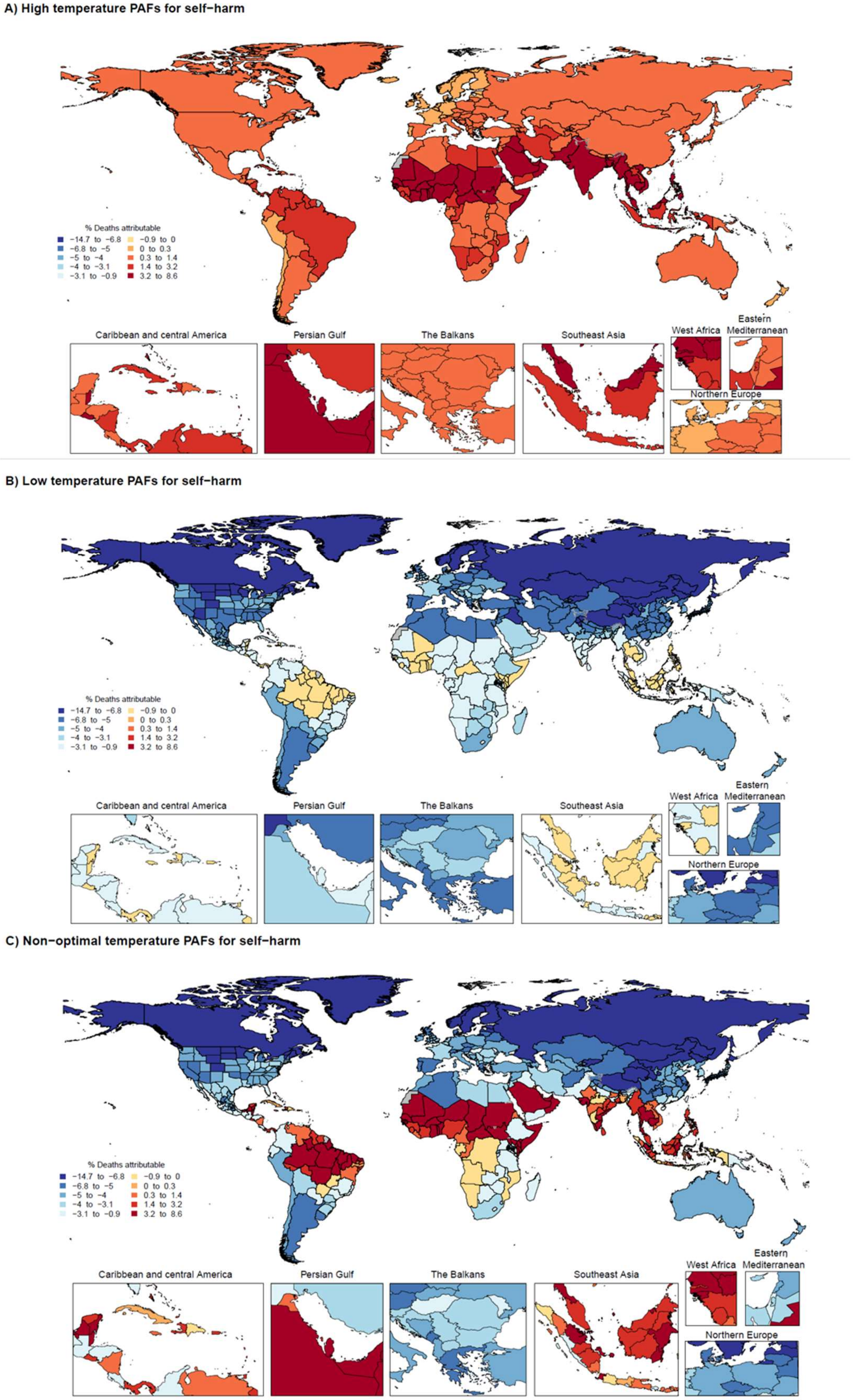

Figure S41: Spatial distribution of the proportion of deaths due to homicide (interpersonal violence) attributable to high temperature (A), low temperature (B), and non-optimal temperature (C) exposure, 2019

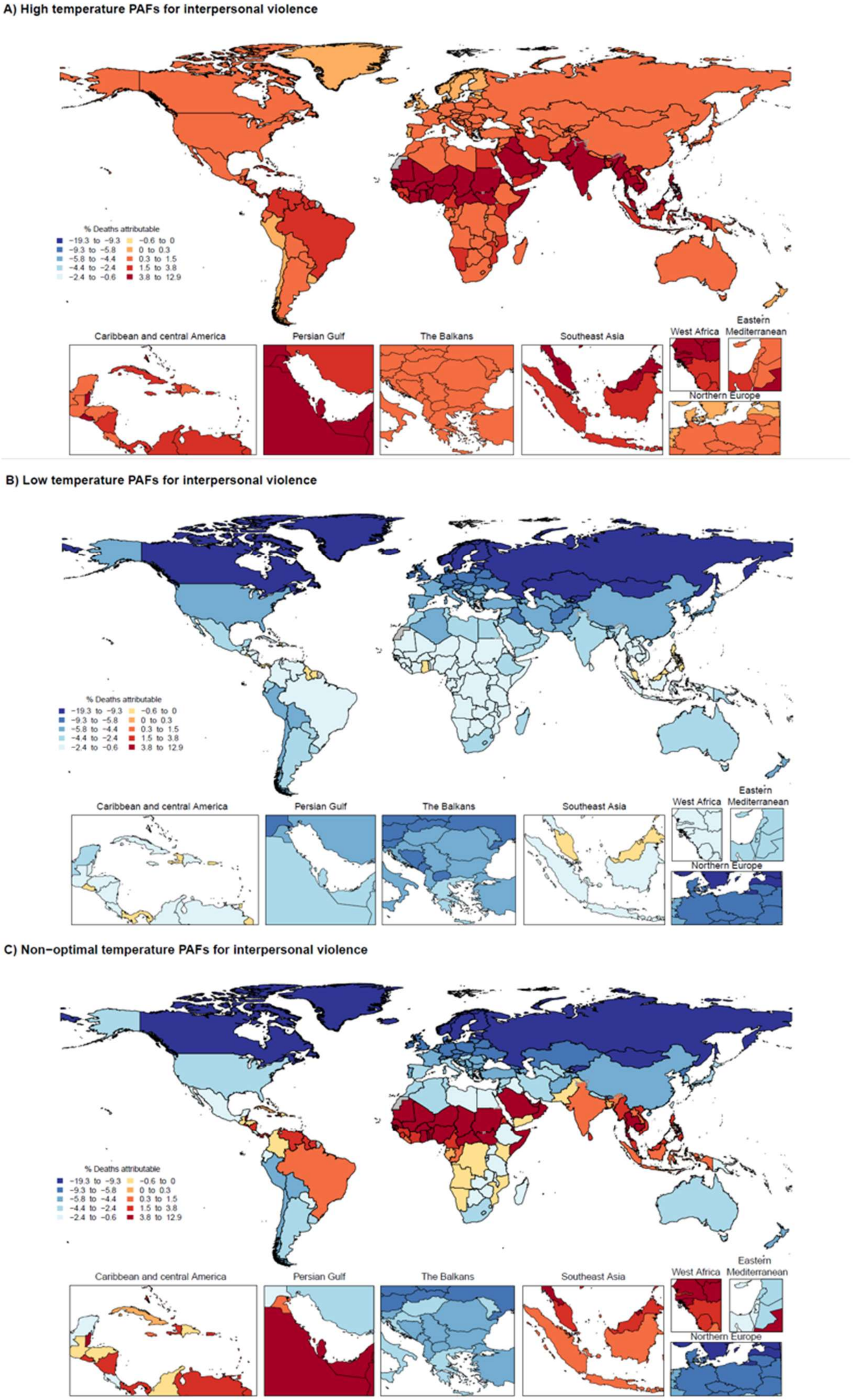

Figure S42: Spatial distribution of DALYs (per 100,000) due to lower respiratory infections attributable to high temperature (A), low temperature (B), and non-optimal temperature (C) exposure, 2019

A) Number of DALYs per 100,000 from lower respiratory infections due to high temperature

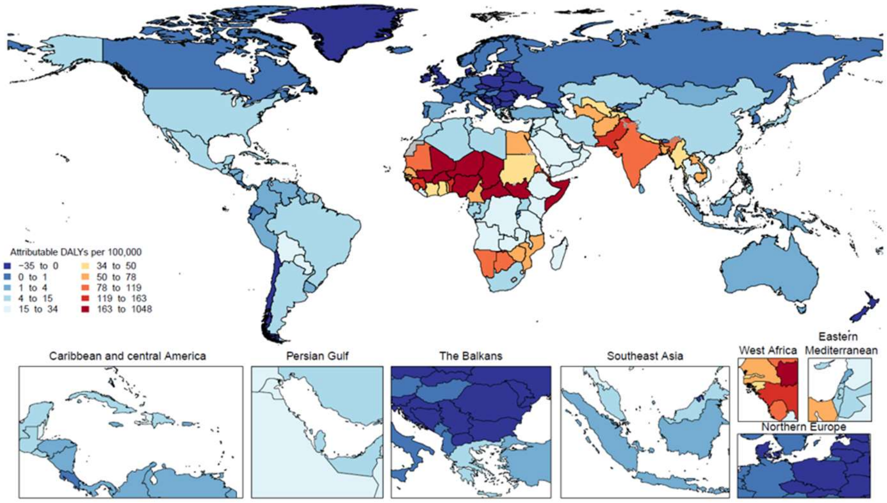

B) Number of DALYs from lower respiratory infections due to low temperature

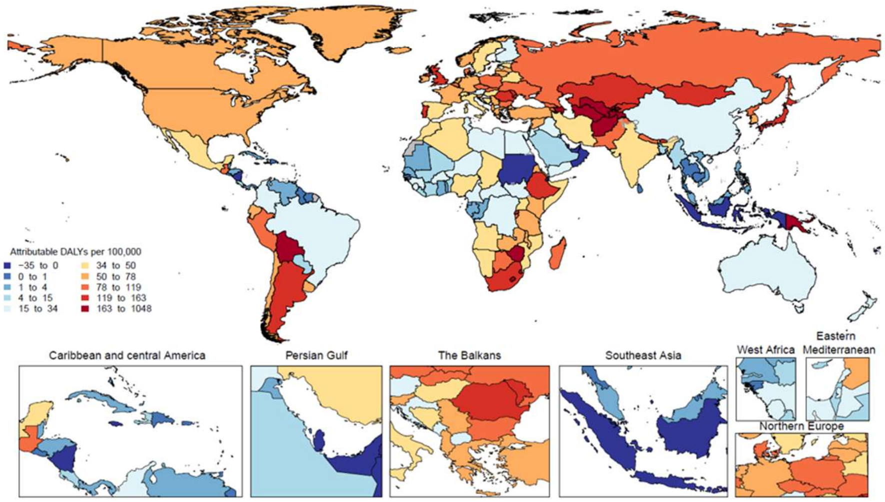

C) Number of DALYs from lower respiratory infections due to non−optimal temperature

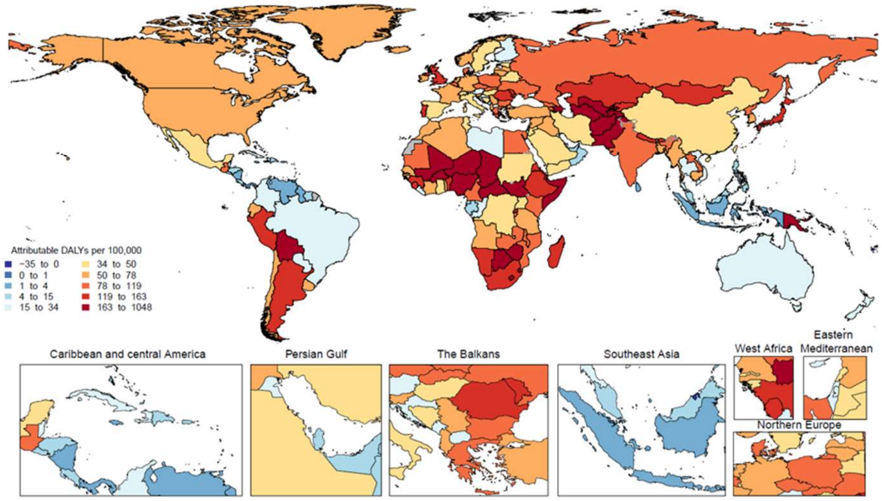

Figure S43: Spatial distribution of DALYs (per 100,000) due to ischaemic heart disease attributable to high temperature (A), low temperature (B), and non-optimal temperature (C) exposure, 2019

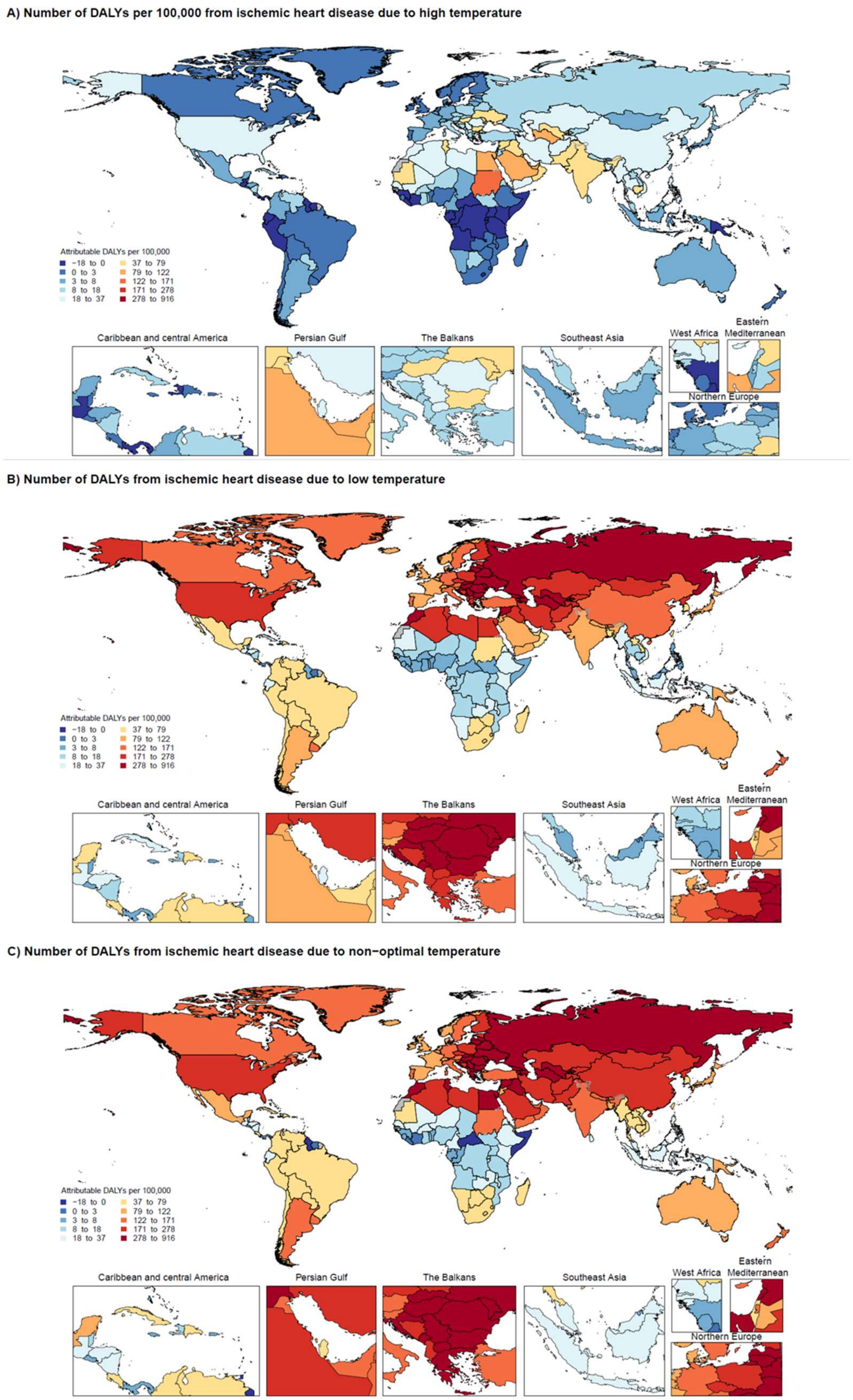

Figure S44: Spatial distribution of DALYs (per 100,000) due to stroke attributable to high temperature (A), low temperature (B), and non-optimal temperature (C) exposure, 2019

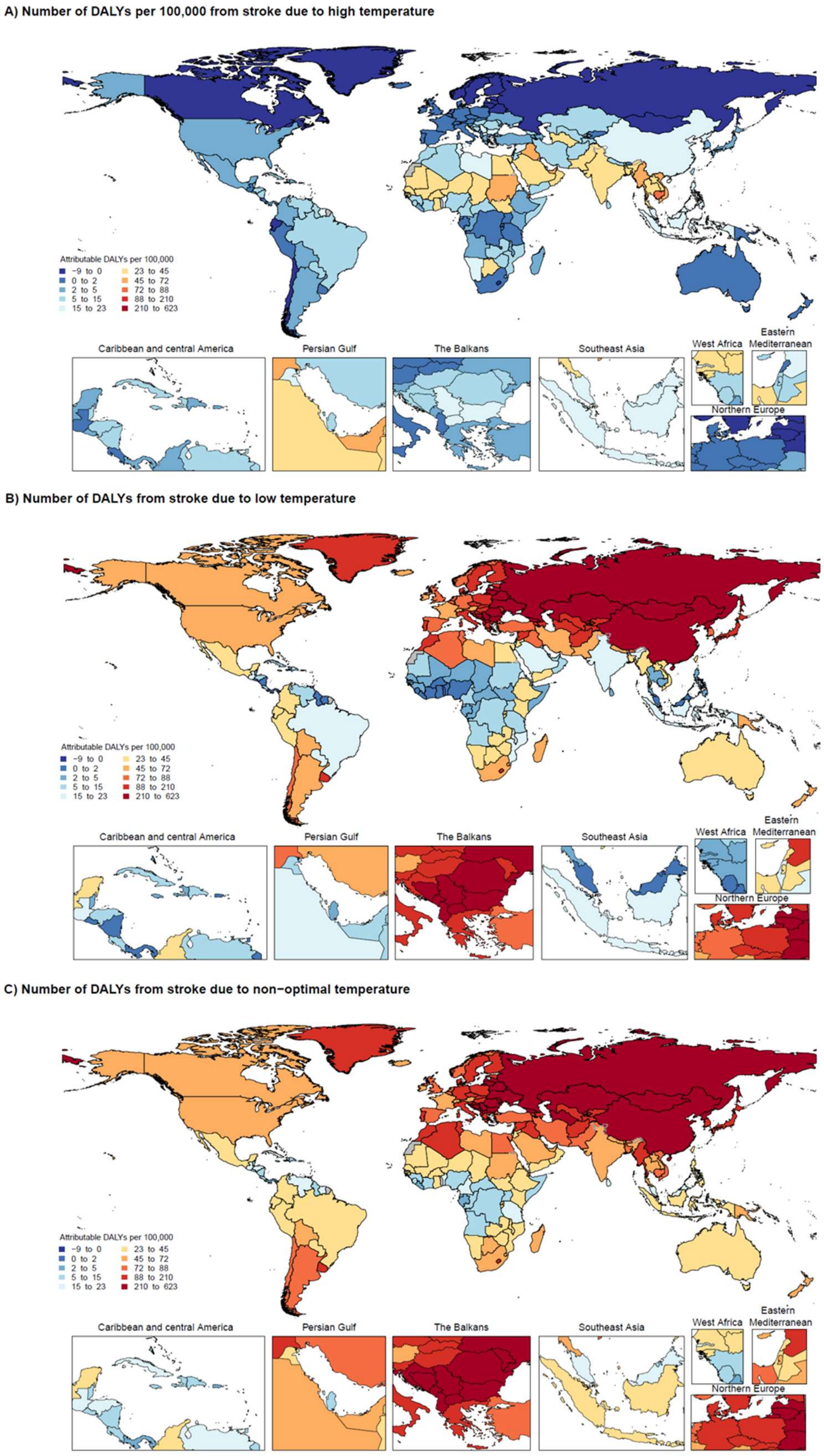

Figure S45: Spatial distribution of DALYs (per 100,000) due to hypertensive heart disease attributable to high temperature (A), low temperature (B), and non-optimal temperature (C) exposure, 2019

A) Number of DALYs per 100,000 from hypertensive heart disease due to high temperature

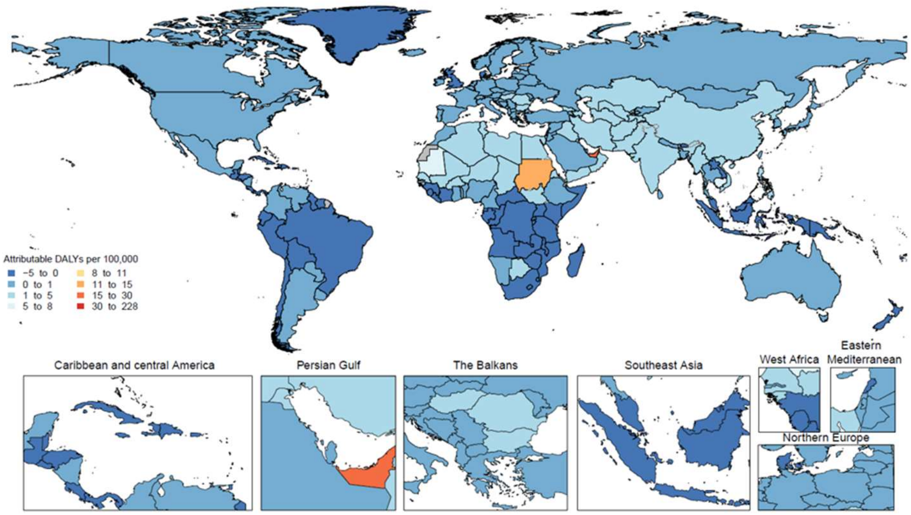

B) Number of DALYs from hypertensive heart disease due to low temperature

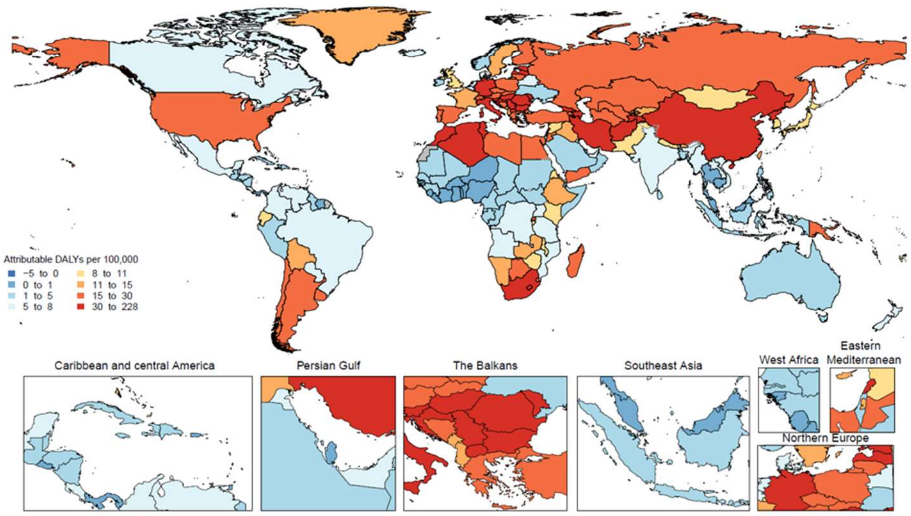

C) Number of DALYs from hypertensive heart disease due to non−optimal temperature

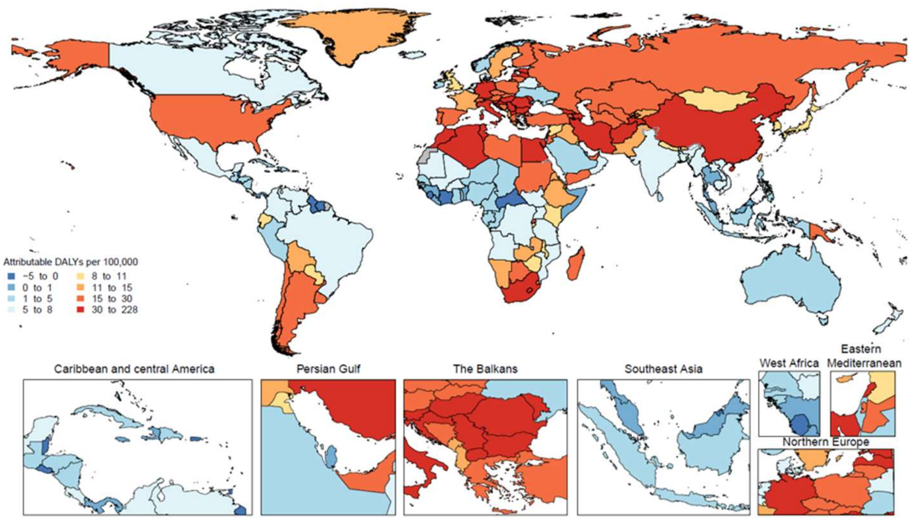

Figure S46: Spatial distribution of DALYs (per 100,000) due to cardiomyopathy and myocarditis attributable to high temperature (A), low temperature (B), and non-optimal temperature (C) exposure, 2019

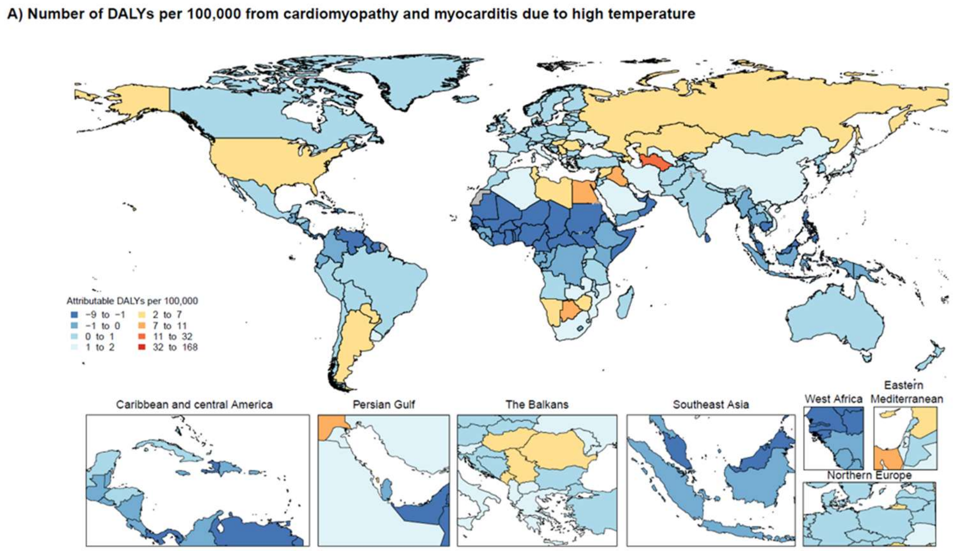

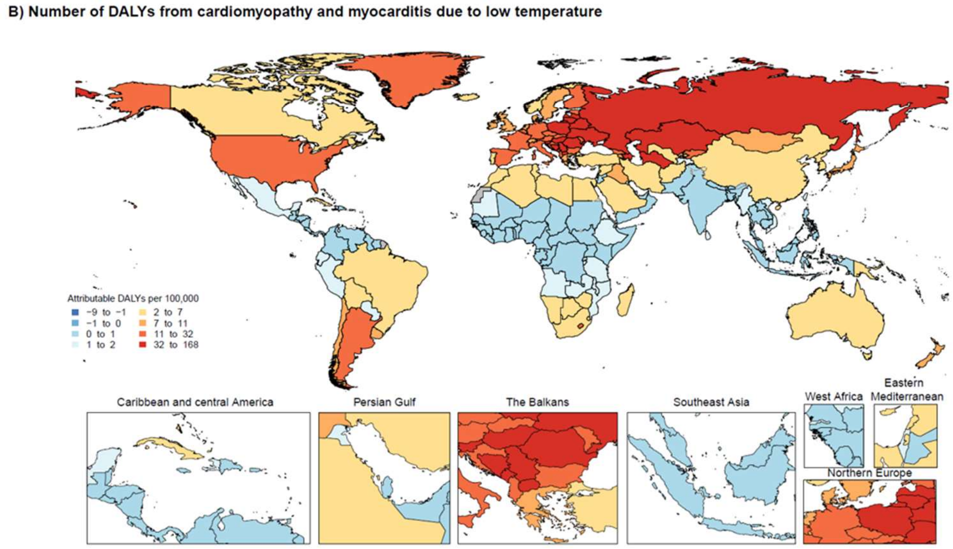

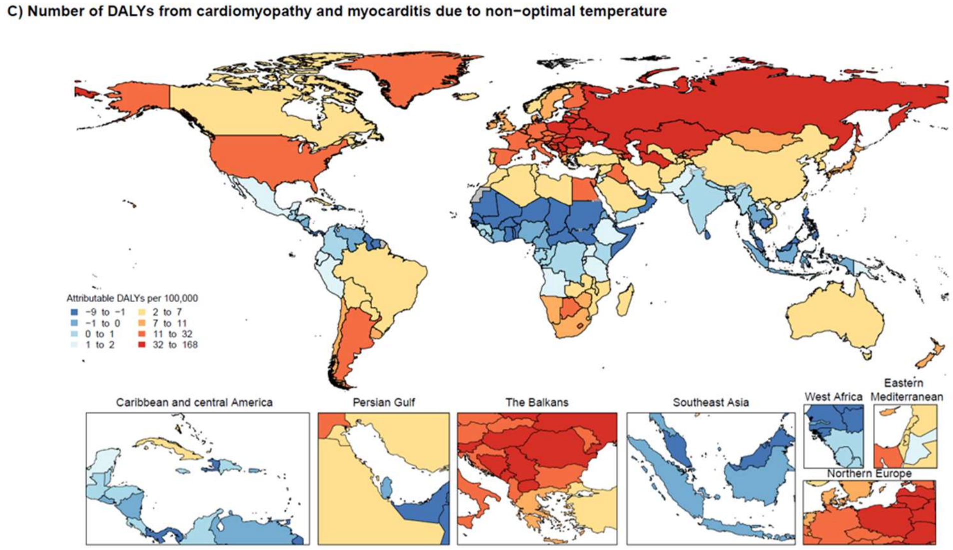

Figure S47: Spatial distribution of DALYs (per 100,000) due to chronic obstructive pulmonary disease attributable to high temperature (A), low temperature (B), and non-optimal temperature (C) exposure, 2019

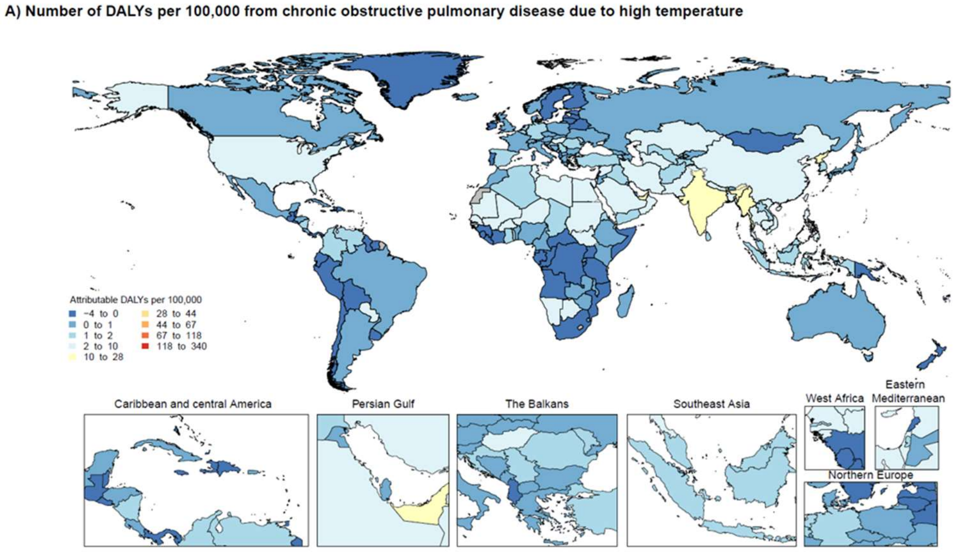

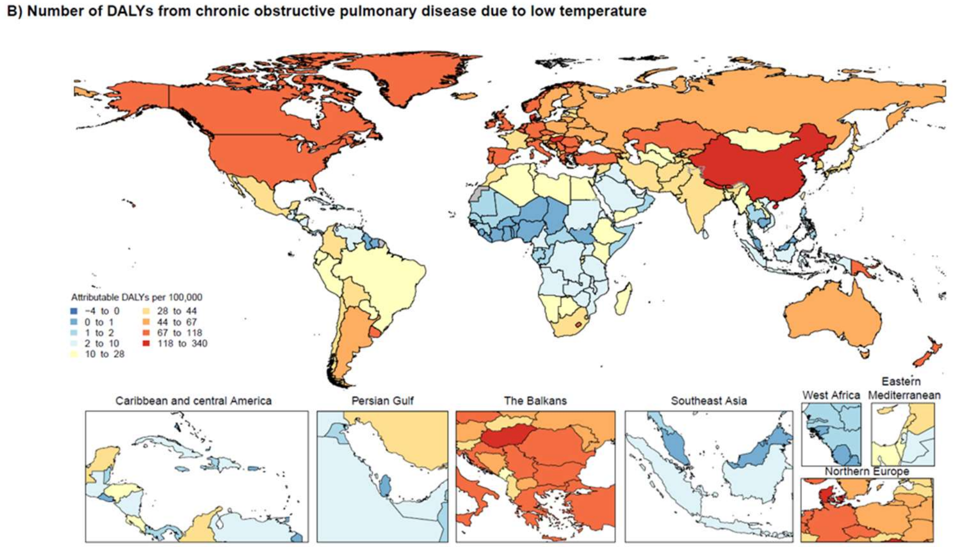

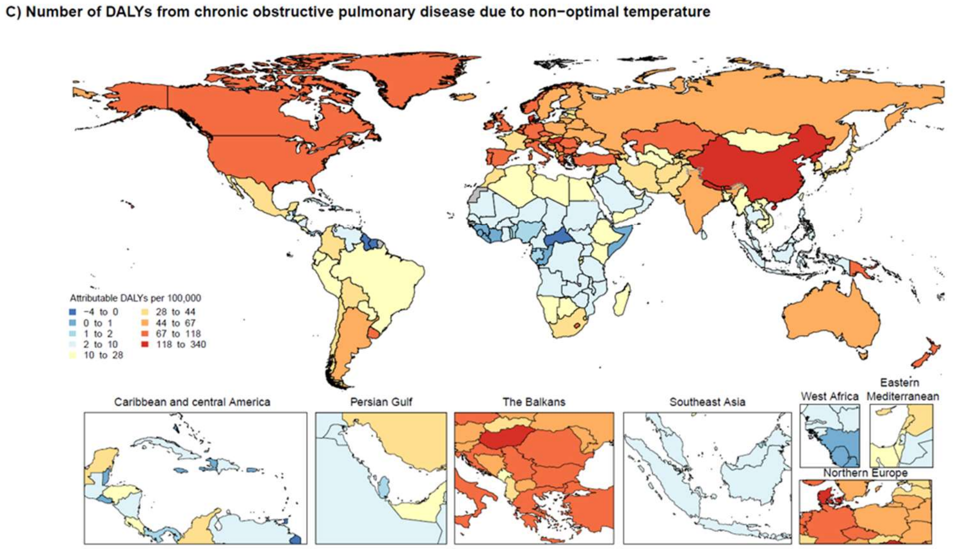

Figure S48: Spatial distribution of DALYs (per 100,000) due to diabetes mellitus attributable to high temperature (A), low temperature (B), and non-optimal temperature (C) exposure, 2019

A) Number of DALYs per 100,000 from diabetes mellitus due to high temperature

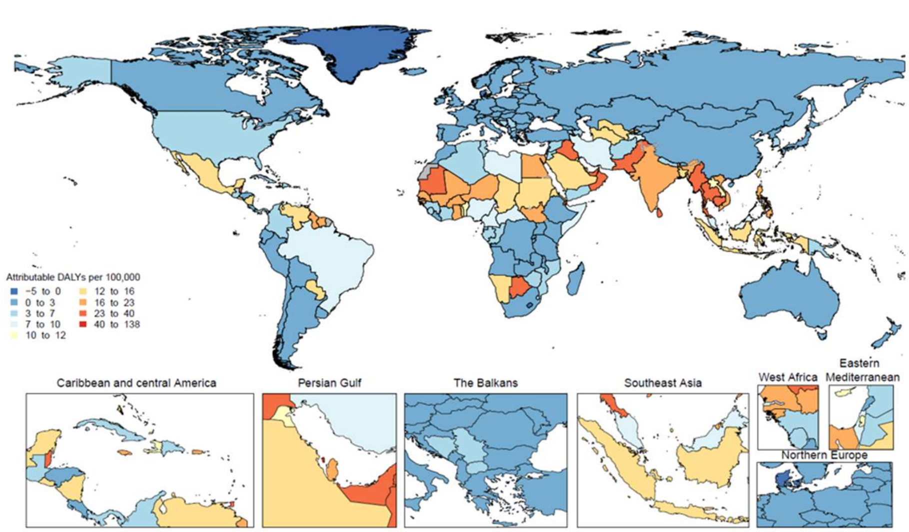

B) Number of DALYs from diabetes mellitus due to low temperature

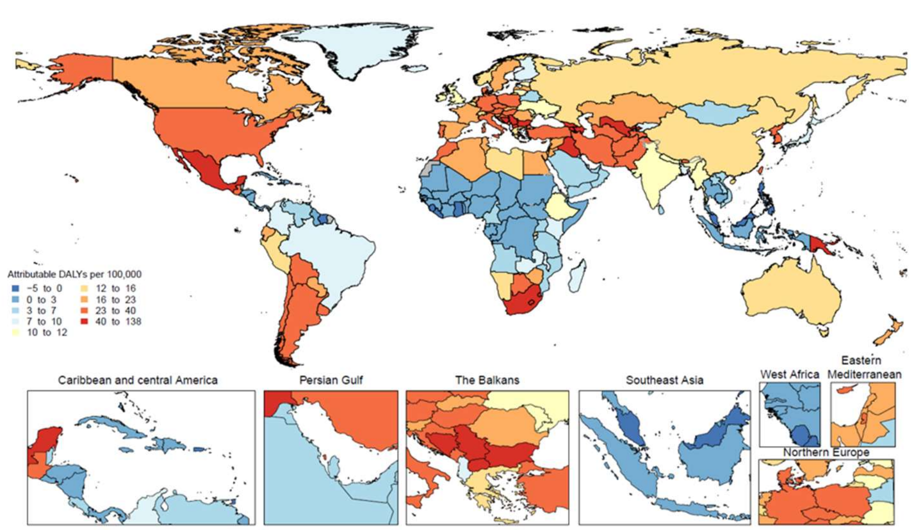

C) Number of DALYs from diabetes mellitus due to non−optimal temperature

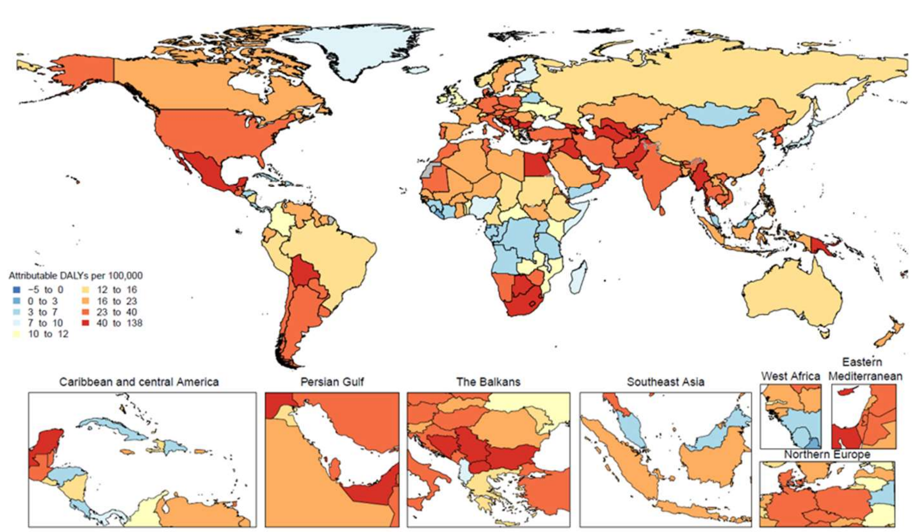

Figure S49: Spatial distribution of DALYs (per 100,000) due to chronic kidney disease attributable to high temperature (A), low temperature (B), and non-optimal temperature (C) exposure, 2019

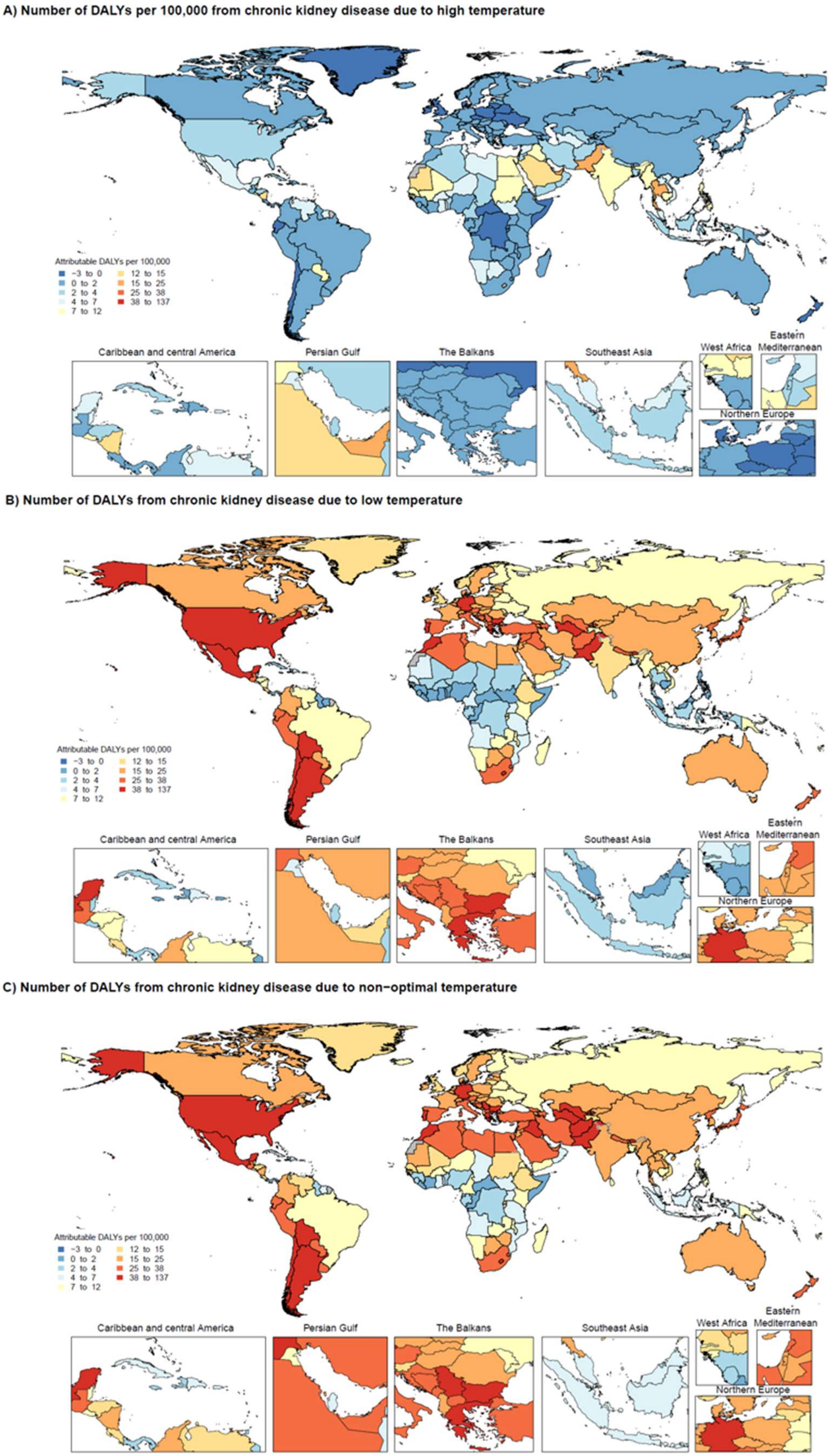

Figure S50: Spatial distribution of DALYs (per 100,000) due to road injuries attributable to high temperature (A), low temperature (B), and non-optimal temperature (C) exposure, 2019

A) Number of DALYs per 100,000 from road injuries due to high temperature

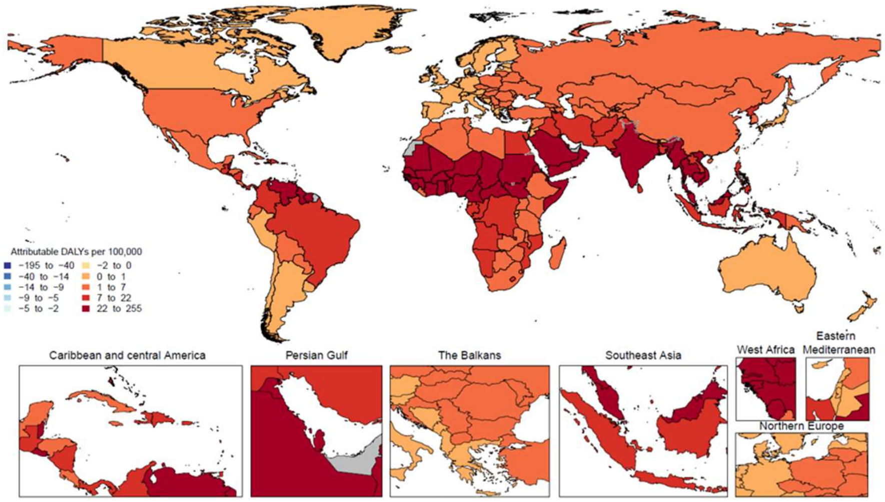

B) Number of DALYs from road injuries due to low temperature

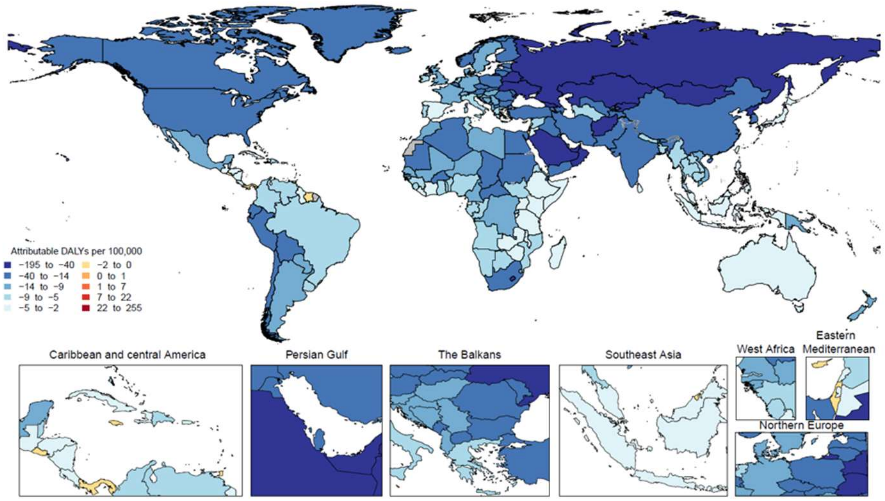

C) Number of DALYs from road injuries due to non−optimal temperature

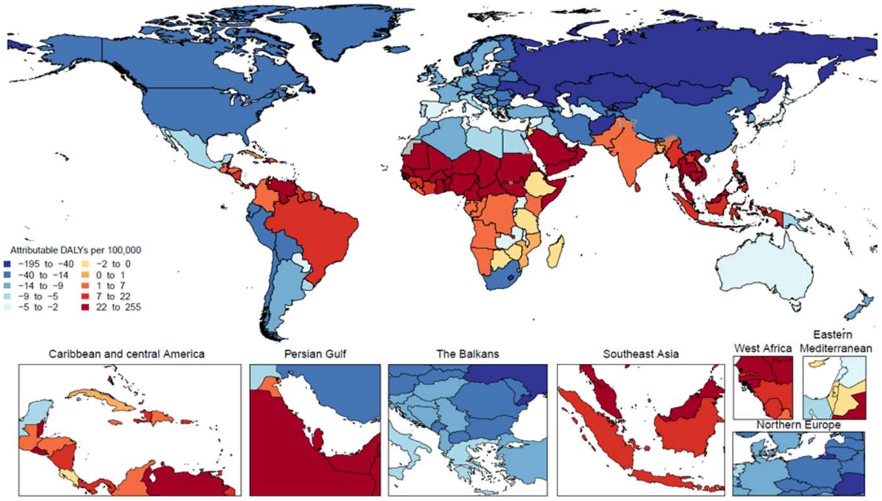

Figure S51: Spatial distribution of DALYs (per 100,000) due to other transport-related injuries attributable to high temperature (A), low temperature (B), and non-optimal temperature (C) exposure, 2019

A) Number of DALYs per 100,000 from other transport injuries due to high temperature

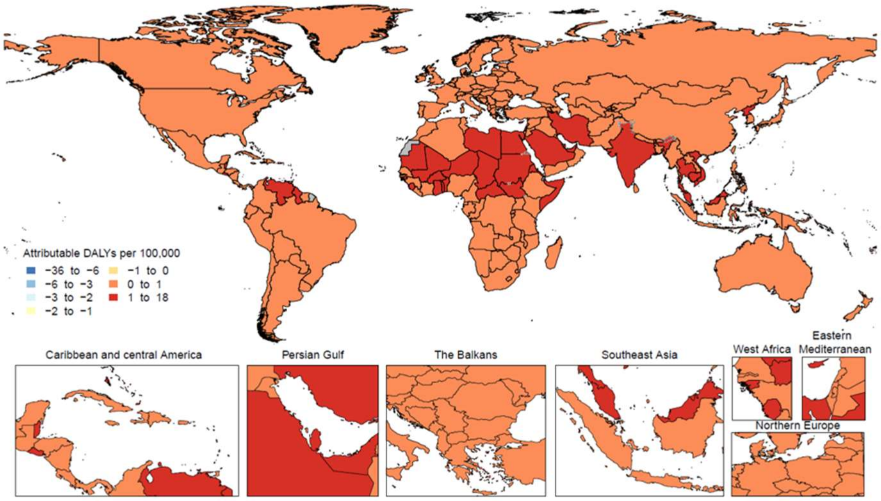

B) Number of DALYs from other transport injuries due to low temperature

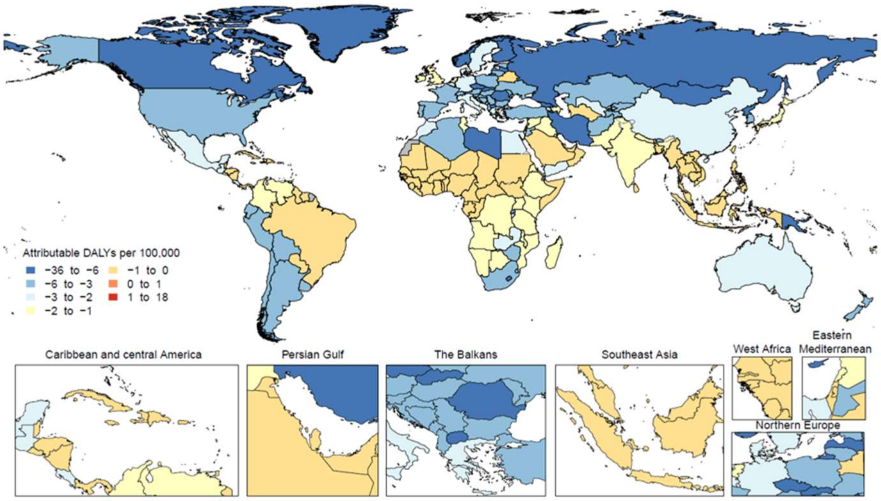

C) Number of DALYs from other transport injuries due to non−optimal temperature

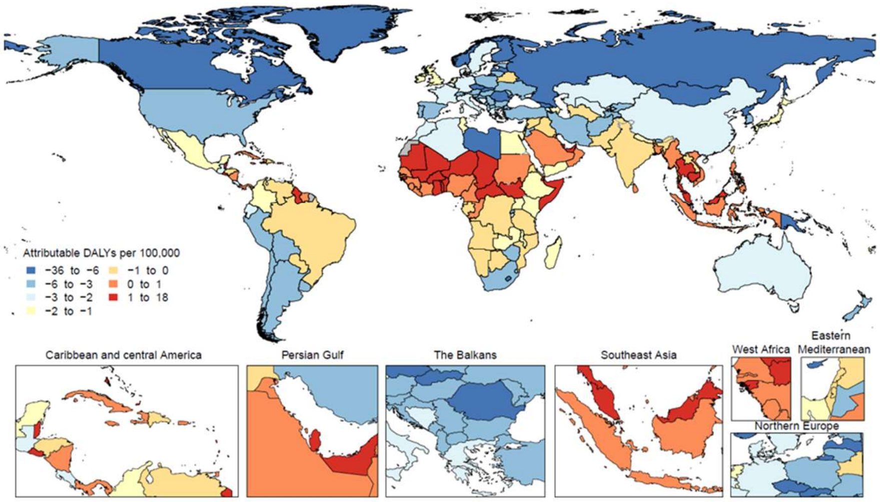

Figure S52: Spatial distribution of DALYs (per 100,000) due to drowning attributable to high temperature (A), low temperature (B), and non-optimal temperature (C) exposure, 2019

A) Number of DALYs per 100,000 from drowning due to high temperature

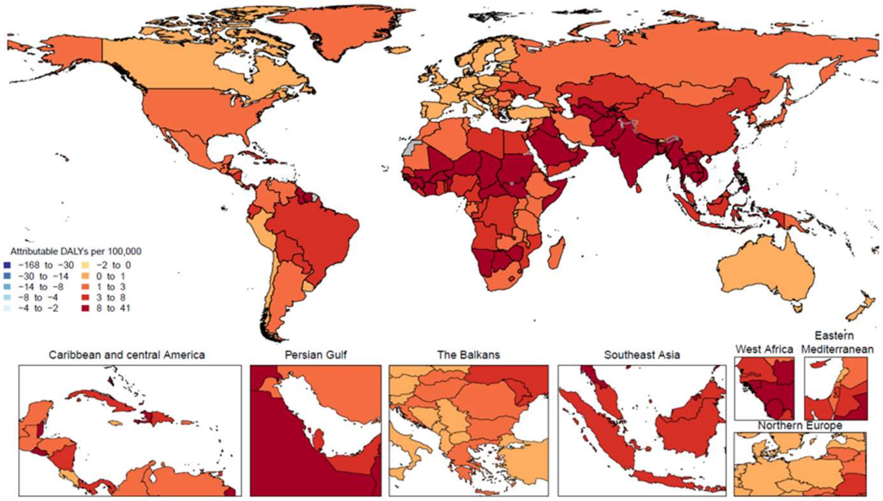

B) Number of DALYs from drowning due to low temperature

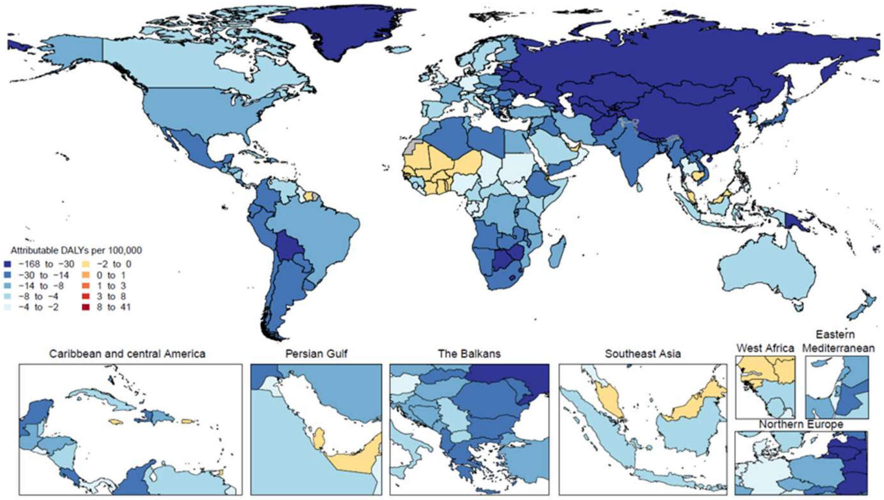

C) Number of DALYs from drowning due to non−optimal temperature

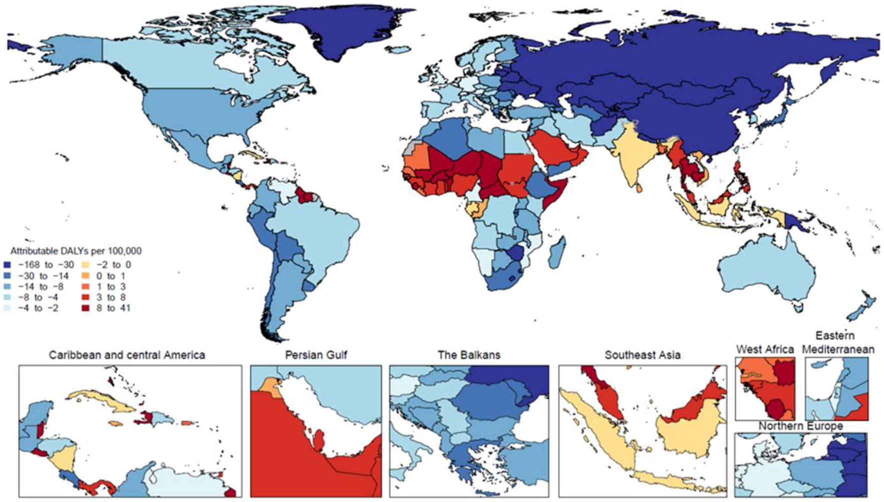

Figure S53: Spatial distribution of DALYs (per 100,000) due to mechanical injuries (exposure to mechanical forces) attributable to high temperature (A), low temperature (B), and non-optimal temperature (C) exposure, 2019

A) Number of DALYs per 100,000 from exposure to mechanical forces due to high temperature

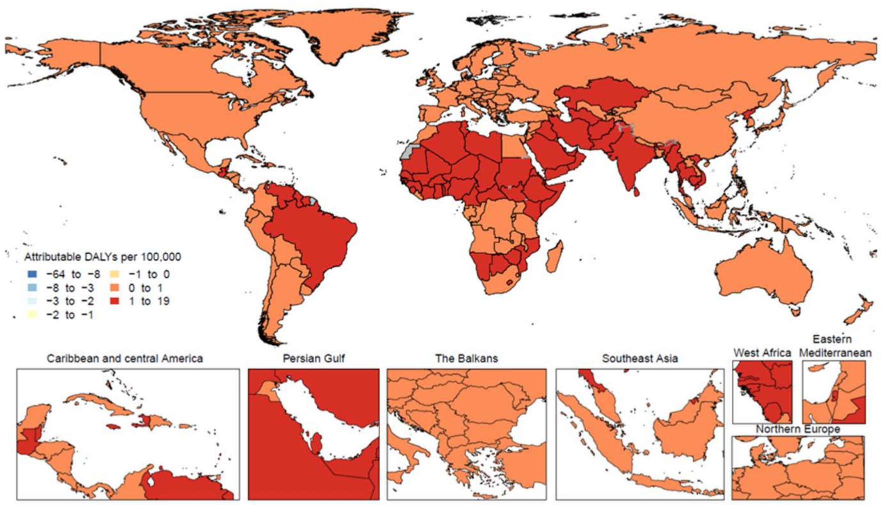

B) Number of DALYs from exposure to mechanical forces due to low temperature

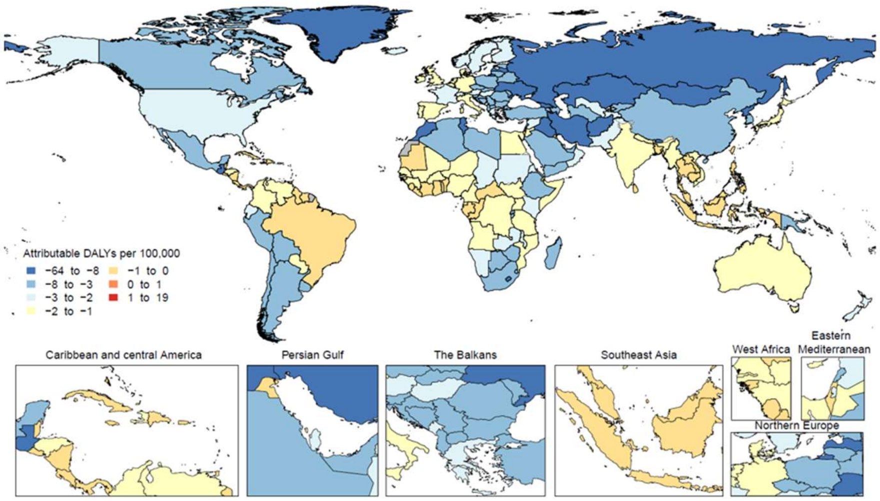

C) Number of DALYs from exposure to mechanical forces due to non−optimal temperature

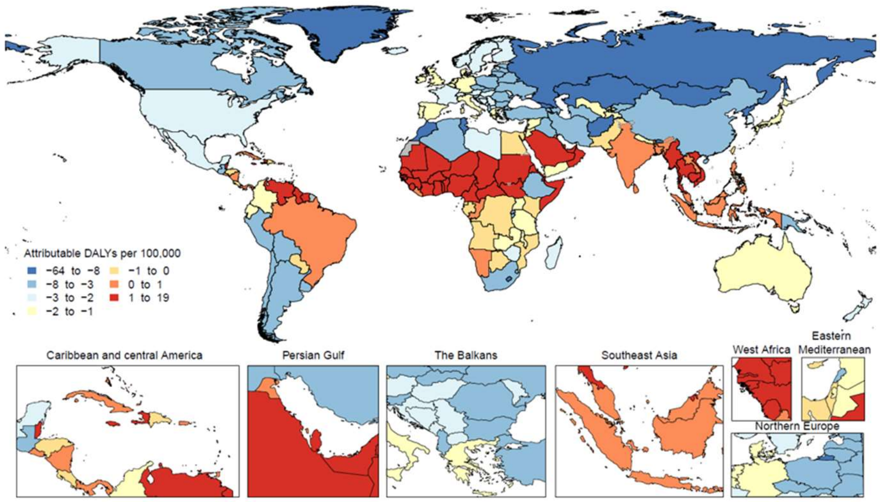

Figure S54: Spatial distribution of DALYs (per 100,000) due to animal-related injuries (animal contact) attributable to high temperature (A), low temperature (B), and non-optimal temperature (C) exposure, 2019

A) Number of DALYs per 100,000 from animal contact due to high temperature

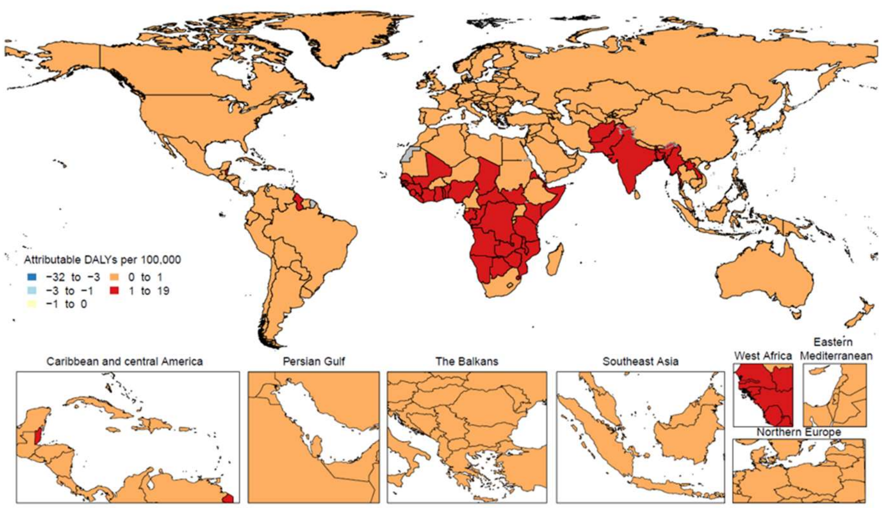

B) Number of DALYs from animal contact due to low temperature

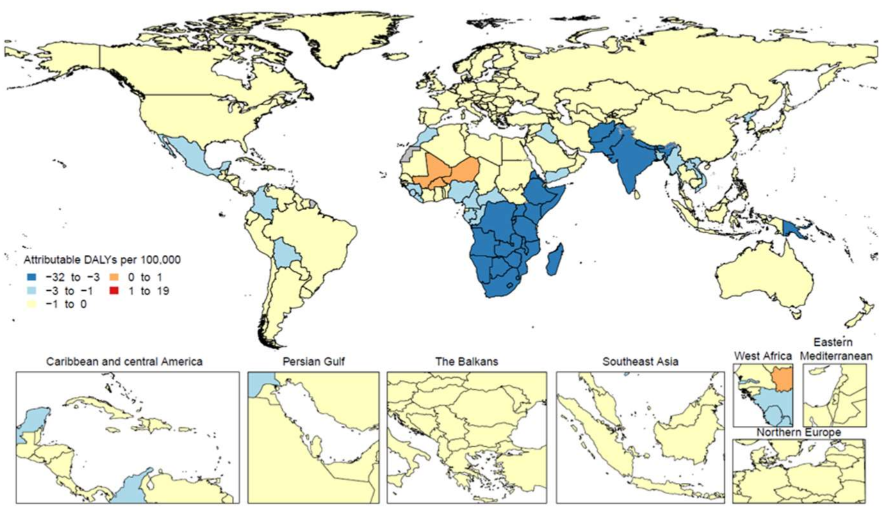

C) Number of DALYs from animal contact due to non−optimal temperature

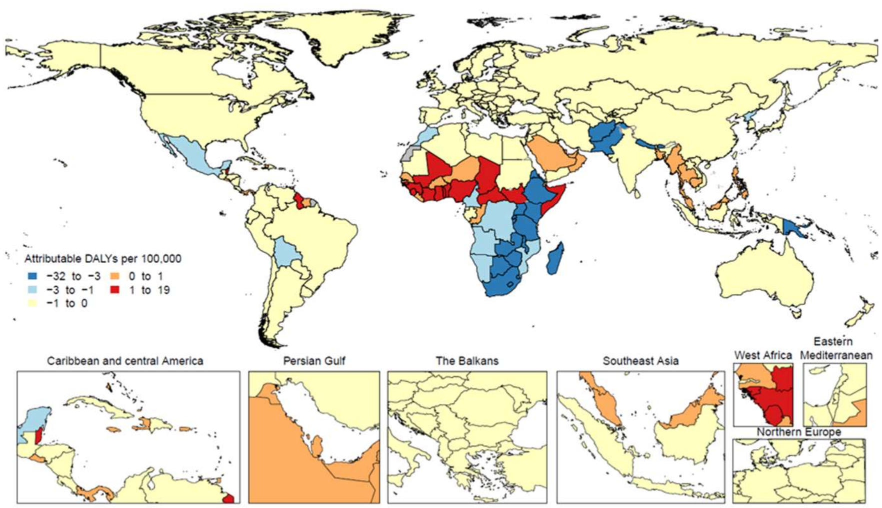

Figure S55: Spatial distribution of DALYs (per 100,000) due to disaster-related injuries (exposure to forces of nature) attributable to high temperature (A), low temperature (B), and non-optimal temperature (C) exposure, 2019

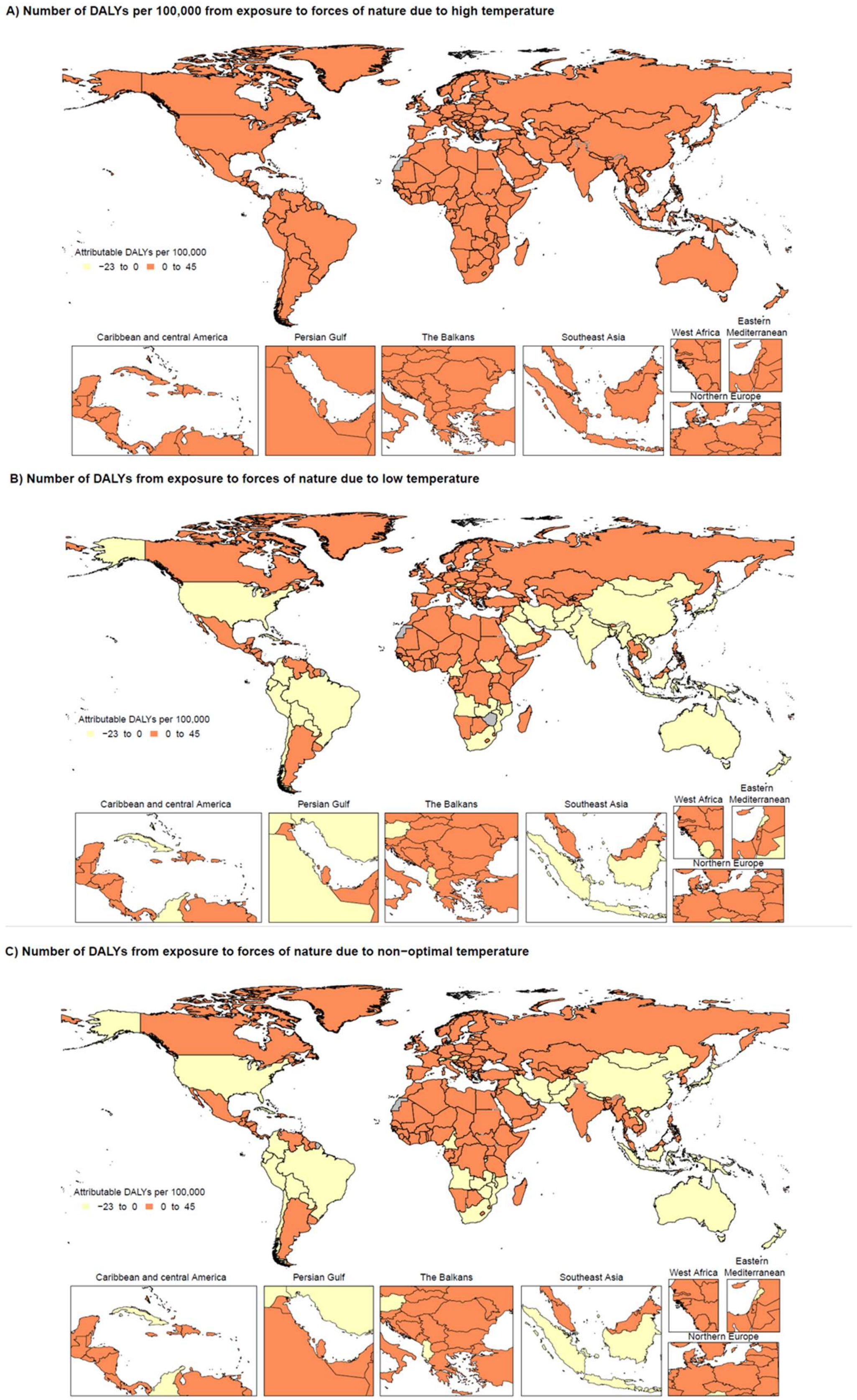

Figure S56: Spatial distribution of DALYs (per 100,000) due to other unintentional injuries attributable to high temperature (A), low temperature (B), and non-optimal temperature (C) exposure, 2019

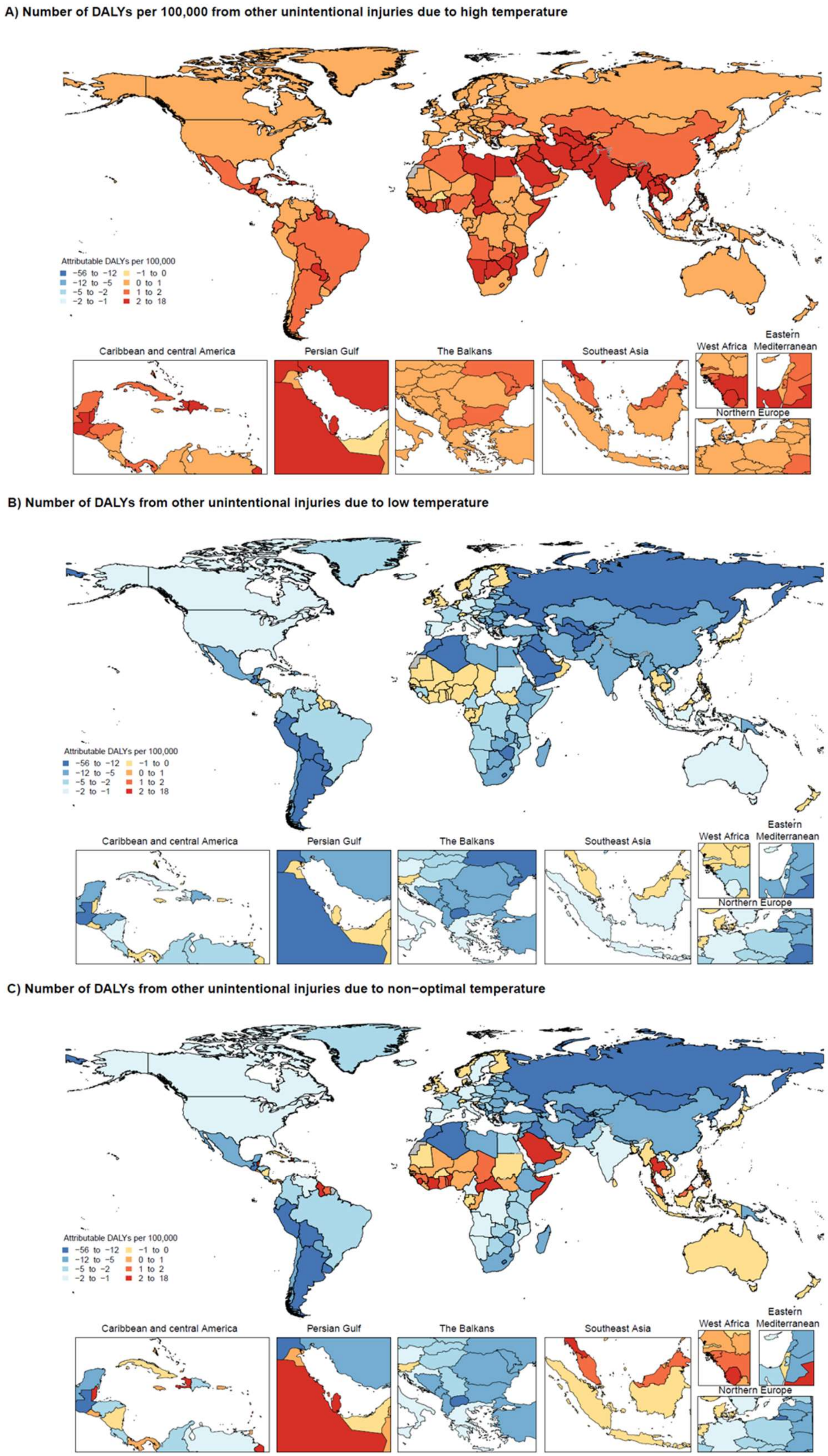

Figure S57: Spatial distribution of DALYs (per 100,000) due to suicide (self-harm) attributable to high temperature (A), low temperature (B), and non-optimal temperature (C) exposure, 2019

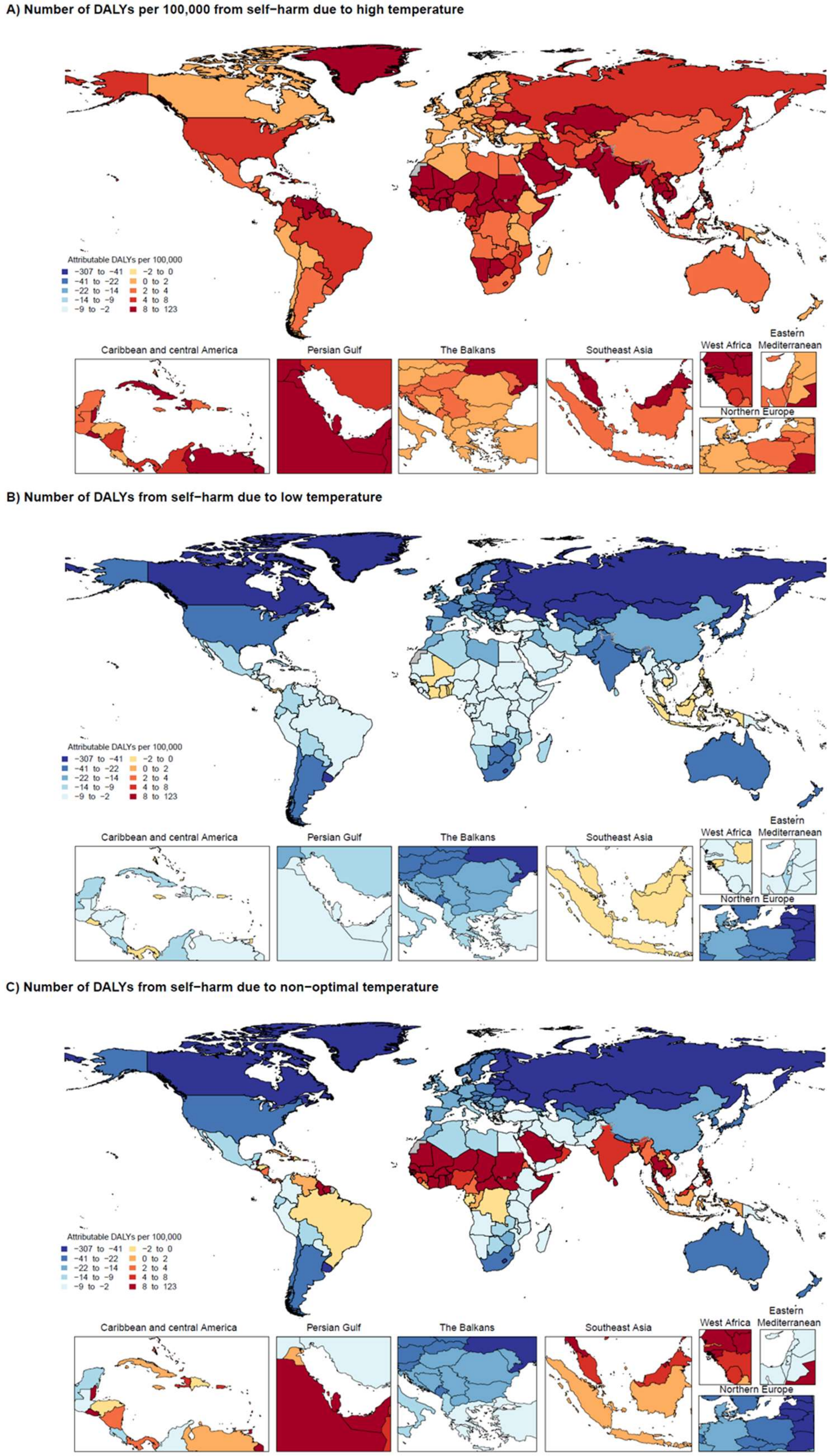

Figure S58: Spatial distribution of DALYs (per 100,000) due to homicide (interpersonal violence) attributable to high temperature (A), low temperature (B), and non-optimal temperature (C) exposure, 2019

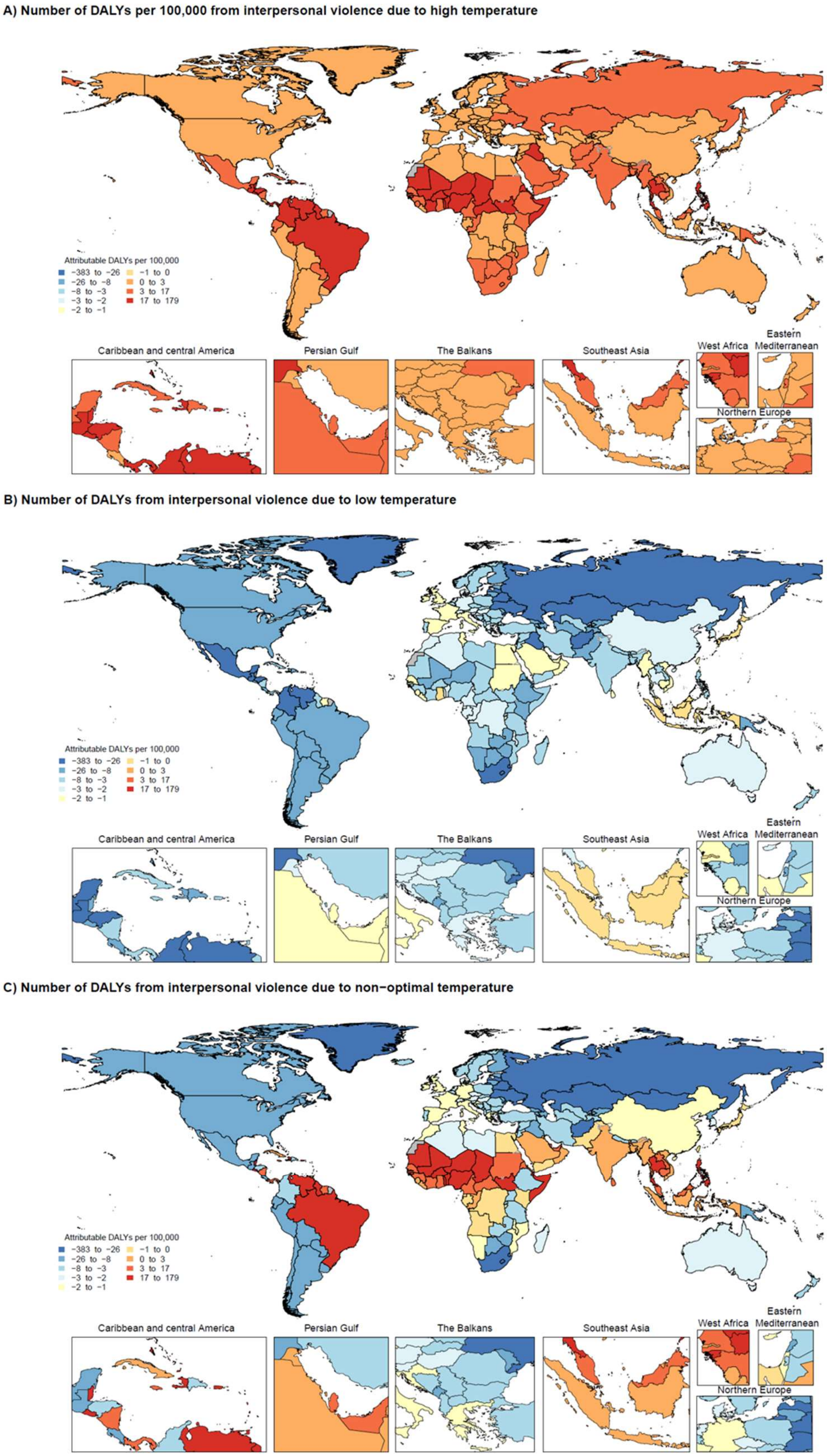

Section 4 Drivers of temperature-induced morbidity and mortality

Temperature affects human health in various ways, and a multitude of biological mechanisms have been described in the literature: vasoconstriction during cold episodes and vasodilation during hot episodes facilitate heat retention and release, respectively, and can strain the cardiovascular system.[16] Core temperature deviations trigger changes in haemoconcentration, increased plasma and blood viscosity, increased levels of certain proteins, and inflammatory changes.[17–20] Similarly, cold exposure triggers changes in humoral activity (eg, non-specific immune response and reduced phagocyte activity).[21,22] Moreover, cold can provoke bronchoconstriction, increasing the risk of pulmonary infections.[23] Beyond these biochemical mechanisms, social, behavioural, and environmental factors affect actual personal exposure to ambient temperatures and may shape adaptation and resilience.[24] Increased activity levels might increase risk for drowning or other accidents, and some evidence suggests that temperature-induced changes in serotonin function might increase impulsiveness and aggression and possibly lead to suicidal or criminal behaviours.[25,26] Further evidence indicates that heat decreases performance on physical and intellectual tasks.[27,28] We detail cause-specific biological mechanisms below:

Section 4.1 Cardiovascular disease

Section 4.1.1 Ischaemic heart disease

Ischaemic heart disease (IHD), which is the most common type of cardiovascular diseases, is triggered by a limited blood flow to the heart causing ischaemia, (cell starvation secondary to a lack of oxygen) of the heart's muscle cells.[29] Both high and low temperatures have been associated with increases in IHD.[21,30–33] Several bio-medical mechanisms, encompassing changes in blood pressure and blood chemistry, seem to be relevant:

Exposure to cold or a drop in temperature is associated with vasoconstriction and increased blood pressure[21,30], as well as higher cholesterol levels and heart rate[18,31,34]. Moreover, lower concentrations of high density lipoprotein (HDL) cholesterol have been found during periods of cold; low levels of HDL have been associated with poor cardiovascular health. Effects on the haemostatic system seem to be of especially high relevance. During cold periods higher levels of fibrinogen, the major plasma protein coagulation factor, have been found.[34] In addition, a protein that inhibits fibrinolysis, a process that inhibits blood clots from growing, is also raised.[18,21,34] Platelet count is augmented, increasing the risk of thrombocytosis. Additionally, exposure to cold has been associated with increased inflammation, as indicated by several markers such as C-reactive proteins and interleukin-6.[35] Several of the mechanisms outlined for cold temperatures also seem to be relevant for high temperatures, leading to increased blood viscosity and cholesterol levels.[31] Furthermore, exposure to hot temperatures may cause dehydration, salt depletion, and increased surface blood circulation, which can induce a failure of thermoregulation.[31]

Section 4.1.2 Stroke

Several previous studies demonstrated U- or V-shaped relationship between temperature and stroke mortality indicating both heat as well as cold effects.[36–38] However, findings are inconsistent and the impact of climate and weather on stroke has been intensely discussed within the scientific community.[39] In a recent literature review, Lianand colleagues [39] highlighted the importance of distinguishing between haemorrhage stroke (HS) and ischaemic stroke (IS). While hot temperature acted as a protective factor of HS, it acted as a risk factor for IS. IS is caused by a blocking of the brain artery, whereas HS occurs due to a brain aneurysm burst or a weakened blood vessel leak. The mechanisms leading to IS are likely to be similar to those described for IHD: acomplex interplay between blood chemistry, coagulation and inflammatory responses might ultimately lead to microvascular thrombosis.[40] These mechanisms are likely to be induced by high as well as low temperatures, leading to increased incidence on both ends of the temperature distribution.

The protective effect of high temperatures on HS is likely due to the peripheral vessels relaxation, lowering the afterload and reducing blood pressure, thus preventing the ongoing of HS. However, as heat continues, surface blood circulates faster, and more water comes out to accelerate the process of heat elimination with the implications of hemoconcentration and electrolyte imbalance.[41,42] Roy and Ray (2004)[43] found that hyperthermia was strongly associated with high mortality rates in both IS as well as HS, underlining the importance of high temperature for HS. As HS is less common than IS, the observed heat effect in our data is likely due to IS.

Section 4.2 Respiratory diseases

Section 4.2.1 Upper and lower respiratory infection

Exposure to low temperatures can lead to changes in humoral activity (non-specific immune response) characterised by reduced phagocyte activity, thus hampering the host's ability to destroy viruses and bacteria.[21,44] Moreover, cold might provoke a physiological reaction of the respiratory tract, for example through bronchoconstriction, leading to an increased risk of pulmonary infections. The presence of inflammatory cells in sputum after breathing cold air indicates that cold air induces inflammatory changes in the airways.[16,45,46] In addition, weather and temperature play a crucial role for pathogens, affecting the survival and replication of disease-provoking agents. Different agents favor different meteorological and hydrological conditions. High temperatures generally promote the growth of bacteria while high humidity can favor the survival of pathogens in droplets.[47] The relationship between heat and respiratory disease is less understood: respiratory tract irritations due to increased breathing rates as well as socio/cultural aspect as the usage of air conditioning or air pollution-temperature interactions might be crucial.

Section 4.2.2 Chronic obstructive pulmonary disease (COPD)

Extremes of temperature—both heat and cold—have been associated with increased respiratory morbidity in COPD. Several studies highlighted an increased risk of dying from low temperatures among those with COPD.[48,49] Cold impacts lung functions and increases the risk of exacerbation.[50] Additionally, bronchoconstriction and inflammatory responses to low temperatures might aggravate COPD symptoms.[16,23,45,46,51] A recent study suggests the role of cold-induced mucus hypersecretion, a major clinical and pathological feature in COPD (and asthma), which is associated with the activation of the host inflammatory response.[52] Similarly, a multitude of studies have shown that individuals with pre-existing respiratory diseases, such as COPD, have an increased vulnerability to heat exposure.[53–57] In terms of high temperature effects and interaction between heat and air pollution has been suggested in the literature.[48,58–63] Nevertheless, findings are inconstant with several studies not validating interactive effects[54,64,65] or finding interaction between temperature and air pollution for all-cause or cardiovascular but not respiratory diseases.[66,67] In addition to interaction effects, Hansel et al.[68] suggest the relevance to bronchoconstriction from breathing hot and humid air, similar to those observed for asthma patients.[69]

Section 4.3 Chronic kidney disease (CKD)

Several studies have highlighted the impact of high temperature on renal disease.[70–72] Heat most plausibly exerts an effect through dehydration, which has been linked to acute as well as CKD in various studies.[73,74] However, a precise mechanism has not been established so far.[75–78] A proposed mechanism is that prolonged elevated vasopressin secretion, induced by chronic dehydration, contributes to progressive tubulointerstitial damage, predisposing to CKD.[75,79] In addition, causes and co-morbidities of CKD, such as diabetes, obesity, and hypertension[80] might aggravate the impact of heat as these are similarly affected by temperature, as outlined elsewhere in this document. Most research on temperature and renal disease relates to adverse effects of high temperatures. To our knowledge, there is no body of evidence describing the impacts of cold on CKD, as observed in our data. Potential mechanisms might be cold-induced inflammatory responses, as described for cardiovascular disease.

Section 4.4 Diabetes

Several studies highlighted temperature effects in diabetes patients with increased susceptibility to both cold and hot temperatures.[28,31,81–92] Increased numbers of emergency department visits and hospitalisations, increased occurrence of dehydration and electrolyte abnormalities, and higher death rate have been observed for diabetics than for non-diabetics.[54,83–91,93] Patients with diabetes may have increased susceptibility to heat due to the impairment of thermoregulatory mechanisms with reported lower skin blood flow and sweating response.[81,94,95] Additionally, weakened orthostatic responses at elevated temperatures have been observed.[94,95] Moreover, prolonged exposure to high temperatures can alter insulin kinetics and stability.[94] This has strong implications for cardiovascular regulations and glycemic control.[96] In contrast, fewer studies have examined the effects of cold exposure on individuals with diabetes.[96] Research outlined an apparent loss of efferent vasomotor control during diabetic neuropathy during cold periods which might lead to increased susceptibility.[94,95]

Section 5 Data source citations

Table S7. Vital registration data source citations by location and year

Country	Year	Citation
Brazil	2016	Ministry of Health (Brazil). Brazil Mortality Information System - Deaths 2016. Rio de Janeiro, Brazil: Ministry of Health (Brazil).
Brazil	2015	Ministry of Health (Brazil). Brazil Mortality Information System - Deaths 2015. Rio de Janeiro, Brazil: Ministry of Health (Brazil).
Brazil	2014	Ministry of Health (Brazil). Brazil Mortality Information System - Deaths 2014. Rio de Janeiro, Brazil: Ministry of Health (Brazil).
Brazil	2013	Ministry of Health (Brazil). Brazil Mortality Information System - Deaths 2013. Rio de Janeiro, Brazil: Ministry of Health (Brazil).
Brazil	2012	Ministry of Health (Brazil). Brazil Mortality Information System - Deaths 2012. Rio de Janeiro, Brazil: Ministry of Health (Brazil).
Brazil	2011	Ministry of Health (Brazil). Brazil Mortality Information System - Deaths 2011. Rio de Janeiro, Brazil: Ministry of Health (Brazil).
Brazil	2010	Ministry of Health (Brazil). Brazil Mortality Information System - Deaths 2010. Rio de Janeiro, Brazil: Ministry of Health (Brazil).
Brazil	2009	Ministry of Health (Brazil). Brazil Mortality Information System - Deaths 2009. Rio de Janeiro, Brazil: Ministry of Health (Brazil).
Brazil	2008	Ministry of Health (Brazil). Brazil Mortality Information System - Deaths 2008. Rio de Janeiro, Brazil: Ministry of Health (Brazil).
Brazil	2007	Ministry of Health (Brazil). Brazil Mortality Information System - Deaths 2007. Rio de Janeiro, Brazil: Ministry of Health (Brazil).
Brazil	2006	Ministry of Health (Brazil). Brazil Mortality Information System - Deaths 2006. Rio de Janeiro, Brazil: Ministry of Health (Brazil).
Brazil	2005	Ministry of Health (Brazil). Brazil Mortality Information System - Deaths 2005. Rio de Janeiro, Brazil: Ministry of Health (Brazil).
Brazil	2004	Ministry of Health (Brazil). Brazil Mortality Information System - Deaths 2004. Rio de Janeiro, Brazil: Ministry of Health (Brazil).
Brazil	2003	Ministry of Health (Brazil). Brazil Mortality Information System - Deaths 2003. Rio de Janeiro, Brazil: Ministry of Health (Brazil).
Brazil	2002	Ministry of Health (Brazil). Brazil Mortality Information System - Deaths 2002. Rio de Janeiro, Brazil: Ministry of Health (Brazil).
Brazil	2001	Ministry of Health (Brazil). Brazil Mortality Information System - Deaths 2001. Rio de Janeiro, Brazil: Ministry of Health (Brazil).
Brazil	2000	Ministry of Health (Brazil). Brazil Mortality Information System - Deaths 2000. Rio de Janeiro, Brazil: Ministry of Health (Brazil).
Brazil	1999	Ministry of Health (Brazil). Brazil Mortality Information System - Deaths 1999. Rio de Janeiro, Brazil: Ministry of Health (Brazil).
Chile	2011	Department of Statistics and Health Information, Ministry of Health (Chile), National Institute of Statistics (Chile). Chile Vital Statistics - Deaths 2011. Santiago, Chile: Department of Statistics and Health Information, Ministry of Health (Chile).
Chile	2010	Department of Statistics and Health Information, Ministry of Health (Chile), National Institute of Statistics (Chile). Chile Vital Statistics - Deaths 2010. Santiago, Chile: Department of Statistics and Health Information, Ministry of Health (Chile).
Chile	2009	Department of Statistics and Health Information, Ministry of Health (Chile), National Institute of Statistics (Chile). Chile Vital Statistics - Deaths 2009. Santiago, Chile: Department of Statistics and Health Information, Ministry of Health (Chile).
Chile	1996	Department of Statistics and Health Information, Ministry of Health (Chile), National Institute of Statistics (Chile). Chile Vital Statistics - Deaths 1996. Santiago, Chile: Department of Statistics and Health Information, Ministry of Health (Chile).

Chile	1995	Department of Statistics and Health Information, Ministry of Health (Chile), National Institute of Statistics (Chile). Chile Vital Statistics - Deaths 1995. Santiago, Chile: Department of Statistics and Health Information, Ministry of Health (Chile).
Chile	1994	Department of Statistics and Health Information, Ministry of Health (Chile), National Institute of Statistics (Chile). Chile Vital Statistics - Deaths 1994. Santiago, Chile: Department of Statistics and Health Information, Ministry of Health (Chile).
Chile	1993	Department of Statistics and Health Information, Ministry of Health (Chile), National Institute of Statistics (Chile). Chile Vital Statistics - Deaths 1993. Santiago, Chile: Department of Statistics and Health Information, Ministry of Health (Chile).
Chile	1992	Department of Statistics and Health Information, Ministry of Health (Chile), National Institute of Statistics (Chile). Chile Vital Statistics - Deaths 1992. Santiago, Chile: Department of Statistics and Health Information, Ministry of Health (Chile).
Chile	1991	Department of Statistics and Health Information, Ministry of Health (Chile), National Institute of Statistics (Chile). Chile Vital Statistics - Deaths 1991. Santiago, Chile: Department of Statistics and Health Information, Ministry of Health (Chile).
Chile	1990	Department of Statistics and Health Information, Ministry of Health (Chile), National Institute of Statistics (Chile). Chile Vital Statistics - Deaths 1990. Santiago, Chile: Department of Statistics and Health Information, Ministry of Health (Chile).
China	2016	Chinese Center for Disease Control and Prevention (CCDC), Ministry of Health (China). China Vital Registration - Death Counts by Cause, County, Age, and Daily Temperature 2016.
China	2015	Chinese Center for Disease Control and Prevention (CCDC), Ministry of Health (China). China Vital Registration - Death Counts by Cause, County, Age, and Daily Temperature 2015.
Colombia	2005	National Administrative Department of Statistics (DANE) (Colombia). Colombia Vital Statistics - Deaths 2005. Bogotá, Colombia: National Administrative Department of Statistics (DANE) (Colombia).
Colombia	2004	National Administrative Department of Statistics (DANE) (Colombia). Colombia Vital Statistics - Deaths 2004. Bogotá, Colombia: National Administrative Department of Statistics (DANE) (Colombia).
Colombia	2003	National Administrative Department of Statistics (DANE) (Colombia). Colombia Vital Statistics - Deaths 2003. Bogotá, Colombia: National Administrative Department of Statistics (DANE) (Colombia).
Colombia	2002	National Administrative Department of Statistics (DANE) (Colombia). Colombia Vital Statistics - Deaths 2002. Bogotá, Colombia: National Administrative Department of Statistics (DANE) (Colombia).
Colombia	2001	National Administrative Department of Statistics (DANE) (Colombia). Colombia Vital Statistics - Deaths 2001. Bogotá, Colombia: National Administrative Department of Statistics (DANE) (Colombia).
Guatemala	2016	National Statistics Institute (Guatemala). Guatemala Vital Statistics 2016. Guatemala City, Guatemala: National Statistics Institute (Guatemala), 2017.
Guatemala	2015	National Statistics Institute (Guatemala). Guatemala Vital Statistics 2015. Guatemala City, Guatemala: National Statistics Institute (Guatemala), 2016.
Guatemala	2014	National Statistics Institute (Guatemala). Guatemala Vital Statistics 2014. Guatemala City, Guatemala: National Statistics Institute (Guatemala), 2015.
Guatemala	2013	National Statistics Institute (Guatemala). Guatemala Vital Statistics 2013. Guatemala City, Guatemala: National Statistics Institute (Guatemala), 2014.
Guatemala	2012	National Statistics Institute (Guatemala). Guatemala Vital Statistics 2012. Guatemala City, Guatemala: National Statistics Institute (Guatemala), 2013.
Guatemala	2011	National Statistics Institute (Guatemala). Guatemala Vital Statistics 2011. Guatemala City, Guatemala: National Statistics Institute (Guatemala), 2012.
Guatemala	2010	National Statistics Institute (Guatemala). Guatemala Vital Statistics 2010. Guatemala City, Guatemala: National Statistics Institute (Guatemala), 2011.
Guatemala	2009	National Statistics Institute (Guatemala). Guatemala Vital Statistics 2009. Guatemala City, Guatemala: National Statistics Institute (Guatemala), 2010.
Mexico	2015	National Institute of Statistics and Geography (INEGI) (Mexico), Secretariat of Health (Mexico). Mexico Vital Registration - Multiple Causes of Death 2015.
Mexico	2014	National Institute of Statistics and Geography (INEGI) (Mexico), Secretariat of Health (Mexico). Mexico Vital Registration - Multiple Causes of Death 2014.
Mexico	2013	National Institute of Statistics and Geography (INEGI) (Mexico), Secretariat of Health (Mexico). Mexico Vital Registration - Multiple Causes of Death 2013.

Mexico	2012	National Institute of Statistics and Geography (INEGI) (Mexico), Secretariat of Health (Mexico). Mexico Vital Registration - Multiple Causes of Death 2012.
Mexico	2011	National Institute of Statistics and Geography (INEGI) (Mexico), Secretariat of Health (Mexico). Mexico Vital Registration - Multiple Causes of Death 2011.
Mexico	2010	National Institute of Statistics and Geography (INEGI) (Mexico), Secretariat of Health (Mexico). Mexico Vital Registration - Multiple Causes of Death 2010.
Mexico	2009	National Institute of Statistics and Geography (INEGI) (Mexico), Secretariat of Health (Mexico). Mexico Vital Registration - Multiple Causes of Death 2009.
Mexico	2008	National Institute of Statistics and Geography (INEGI) (Mexico), Secretariat of Health (Mexico). Mexico Vital Registration - Deaths 2008.
Mexico	2007	National Institute of Statistics and Geography (INEGI) (Mexico), Secretariat of Health (Mexico). Mexico Vital Registration - Deaths 2007.
Mexico	2006	National Institute of Statistics and Geography (INEGI) (Mexico), Secretariat of Health (Mexico). Mexico Vital Registration - Deaths 2006.
Mexico	2005	National Institute of Statistics and Geography (INEGI) (Mexico), Secretariat of Health (Mexico). Mexico Vital Registration - Deaths 2005.
Mexico	2004	National Institute of Statistics and Geography (INEGI) (Mexico), Secretariat of Health (Mexico). Mexico Vital Registration - Deaths 2004.
Mexico	2003	National Institute of Statistics and Geography (INEGI) (Mexico), Secretariat of Health (Mexico). Mexico Vital Registration - Deaths 2003.
Mexico	2002	National Institute of Statistics and Geography (INEGI) (Mexico), Secretariat of Health (Mexico). Mexico Vital Registration - Deaths 2002.
Mexico	2001	National Institute of Statistics and Geography (INEGI) (Mexico), Secretariat of Health (Mexico). Mexico Vital Registration - Deaths 2001.
Mexico	2000	National Institute of Statistics and Geography (INEGI) (Mexico), Secretariat of Health (Mexico). Mexico Vital Registration - Deaths 2000.
Mexico	1999	National Institute of Statistics and Geography (INEGI) (Mexico), Secretariat of Health (Mexico). Mexico Vital Registration - Deaths 1999.
Mexico	1998	National Institute of Statistics and Geography (INEGI) (Mexico), Secretariat of Health (Mexico). Mexico Vital Registration - Deaths 1998.
Mexico	1997	National Institute of Statistics and Geography (INEGI) (Mexico), Secretariat of Health (Mexico). Mexico Vital Registration - Deaths 1997.
Mexico	1996	National Institute of Statistics and Geography (INEGI) (Mexico), Secretariat of Health (Mexico). Mexico Vital Registration - Deaths 1996.
New Zealand	2014	Ministry of Health (New Zealand). New Zealand Mortality Collection 2014. Wellington, New Zealand: Ministry of Health (New Zealand).
New Zealand	2013	Ministry of Health (New Zealand). New Zealand Mortality Collection 2013. Wellington, New Zealand: Ministry of Health (New Zealand).
New Zealand	2012	Ministry of Health (New Zealand). New Zealand Mortality Collection 2012. Wellington, New Zealand: Ministry of Health (New Zealand).
New Zealand	2011	Ministry of Health (New Zealand). New Zealand Mortality Collection 2011. Wellington, New Zealand: Ministry of Health (New Zealand).
New Zealand	2010	Ministry of Health (New Zealand). New Zealand Mortality Collection 2010. Wellington, New Zealand: Ministry of Health (New Zealand).
New Zealand	2009	Ministry of Health (New Zealand). New Zealand Mortality Collection 2009. Wellington, New Zealand: Ministry of Health (New Zealand).
New Zealand	2008	Ministry of Health (New Zealand). New Zealand Mortality Collection 2008. Wellington, New Zealand: Ministry of Health (New Zealand).
New Zealand	2007	Ministry of Health (New Zealand). New Zealand Mortality Collection 2007. Wellington, New Zealand: Ministry of Health (New Zealand).

New Zealand	2006	Ministry of Health (New Zealand). New Zealand Mortality Collection 2006. Wellington, New Zealand: Ministry of Health (New Zealand).
New Zealand	2005	Ministry of Health (New Zealand). New Zealand Mortality Collection 2005. Wellington, New Zealand: Ministry of Health (New Zealand).
New Zealand	2004	Ministry of Health (New Zealand). New Zealand Mortality Collection 2004. Wellington, New Zealand: Ministry of Health (New Zealand).
New Zealand	2003	Ministry of Health (New Zealand). New Zealand Mortality Collection 2003. Wellington, New Zealand: Ministry of Health (New Zealand).
New Zealand	2002	Ministry of Health (New Zealand). New Zealand Mortality Collection 2002. Wellington, New Zealand: Ministry of Health (New Zealand).
New Zealand	2001	Ministry of Health (New Zealand). New Zealand Mortality Collection 2001. Wellington, New Zealand: Ministry of Health (New Zealand).
New Zealand	2000	Ministry of Health (New Zealand). New Zealand Mortality Collection 2000. Wellington, New Zealand: Ministry of Health (New Zealand).
New Zealand	1999	Ministry of Health (New Zealand). New Zealand Mortality Collection 1999. Wellington, New Zealand: Ministry of Health (New Zealand).
New Zealand	1998	Ministry of Health (New Zealand). New Zealand Mortality Collection 1998. Wellington, New Zealand: Ministry of Health (New Zealand).
New Zealand	1997	Ministry of Health (New Zealand). New Zealand Mortality Collection 1997. Wellington, New Zealand: Ministry of Health (New Zealand).
New Zealand	1996	Ministry of Health (New Zealand). New Zealand Mortality Collection 1996. Wellington, New Zealand: Ministry of Health (New Zealand).
New Zealand	1995	Ministry of Health (New Zealand). New Zealand Mortality Collection 1995. Wellington, New Zealand: Ministry of Health (New Zealand).
New Zealand	1994	Ministry of Health (New Zealand). New Zealand Mortality Collection 1994. Wellington, New Zealand: Ministry of Health (New Zealand).
New Zealand	1993	Ministry of Health (New Zealand). New Zealand Mortality Collection 1993. Wellington, New Zealand: Ministry of Health (New Zealand).
New Zealand	1992	Ministry of Health (New Zealand). New Zealand Mortality Collection 1992. Wellington, New Zealand: Ministry of Health (New Zealand).
New Zealand	1991	Ministry of Health (New Zealand). New Zealand Mortality Collection 1991. Wellington, New Zealand: Ministry of Health (New Zealand).
New Zealand	1990	Ministry of Health (New Zealand). New Zealand Mortality Collection 1990. Wellington, New Zealand: Ministry of Health (New Zealand).
New Zealand	1989	Ministry of Health (New Zealand). New Zealand Mortality Collection 1989. Wellington, New Zealand: Ministry of Health (New Zealand).
New Zealand	1988	Ministry of Health (New Zealand). New Zealand Mortality Collection 1988. Wellington, New Zealand: Ministry of Health (New Zealand).
South Africa	2016	Department of Home Affairs (South Africa), Statistics South Africa. South Africa Vital Registration - Causes of Death 2016. Pretoria, South Africa: Statistics South Africa, 2019.
South Africa	2015	Department of Home Affairs (South Africa), Statistics South Africa. South Africa Vital Registration - Causes of Death 2015. Pretoria, South Africa: Statistics South Africa.
South Africa	2014	Department of Home Affairs (South Africa), Statistics South Africa. South Africa Vital Registration - Causes of Death 2014. Pretoria, South Africa: Statistics South Africa.
South Africa	2013	Department of Home Affairs (South Africa), Statistics South Africa. South Africa Vital Registration - Causes of Death 2013. Pretoria, South Africa: Statistics South Africa.
South Africa	2012	Department of Home Affairs (South Africa), Statistics South Africa. South Africa Vital Registration - Causes of Death 2012. Pretoria, South Africa: Statistics South Africa.
South Africa	2011	Department of Home Affairs (South Africa), Statistics South Africa. South Africa Vital Registration - Causes of Death 2011. Pretoria, South Africa: Statistics South Africa.

South Africa	2010	Department of Home Affairs (South Africa), Statistics South Africa. South Africa Vital Registration - Causes of Death 2010. Pretoria, South Africa: Statistics South Africa.
South Africa	2009	Department of Home Affairs (South Africa), Statistics South Africa. South Africa Vital Registration - Causes of Death 2009. Pretoria, South Africa: Statistics South Africa.
South Africa	2008	Department of Home Affairs (South Africa), Statistics South Africa. South Africa Vital Registration - Causes of Death 2008. Pretoria, South Africa: Statistics South Africa.
South Africa	2007	Department of Home Affairs (South Africa), Statistics South Africa. South Africa Vital Registration - Causes of Death 2007. Pretoria, South Africa: Statistics South Africa.
South Africa	2006	Department of Home Affairs (South Africa), Statistics South Africa. South Africa Vital Registration - Causes of Death 2006. Pretoria, South Africa: Statistics South Africa.
South Africa	1997 – 2005	Department of Home Affairs (South Africa), Statistics South Africa. South Africa Vital Registration - Causes of Death 1997-2005. Pretoria, South Africa: Statistics South Africa.
United States	1988	National Center for Health Statistics, Centers for Disease Control and Prevention. United States NVSS Mortality Data 1988 - NBER. Hyattsville, United States: National Center for Health Statistics, Centers for Disease Control and Prevention.
United States	1987	National Center for Health Statistics, Centers for Disease Control and Prevention. United States NVSS Mortality Data 1987 - NBER. Hyattsville, United States: National Center for Health Statistics, Centers for Disease Control and Prevention.
United States	1986	National Center for Health Statistics, Centers for Disease Control and Prevention. United States NVSS Mortality Data 1986 - NBER. Hyattsville, United States: National Center for Health Statistics, Centers for Disease Control and Prevention.
United States	1985	National Center for Health Statistics, Centers for Disease Control and Prevention. United States NVSS Mortality Data 1985 - NBER. Hyattsville, United States: National Center for Health Statistics, Centers for Disease Control and Prevention.
United States	1984	National Center for Health Statistics, Centers for Disease Control and Prevention. United States NVSS Mortality Data 1984 - NBER. Hyattsville, United States: National Center for Health Statistics, Centers for Disease Control and Prevention.
United States	1983	National Center for Health Statistics, Centers for Disease Control and Prevention. United States NVSS Mortality Data 1983 - NBER. Hyattsville, United States: National Center for Health Statistics, Centers for Disease Control and Prevention.
United States	1982	National Center for Health Statistics, Centers for Disease Control and Prevention. United States NVSS Mortality Data 1982 - NBER. Hyattsville, United States: National Center for Health Statistics, Centers for Disease Control and Prevention.
United States	1981	National Center for Health Statistics, Centers for Disease Control and Prevention. United States NVSS Mortality Data 1981 - NBER. Hyattsville, United States: National Center for Health Statistics, Centers for Disease Control and Prevention.
United States	1980	National Center for Health Statistics, Centers for Disease Control and Prevention. United States NVSS Mortality Data 1980 - NBER. Hyattsville, United States: National Center for Health Statistics, Centers for Disease Control and Prevention.

Section 6 References

1 Stevens GA, Alkema L, Black RE, *et al.* Guidelines for Accurate and Transparent Health Estimates Reporting: the GATHER statement. *The Lancet* 2016; **388**: e19–23.

2 Hersbach H, Bell B, Berrisford P, *et al.* The ERA5 Global Reanalysis. *Q J R Meteorol Soc* 2020; **146**: 1999–2049.

3 Geography and Environmental Science, University of Southampton. Age and Sex Structures, Global Per Country 2000-2020 - WorldPop. Southampton , United Kingdom: Geography and Environmental Science, University of Southampton, 2018. .

4 Roth GA, Abate D, Abate KH, *et al.* Global, regional, and national age-sex-specific mortality for 282 causes of death in 195 countries and territories, 1980–2017: a systematic analysis for the Global Burden of Disease Study 2017. *The Lancet* 2018; **392**: 1736–88.

5 Johnson SC, Cunningham M, Dippenaar IN, *et al.* Public health utility of cause of death data: applying empirical algorithms to improve data quality. *BMC Medical Informatics and Decision Making* 2021; **21**: 175.

6 Zheng P, Barber R, Sorensen RJD, Murray CJL, Aravkin AY. Trimmed Constrained Mixed Effects Models: Formulations and Algorithms. *Journal of Computational and Graphical Statistics* 2021; **0**: 1–13.

7 Li Y, Johnson EK, Shi T, *et al.* National burden estimates of hospitalisations for acute lower respiratory infections due to respiratory syncytial virus in young children in 2019 among 58 countries: a modelling study. *The Lancet Respiratory Medicine* 2020; published online Sept 21. DOI:10.1016/S2213-2600(20)30322-2.

8 Lozano R, Fullman N, Mumford JE, *et al.* Measuring universal health coverage based on an index of effective coverage of health services in 204 countries and territories, 1990–2019: a systematic analysis for the Global Burden of Disease Study 2019. *The Lancet* 2020; **396**: 1250–84.

9 Rousseeuw PJ. Least Median of Squares Regression. *Journal of the American Statistical Association* 1984; **79**: 871–80.

10 Aravkin A, Davis D. Trimmed Statistical Estimation via Variance Reduction. *Mathematics of Operations Research* 2019; : moor.2019.0992.

11 Yang E, Lozano AC, Aravkin A. A general family of trimmed estimators for robust high-dimensional data analysis. *Electronic Journal of Statistics* 2018; **12**: 3519–53.

12 Roth GA, Abate D, Abate KH, *et al.* Global, regional, and national age-sex-specific mortality for 282 causes of death in 195 countries and territories, 1980-2017: a systematic analysis for the Global Burden of Disease Study 2017 GBD 2017 Causes of Death Collaborators*. 2018 DOI:10.1016/S0140-6736(18)32203-7.

13 Murray CJL, Lopez AD, Murray CL, Lopez AD. On the Comparable Quantification of Health Risks : Lessons from the Global Burden of Disease Study. 1999; **10**: 594–605.

14 Murray CJL, Aravkin AY, Zheng P, *et al.* Global burden of 87 risk factors in 204 countries and territories, 1990–2019: a systematic analysis for the Global Burden of Disease Study 2019. *The Lancet* 2020; **396**: 1223–49.

15 Stanaway JD, Afshin A, Gakidou E, *et al.* Global, regional, and national comparative risk assessment of 84 behavioural, environmental and occupational, and metabolic risks or clusters of risks for 195 countries and territories, 1990-2017: a systematic analysis for the Global Burden of Dise. 2018 DOI:10.1016/S0140-6736(18)32225-6.

16 Keatinge WR. Cold exposure and winter mortality from ischaemic heart disease, cerebrovascular disease, respiratory disease, and all causes in warm and cold regions of Europe. *Lancet* 1997; **349**: 1341–6.

17 Schneider A, Panagiotakos D, Picciotto S, *et al.* Air Temperature and Inflammatory Responses in Myocardial Infarction Survivors. *Epidemiology* 2008; **19**: 391–400.

18 Keatinge WR, Coleshaw SR, Cotter F, Mattock M, Murphy M, Chelliah R. Increases in platelet and red cell counts, blood viscosity, and arterial pressure during mild surface cooling: factors in mortality from coronary and cerebral thrombosis in winter. *British medical journal (Clinical research ed)* 1984; **289**: 1405–8.

19 Neild PJ, Syndercombe-Court D, Keatinge WR, Donaldson GC, Mattock M, Caunce M. Cold-induced increases in erythrocyte count, plasma cholesterol and plasma fibrinogen of elderly people without a comparable rise in protein C or factor X. *Clinical science (London, England : 1979)* 1994; **86**: 43–8.

20 Keatinge WR, Coleshaw SR, Easton JC, Cotter F, Mattock MB, Chelliah R. Increased platelet and red cell counts, blood viscosity, and plasma cholesterol levels during heat stress, and mortality from coronary and cerebral thrombosis. *The American journal of medicine* 1986; **81**: 795–800.

21 Keatinge WR. Cold exposure and winter mortality from ischaemic heart disease, cerebrovascular disease, respiratory disease, and all causes in warm and cold regions of Europe. *Lancet* 1997; **349**: 1341–6.

22 Keatinge WR, Donaldson GC, Bucher K, *et al.* Winter mortality in relation to climate. *International journal of circumpolar health* 2000; **59**: 154–9.

23 Koskela HO. Cold air-provoked respiratory symptoms: the mechanism and management. *International Journal of Circumpolar HealthInt J Circumpolar Health International Journal of Circumpolar Health* 2007; **66**: 91–100.

24 Burkart K, Khan MdMH, Schneider A, *et al.* The effects of season and meteorology on human mortality in tropical climates: a systematic review. *Transactions of The Royal Society of Tropical Medicine and Hygiene* 2014; **108**: 393–401.

25 Kim Y, Kim H, Gasparrini A, *et al.* Suicide and Ambient Temperature: A Multi-Country Multi-City Study. *Environmental health perspectives* 2019; **127**: 117007.

26 Tiihonen J, Halonen P, Tiihonen L, Kautiainen H, Storvik M, Callaway J. The Association of Ambient Temperature and Violent Crime. *Scientific Reports* 2017; **7**. DOI:10.1038/s41598-017-06720-z.

27 Ramsey JD. Task performance in heat: A review. *Ergonomics* 1995; **38**: 154–65.

28 Basagaña X, Sartini C, Barrera-Gómez J, *et al.* Heat Waves and Cause-specific Mortality at all Ages. *Epidemiology* 2011; **22**: 765–72.

29 Ambrose JA, Singh M. Pathophysiology of coronary artery disease leading to acute coronary syndromes. *F1000prime reports* 2015; **7**: 08.

30 Yang L, Li L, Lewington S, *et al.* Outdoor temperature, blood pressure, and cardiovascular disease mortality among 23 000 individuals with diagnosed cardiovascular diseases from China. *European Heart Journal* 2015; **36**: 1178–85.

31 Guo Y, Li S, Zhang Y, *et al.* Extremely cold and hot temperatures increase the risk of ischaemic heart disease mortality: epidemiological evidence from China. *Heart* 2013; **99**: 195–203.

32 Loughnan ME, Nicholls N, Tapper NJ. Demographic, seasonal, and spatial differences in acute myocardial infarction admissions to hospital in Melbourne Australia. *International Journal of Health Geographics* 2008; **7**: 42.

33 Bayentin L, El Adlouni S, Ouarda TBMJ, Gosselin P, Doyon B, Chebana F. Spatial variability of climate effects on ischemic heart disease hospitalization rates for the period 1989-2006 in Quebec, Canada. *International journal of health geographics* 2010; **9**: 5.

34 Neild P, Keatinge W, Donaldson G, Science MM-C, 1994 undefined. Cold-induced increases in erythrocyte count, plasma cholesterol and plasma fibrinogen of elderly people without a comparable rise in protein C or factor X. *clinsci.org*.

35 Schneider A, Panagiotakos D, Picciotto S, *et al.* Air Temperature and Inflammatory Responses in Myocardial Infarction Survivors. *Epidemiology* 2008; **19**: 391–400.

36 Guo Y, Barnett AG, Pan X, Yu W, Tong S. The Impact of Temperature on Mortality in Tianjin, China: A Case-Crossover Design with a Distributed Lag Nonlinear Model. *Environmental Health Perspectives* 2011; **119**: 1719–25.

37 Tawatsupa B, Dear K, Kjellstrom T, Sleigh A. The association between temperature and mortality in tropical middle income Thailand from 1999 to 2008. *International Journal of Biometeorology* 2014; **58**: 203–15.

38 Anderson BG, Bell ML. Weather-related mortality: how heat, cold, and heat waves affect mortality in the United States. *Epidemiology* 2009; **20**: 205–13.

39 Lian H, Ruan Y, Liang R, Liu X, Fan Z. Short-Term Effect of Ambient Temperature and the Risk of Stroke: A Systematic Review and Meta-Analysis. *International Journal of Environmental Research and Public Health* 2015; **12**: 9068–88.

40 Leon LR, Helwig BG. Heat stroke: Role of the systemic inflammatory response. *Journal of Applied Physiology* 2010; **109**: 1980–8.

41 Lian H, Ruan Y, Liang R, Liu X, Fan Z. Short-Term Effect of Ambient Temperature and the Risk of Stroke: A Systematic Review and Meta-Analysis. *International Journal of Environmental Research and Public Health* 2015; **12**: 9068–88.

42 Basagaña X, Sartini C, Barrera-Gómez J, *et al.* Heat Waves and Cause-specific Mortality at all Ages. *Epidemiology* 2011; **22**: 765–72.

43 Roy MK, Ray A. Effect of body temperature on mortality of acute stroke. *The Journal of the Association of Physicians of India* 2004; **52**: 959–61.

44 Keatinge WR, Donaldson GC, Bucher K, *et al.* Winter mortality in relation to climate. *International journal of circumpolar health* 2000; **59**: 154–9.

45 Keatinge WR, Donaldson GC, Bucher K, *et al.* Winter mortality in relation to climate. *International journal of circumpolar health* 2000; **59**: 154–9.

46 Berk JL, Lenner KA, McFadden ER. Cold-induced bronchoconstriction: role of cutaneous reflexes vs. direct airway effects. *Journal of Applied Physiology* 1987; **63**: 659–64.

47 Rau R. Seasonality in Human Mortality. A Demographic Approach. Berlin: Springer, 2006.

48 Hansel NN, McCormack MC, Kim V. The Effects of Air Pollution and Temperature on COPD. *COPD* 2016; **13**: 372–9.

49 Davie GS, Baker MG, Hales S, Carlin JB. Trends and determinants of excess winter mortality in New Zealand: 1980 to 2000. *BMC Public Health* 2007; **7**: 263.

50 Tseng C-M, Chen Y-T, Ou S-M, *et al.* The Effect of Cold Temperature on Increased Exacerbation of Chronic Obstructive Pulmonary Disease: A Nationwide Study. *PLoS ONE* 2013; **8**: e57066.

51 Koskela HO, Koskela AK, Tukiaineu HO. Bronchoconstriction due to cold weather in COPD. The roles of direct airway effects and cutaneous reflex mechanisms. *Chest* 1996; **110**: 632–6.

52 Li M, Li Q, Yang G, Kolosov VP, Perelman JM, Zhou XD. Cold temperature induces mucin hypersecretion from normal human bronchial epithelial cells in vitro through a transient receptor potential melastatin 8 (TRPM8)–mediated mechanism. *Journal of Allergy and Clinical Immunology* 2011; **128**: 626-634.e5.

53 Anderson BG, Bell ML. Weather-Related Mortality. *Epidemiology* 2009; **20**: 205–13.

54 Zanobetti A, Schwartz J. Temperature and Mortality in Nine US Cities. *Epidemiology* 2008; **19**: 563–70.

55 Braga ALF, Zanobetti A, Schwartz J. The effect of weather on respiratory and cardiovascular deaths in 12 U.S. cities. *Environmental health perspectives* 2002; **110**: 859–63.

56 Monteiro A, Carvalho V, Oliveira T, Sousa C. Excess mortality and morbidity during the July 2006 heat wave in Porto, Portugal. *International Journal of Biometeorology* 2013; **57**: 155–67.

57 Liu L, Breitner S, Pan X, *et al.* Associations between air temperature and cardio-respiratory mortality in the urban area of Beijing, China: a time-series analysis. *Environmental Health* 2011; **10**: 51.

58 Katsouyanni K, Pantazopoulou A, Touloumi G, *et al.* Evidence for Interaction between Air Pollution and High Temperature in the Causation of Excess Mortality. *Archives of Environmental Health: An International Journal* 1993; **48**: 235–42.

59 Burkart K, Canário P, Breitner S, *et al.* Interactive short-term effects of equivalent temperature and air pollution on human mortality in Berlin and Lisbon. *Environmental pollution* 2013; **183**.

60 Ren C, Williams GM, Mengersen K, Morawska L, Tong S. Does temperature modify short-term effects of ozone on total mortality in 60 large eastern US communities? — An assessment using the NMMAPS data. *Environment International* 2008; **34**: 451–8.

61 Ren C, Williams GM, Tong S. Does particulate matter modify the association between temperature and cardiorespiratory diseases? *Environmental health perspectives* 2006; **114**: 1690–6.

62 Stafoggia M, Schwartz J, Forastiere F, Perucci CA, Group S. Does Temperature Modify the Association between Air Pollution and Mortality? A Multicity Case-Crossover Analysis in Italy. *American Journal of Epidemiology* 2008; **167**: 1476–85.

63 Pattenden S, Armstrong B, Milojevic A, *et al.* Ozone, heat and mortality: acute effects in 15 British conurbations. *Occupational and Environmental Medicine* 2010; **67**: 699–707.

64 Kelsall JE, Samet JM, Zeger SL, Xu J. Air pollution and mortality in Philadelphia, 1974-1988. *American journal of epidemiology* 1997; **146**: 750–62.

65 Basu R, Feng W-Y, Ostro BD. Characterizing Temperature and Mortality in Nine California Counties. *Epidemiology* 2008; **19**: 138–45.

66 Analitis A, Michelozzi P, D'Ippoliti D, *et al.* Effects of Heat Waves on Mortality. *Epidemiology* 2014; **25**: 15–22.

67 Qian Z, He Q, Lin H-M, *et al.* High temperatures enhanced acute mortality effects of ambient particle pollution in the "oven" city of Wuhan, China. *Environmental health perspectives* 2008; **116**: 1172–8.

68 Hansel NN, McCormack MC, Kim V. The Effects of Air Pollution and Temperature on COPD. *COPD* 2016; **13**: 372–9.

69 Hayes D, Collins PB, Khosravi M, Lin R-L, Lee L-Y. Bronchoconstriction Triggered by Breathing Hot Humid Air in Patients with Asthma. *American Journal of Respiratory and Critical Care Medicine* 2012; **185**: 1190–6.

70 de Lorenzo A, Liaño F. High temperatures and nephrology: The climate change problem. *Nefrología (English Edition)* 2017; **37**: 492–500.

71 Glaser J, Lemery J, Rajagopalan B, *et al.* Climate Change and the Emergent Epidemic of CKD from Heat Stress in Rural Communities: The Case for Heat Stress Nephropathy. *Clinical Journal of the American Society of Nephrology* 2016; **11**: 1472–83.

72 Barraclough KA, Blashki GA, Holt SG, Agar JWM. Climate change and kidney disease-threats and opportunities. *Kidney international* 2017; **92**: 526–30.

73 Strippoli G, Graig JC, Rochtchina E, Flood VM, Wang JJ, Mitchell P. Fluid and nutrient intake and risk of chronic kidney disease. *Nephrology* 2011; **16**: 326–34.

74 Barraclough KA, Blashki GA, Holt SG, Agar JWM. Climate change and kidney disease-threats and opportunities. *Kidney international* 2017; **92**: 526–30.

75 STRIPPOLI GF, CRAIG JC, ROCHTCHINA E, FLOOD VM, WANG JJ, MITCHELL P. Fluid and nutrient intake and risk of chronic kidney disease. *Nephrology* 2011; **16**: 326–34.

76 Lotan Y, Daudon M, Bruyère F, *et al.* Impact of fluid intake in the prevention of urinary system diseases. *Current Opinion in Nephrology and Hypertension* 2013; **22**: S1–10.

77 Lafontan M. H4H - hydration for health. *Obesity facts* 2014; **7 Suppl 2**: 1–5.

78 Clark WF, Sontrop JM, Macnab JJ, *et al.* Urine volume and change in estimated GFR in a community-based cohort study. *Clinical journal of the American Society of Nephrology : CJASN* 2011; **6**: 2634–41.

79 Torres VE. Vasopressin in chronic kidney disease: an elephant in the room? *Kidney International* 2009; **76**: 925–8.

80 Hall ME, do Carmo JM, da Silva AA, Juncos LA, Wang Z, Hall JE. Obesity, hypertension, and chronic kidney disease. *International journal of nephrology and renovascular disease* 2014; **7**: 75–88.

81 Cook CB, Wellik KE, Fowke M. Geoenvironmental diabetology. *Journal of diabetes science and technology* 2011; **5**: 834–42.

82 Seposo XT, Dang TN, Honda Y. How Does Ambient Air Temperature Affect Diabetes Mortality in Tropical Cities? *International journal of environmental research and public health* 2017; **14**. DOI:10.3390/ijerph14040385.

83 Yang J, Yin P, Zhou M, *et al.* The effect of ambient temperature on diabetes mortality in China: A multi-city time series study. *Science of The Total Environment* 2016; **543**: 75–82.

84 Sun S, Tian L, Qiu H, *et al.* The influence of pre-existing health conditions on short-term mortality risks of temperature: Evidence from a prospective Chinese elderly cohort in Hong Kong. *Environmental Research* 2016; **148**: 7–14.

85 Lavigne E, Gasparrini A, Wang X, *et al.* Extreme ambient temperatures and cardiorespiratory emergency room visits: assessing risk by comorbid health conditions in a time series study. *Environmental Health* 2014; **13**: 5.

86 Basu R, Pearson D, Malig B, Broadwin R, Green R. The Effect of High Ambient Temperature on Emergency Room Visits. *Epidemiology* 2012; **23**: 813–20.

87 Green RS, Basu R, Malig B, Broadwin R, Kim JJ, Ostro B. The effect of temperature on hospital admissions in nine California counties. *International Journal of Public Health* 2010; **55**: 113–21.

88 Schwartz J. Who is sensitive to extremes of temperature?: A case-only analysis. *Epidemiology (Cambridge, Mass)* 2005; **16**: 67–72.

89 Lindstrom SJ, Nagalingam V, Newnham HH. Impact of the 2009 Melbourne heatwave on a major public hospital. *Internal Medicine Journal* 2013; **43**: 1246–50.

90 Medina-Ramón M, Zanobetti A, Cavanagh DP, Schwartz J. Extreme temperatures and mortality: Assessing effect modification by personal characteristics and specific cause of death in a multi-city case-only analysis. *Environmental Health Perspectives* 2006; **114**: 1331–6.

91 Vaneckova P, Bambrick H. Cause-Specific Hospital Admissions on Hot Days in Sydney, Australia. *PLoS ONE* 2013; **8**: e55459.

92 Zanobetti A, O'Neill MS, Gronlund CJ, Schwartz JD. Susceptibility to Mortality in Weather Extremes. *Epidemiology* 2013; **24**: 809–19.

93 Li Y, Lan L, Wang Y, *et al.* Extremely cold and hot temperatures increase the risk of diabetes mortality in metropolitan areas of two Chinese cities. *Environmental Research* 2014; **134**: 91–7.

94 Westphal S, Childs R, Seifert K, *et al.* Managing Diabetes in the Heat: Potential Issues and Concerns. *Endocrine Practice* 2010; **16**: 506–11.

95 Scott AR, MacDonald IA, Bennett T, Tattersall RB. Abnormal thermoregulation in diabetic autonomic neuropathy. *Diabetes* 1988; **37**: 961–8.

96 Kenny GP, Sigal RJ, McGinn R. Body temperature regulation in diabetes. *Temperature (Austin, Tex)* 2016; **3**: 119–45.
